# Towards medical imaging of drug photoactivation: Development of light responsive magnetic resonance imaging and chemical exchange saturation transfer contrast agents

**DOI:** 10.1002/smo.20230029

**Published:** 2024-06-01

**Authors:** Ilse M. Welleman, Carlijn L. F. van Beek, Ioana Belcin, Albert M. Schulte, Rudi A. J. O. Dierckx, Ben L. Feringa, Hendrikus H. Boersma, Wiktor Szymański

**Affiliations:** ^1^ Department of Radiology Medical Imaging Center University Medical Center Groningen University of Groningen Groningen The Netherlands; ^2^ Stratingh Institute for Chemistry University of Groningen Groningen The Netherlands; ^3^ Department of Clinical Pharmacy and Pharmacology Nuclear Medicine and Molecular Imaging University Medical Center Groningen University of Groningen Groningen The Netherlands

**Keywords:** CEST, MRI, photochemistry, photocleavable protecting groups, photopharmacology

## Abstract

In recent years, the use of light to selectively and precisely activate drugs has been developed along the fundamental concepts of photopharmacology. One of the key methods in this field relies on transiently silencing the drug activity with photocleavable protecting groups (PPGs). To effectively utilize light‐activated drugs in future medical applications, physicians will require a reliable method to assess whether light penetrates deep enough into the tissues to activate the photoresponsive theragnostic agents. Here, we describe the development and evaluation of magnetic resonance (MR) imaging agents that allow for the detection of light penetration and drug activation in the tissues using non‐invasive whole‐body magnetic resonance imaging (MRI) and chemical exchange saturation transfer (CEST)‐MRI modalities. The approach relies on the use of PPG‐protected MR contrast agents, which upon irradiation with light change their imaging signal. A Gadolinium(III)‐based MRI contrast agent is presented that undergoes a significant change in relaxivity (25%) upon uncaging, providing a reliable indicator of light‐induced cargo release. Additionally, we introduce the first light‐responsive CEST‐MRI imaging agent, enabling positive signal enhancement (off‐to‐on) upon light activation, offering a novel approach to visualize the activation of photoactive agents in living tissues. This research provides a proof‐of‐principle for the non‐invasive, whole‐body imaging of light penetration and drug activation with high temporal resolution characteristic of MR methods.

## Graphical abstract

Seeing the light inside: In this paper, smart contrast agents are presented that enable the use of non‐invasive whole‐body methods for the imaging of light penetration and photoactivation of drugs inside the human body. These molecules change their MRI and CEST‐MRI signal under light irradiation, acting as responsive imaging agents for the future applications of light in medicine, photodynamic therapy and photopharmacology.

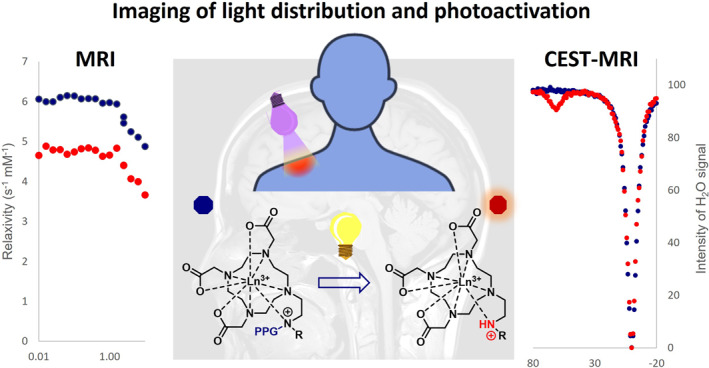

## 1 INTRODUCTION

Light's unique properties, such as high spatiotemporal precision, control over intensity and wavelength, and low cytotoxicity, make it a powerful tool for biomedical applications. Light plays a major role in clinical diagnostics, serving as both a readout signal and an excitation stimulus in various optical and optoacoustic imaging techniques.^[1]^ In addition to its use in diagnostics, light can also be used in cancer treatment through photodynamic therapy (PDT). In PDT, photosensitizing drugs are administered to the patient and upon irradiation with light reactive oxygen species are formed that damage and kill cancer cells.^[2]^ Recently, other methods to activate drugs with light are emerging, relying either on light‐induced release from carriers or on a small‐molecule approach of photopharmacology. In the latter approach, drugs are either caged by photocleavable protecting groups (PPGs), transiently masking their bioactivity, or functionalized with molecular photoswitches to enable reversible control over the bioactivity of the drug.[^3^, ^4^, ^5^, ^6^]

In future clinical applications of light in medicine, physicians will need reliable methods to determine whether light penetrates deep enough into the tissues to effectively activate the photoresponsive agents. Furthermore, in scenarios in which light for therapeutic purposes would be generated inside the human body, for example, through luminescent systems like luciferin/luciferase that have been envisioned for this purpose,^[7]^ imaging of the light distribution[^7^, ^8^, ^9^, ^10^, ^11^] and intensity will be required. In this context, a light‐responsive whole‐body medical imaging technique would offer a safe and effective approach to visualize the distribution of light and the progress of drug activation within the body. Those characteristics are very well fulfilled by magnetic resonance imaging (MRI), which is a non‐invasive, whole‐body imaging technique with outstanding spatial and temporal resolution.[^12^, ^13^] Hence, MRI is perfectly suited for imaging the activation of photoresponsive molecules, provided that they can function as stimuli‐responsive MRI contrast agents (MRI CAs).

Traditional MRI CAs are molecules that enhance the contrast by shortening the longitudinal and/or transverse relaxation times of neighboring protons in the tissue and altering the intensity of a specific tissue or structure. This enables the differentiation from surrounding tissues, thereby helping the physicians with the diagnosis of the disease.^[14]^ In recent years, light‐responsive MRI CAs have been emerging that exhibit outstanding photopharmacological properties. They could therefore in principle be used to determine whether light penetrates deep enough into the tissues to effectively activate the photoresponsive agents. Imaging the distribution of the light‐responsive liposomal drug delivery system and the drug release from it has been shown by Reeβing et al.^[8]^ and more recently by Simon et al.^[9]^ In addition, MRI CAs with incorporated photoswitches have been published, where the light‐induced change in the configuration induces a positive difference in MRI signal: Dommaschk et al.[^10^, ^11^] showed the application of an azobenzene photoswitch, while Kruttwig et al.^[7]^ showed the same concepts for a spiropyran photoswitch.

Recently, our group has attempted to design an MRI CA that features a PPG and upon irradiation with violet (*λ* = 400 nm) light, release an MRI contrast agent, inducing a negative difference in the MRI signal. The development of “positive” contrast agents, characterized by signal amplification upon activation, presents a challenge. Predicting the factors that give rise to relaxivity enhancement proves to be complicated due to the potential for contrast agents to interact with proteins or undergo aggregation leading to relaxivity alterations.[^14^, ^15^]

A promising alternative way to design a “positive” contrast agent is to use a different MRI technique, namely, chemical exchange saturation transfer (CEST) imaging. This emerging MRI modality relies on a different mechanism, since the contrast arises due to the proton exchange between the contrast agent and the bulk water molecules surrounding it. Recent advancements have demonstrated that CEST contrast agents have potential for the imaging of physiological parameters, including pH,^[16]^ temperature^[17]^ and enzyme activity.^[18]^

Here we introduce PPG‐bearing CAs for both magnetic resonance (MR) imaging modalities that respond to light by releasing a cargo and thereby changing their imaging signal in our attempts towards imaging the light penetration and activation of photopharmacological agents. In the design, we use classical and widely used classes of PPGs that respond to UV‐violet light, showing a proof‐of‐principle for future imaging agents activated by external irradiation with deep‐tissue‐penetrating red light, but also establishing systems directly applicable in scenarios involving light generation inside the body. We present a Gadolinium(III)‐bearing MRI CA that reports on its uncaging by changing its relaxivity by 25%. Furthermore, we present the first example of a CEST‐MRI imaging agent that is responsive to light and enables positive (off‐to‐on) signals.

## 2 RESULTS AND DISCUSSION

### 2.1 Design

**FIGURE 1:**
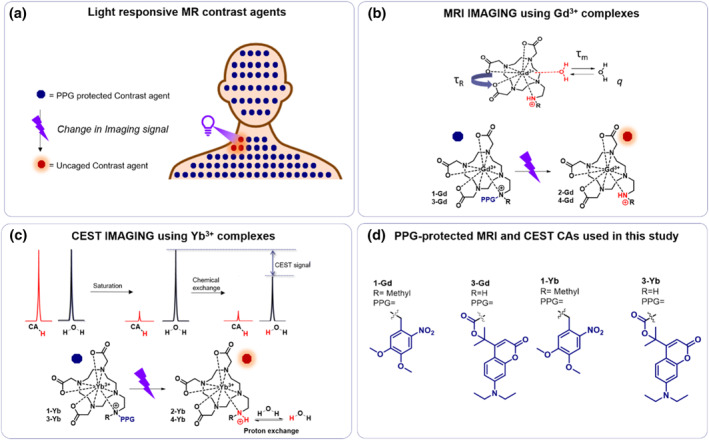
Schematic representation of the functioning of light responsive CAs for imaging the light penetration and activation of a model photocaged drug. (a) Upon irradiation with light, the light responsive CAs creates a change in the MR imaging signal. (b) Schematic explanation of activatable MRI CA design and the synthesized molecules **1‐Gd** and **3‐Gd**: upon irradiation with light, the photocleavable protecting group is removed and the newly formed contrast agents **2‐Gd** and **4‐Gd** show a difference in relaxivity. (c) Schematic explanation of the CEST‐MRI working principle and the designed molecules **1‐Yb** and **3‐Yb**, upon irradiation the photocleavable protecting group is removed and the newly formed contrast agents **2‐Yb** and **4‐Yb** have protons to participate in CEST. (d) The outline of the CA structures presented in this work with different R‐groups and PPGs attached.

The schematic application of the photocleavable PPG‐protected MRI/CEST contrast agent is shown in Figure 1a. For the contrast agent to effectively report on the removal of the PPG, the PPG's release must generate a distinguishable change in the MR signal. In order to create a difference in signal for traditional MRI (i.e., magnetic relaxivity), one of the following structural parameters that define the relaxivity of an MRI contrast agent needs to be changed upon release of the PPG (Figure 1b): (1) the number of water molecules binding to the metal center (*q*), (2) the residence time of the coordinated water molecule on to the lanthanide complex (*τ*_*m*_), or (3) the rotational correlation time of a molecule (*τ*_*R*_).^[14]^

On the other hand, a different set of requirements is applicable to CEST‐MRI. CEST is a technique that utilizes the exchange of protons between a contrast agent and the bulk water protons in the tissues surrounding it. The CEST image is created by applying a radiofrequency pulse to saturate the protons from the contrast agent, whereupon the saturated proton pool of the contrast agent exchanges with the bulk water protons, thereby transferring the saturation. This process reduces the signal of bulk water, which is detected and transformed to an image (Figure 1c).[^14^, ^19^] In order to generate a change in CEST MRI imaging, the exchange of the protons needs to be controlled.

The design of the light‐responsive MRI/CEST‐MRI contrast agents is shown in Figure 1b,c. Lanthanide‐based contrast agents were selected because of their established clinical applicability.^[14]^ To complex the lanthanide ion, a DOTA‐based ligand was employed. This ligand was rendered photoresponsive by replacing one of the four acetic acid arms with an aminoethylene group, which could be masked by a PPG moiety. The choice of amine functional group was inspired by a large number of drugs where an amine functional group is caged by a PPG, enabling the light‐control over the bioactivity of the drug.[^20^, ^21^, ^22^, ^23^, ^24^] Therefore, by masking an amine functional group on the contrast agent, we aimed to mimic the typical architecture of the photoactivated drug and therefore use the CA to image the amine‐bearing drug release.

Upon irradiation with light, the PPG is removed, thereby exposing the amine functional group, which is anticipated to induce a change in MR signal. For conventional MRI, this group is expected to coordinate to the lanthanide center, altering the number of water molecules that can bind to the lanthanide. In CEST‐MRI imaging, the released amine group will possess protons capable of chemical exchange with neighboring water molecules, leading to a detectable signal.

To develop a model contrast agent for the imaging of light penetration and drug activation in the human body, particularly for drugs caged by PPGs, we introduced two widely applied classes of PPGs in the contrast agent design (Figure 1d). This approach allowed us to explore the feasibility of employing this technique as a general method for imaging the efficacy of the removal of PPG from the drugs in the human body. The first PPG chosen was the 4,5‐dimethoxy‐2‐nitrobenzyl‐based protecting group (DMNB). The DMNB belongs to a class of UV‐light activated nitrobenzyl PPGs,^[25]^ which is widely applied in preparing photoactivated cancer chemotherapeutics^[26]^ and enzyme inhibitors.[^27^, ^28^, ^29^] For the second PPG, we have chosen a so‐called tertiary coumarin PPG, recently introduced by our group, which belongs to another extensively employed class of coumarin PPGs,^[30]^ commonly applied in neurochemistry^[31]^ and cancer chemotherapeutics.^[32]^ The tertiary coumarin can be removed under irradiation with 400 nm light and it was designed to have a high uncaging quantum yield (*φ*, QY) through stabilizing the incipient contact ion pair through hyperconjugation.^[33]^ Uncaging efficiency is of key importance because light‐penetration inside the human body is limited. Therefore, an efficient translation of photon‐absorption into payload release is crucial. To further increase the uncaging efficiency, we connected this PPG to the MRI CA amine group using a carbamate linker, resulting in what we expected to be a fast light‐responsive contrast agent,^[33]^ and allowing us to study the activation of amines through the two most often used PPG attachment approaches (direct attachment and through a carbamate linker).

### 2.2 Synthesis

**SCHEME 1:**
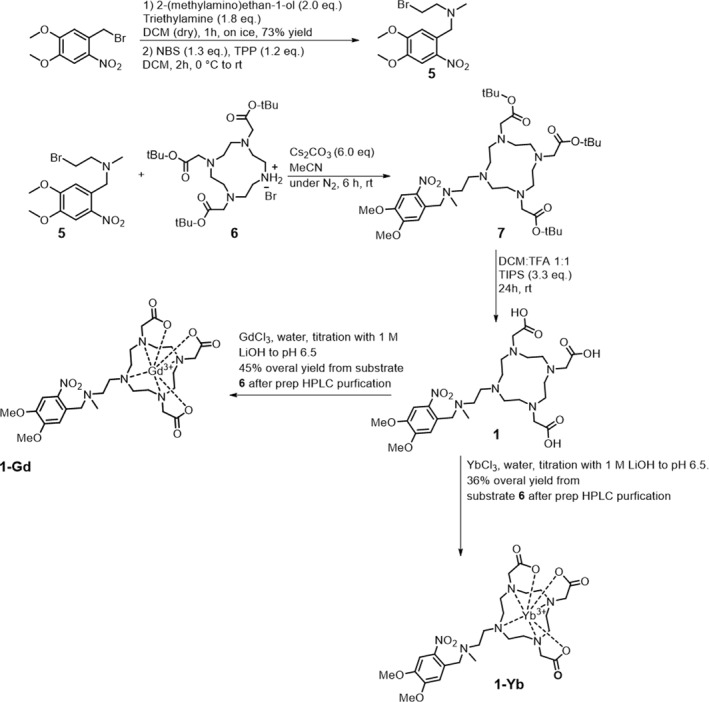
Synthetic route toward compounds **1‐Gd** and **1‐Yb**.

**SCHEME 2:**
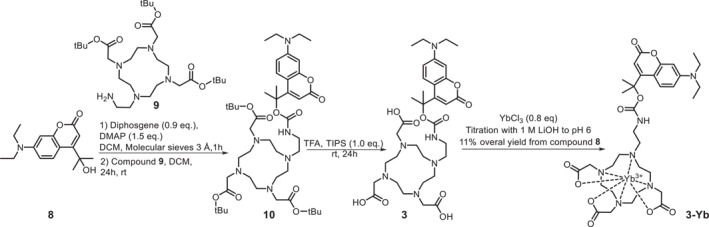
Synthetic route towards compound **3‐Yb**.

The synthetic routes for ligands **1** and **3** are shown in Schemes 1 and 2, respectively. The synthesis of both compounds consists of five steps: (1) the synthesis of the light‐responsive moiety (building block 1), (2) the synthesis of the protected chelator ligand (building block 2), (3) the coupling of the two building blocks, (4) deprotection of the ester moieties from the DOTA ligand and (5) complexation of the lanthanide metal and final preparative HPLC purification.

(Detailed procedures for all experiments are provided in the Supporting Information Section S2, summarized in Figures S1 and S2. All characterization data for the compounds described in the main text can be found in Figures S19–S57).

The synthesis of the first building block of ligand **1** (Scheme 1) started with a nucleophilic substitution reaction leading to the formation of an intermediate alcohol using conditions inspired by a similar synthesis by Jiang et al.^[34]^ This was followed by an Appel reaction to convert the intermediate alcohol to the corresponding bromide **5**. However, the purification of compound **5** was complicated by the presence of triphenylphosphine oxide; therefore, after extensive purification of the analytical sample, crude compound **5** was used in the next step. In parallel, compound **6** was synthesized according to a published procedure^[35]^ (see Supporting Information Section S2) and connected to linker **5** in a nucleophilic substitution reaction. Screening of the reaction conditions resulted in a procedure that afforded excellent product selectivity towards mono alkylation at room temperature (see Supporting Information Section S2). While complete conversion was not achieved, in the next step, the *tert*‐butyl esters were successfully cleaved under acidic conditions, yielding the desired ligand **1**. However, purifying ligand **1** posed a significant challenge due to impurities formed by the deprotection of unreacted compound **6**. To circumvent the purification hurdle resulting from the very high polarity of all the compounds involved, we introduced lanthanide metals prior to purification. This strategy effectively reduced the polarity of the product, facilitating the isolation of the complexes **1‐Gd** and **1‐Yb** via preparative‐HPLC.

The synthesis of ligand **3** (Scheme 2) started with the formation of the self‐immolative carbamate linker between the previously published PPG **8** ^[33]^ and ligand **9** ^[36]^ (see Supporting Information Section S2), by first reacting compound **8** with diphosgene, and afterward adding compound **9.** This order was used to minimize unreacted compound **9**, which would later make the purification more challenging. In the next step, *tert*‐butyl esters were cleaved under acidic conditions yielding ligand **3**. While the purification of ligand **3** was rather straightforward, we have observed that inserting the lanthanide metal after purification under basic conditions resulted in addition of water to the coumarin PPG, rendering it unresponsive to light. This issue could unfortunately not be resolved by inserting the metal first and then purifying the complex via preparative HPLC. Even when **3‐Yb** was successfully synthesized and the complex purified upon incubation in water, it was not stable over time. Despite several attempts to isolate **3‐Gd**, the complex exhibited even more stability issues in comparison to **3‐Yb** and could never be isolated pure (see Figure S55). Consequently, the evaluation of **3‐Gd** was discontinued due to its inherent instability. Inspired by literature reports, we hypothesize that the lanthanide probably acts as a Lewis acid, making the coumarin more susceptible to nucleophilic attack by water.[^37^, ^38^, ^39^]

### 2.3 Photochemical evaluation of light responsive contrast agents

**FIGURE 2:**
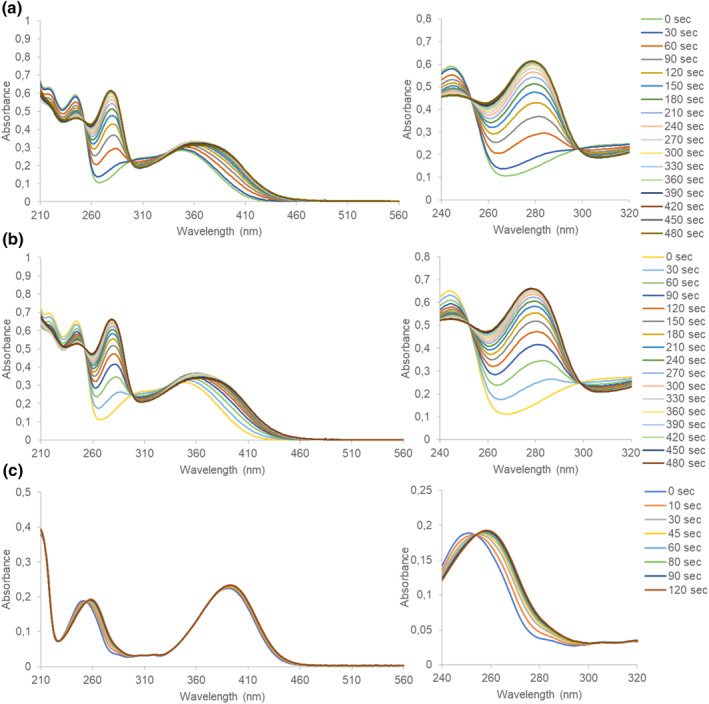
Photochemical evaluation of the uncaging process of compounds **1‐Yb**, **1‐Gd** and **3‐Yb**, with the overall spectrum shown in the left and an expanded spectrum, highlighting the isosbestic points and most pronounced signal change, shown on the right side of each panel. (a) UV‐Vis absorption spectra of **1‐Yb** (water, 100 μM, 25°C, pH 7), freshly prepared solutions, and solutions after irradiation (*λ* = 365 nm) for the times indicated in figure. (b) UV‐Vis absorption spectra of **1‐Gd** (water, 100 μM, 25°C, pH 7), freshly prepared solutions, and solutions after irradiation (*λ* = 365 nm) for the times indicated in figure. (c) UV‐Vis absorption spectra of **3‐Yb** (water, 20 μM, 25°C, pH 7), freshly prepared solutions, and solutions after irradiation (*λ* = 390 nm) for the times indicated in figure. For an expanded spectrum, see Figures S16–S18.

With the designed light responsive contrast agents in hand, we investigated their photochemical properties using UV‐Vis spectroscopy (Figure 2). The obtained absorption spectra are in line with those published for the chosen PPGs.[^28^, ^33^] Irradiation (*λ* = 365 nm) of compounds **1‐Yb** and **1‐Gd** in water resulted in changes in absorption spectra within minutes. However, upon irradiation (*λ* = 390 nm) of compound **3‐Yb** in water, we saw changes in the absorption spectrum within seconds. The observed difference of uncaging efficiency between the tertiary coumarin and DMNB is consistent with the higher efficiency of uncaging for the former, which depends on the improved quantum yield and absorptivity of the PPG.

### 2.4 Using NMR to follow light activation: Fast field cycling NMR relaxometric analysis of MRI contrast agent

Having confirmed the light‐responsive behavior of the designed contrast agents, we proceeded to evaluate the performance of **1‐Gd** in terms of proton relaxation rate enhancement (i.e., MRI signal) under light irradiation. We therefore collected a nuclear magnetic relaxation dispersion profile (NMRD) using Fast Field Cycling (FFC) relaxometry. With this method, the relaxivity (*r*_1_ = 1/*T*_1_) of contrast agents is measured over a range of magnetic fields to gain insights into their magnetic properties such as the number of water molecules coordinated to the metal ion, the water exchange rate and the rotational correlation time of the complex as these parameters all influence the shape of the NMRD profile.^[40]^ To obtain quantitative data on the light‐induced uncaging process, the relaxation of the light responsive contrast agents were measured before and after irradiation (see Tables S1–S4 for obtained data), and the conversion of the compounds was also followed with UPLC‐MS (see Figures S3–S5).

**FIGURE 3:**
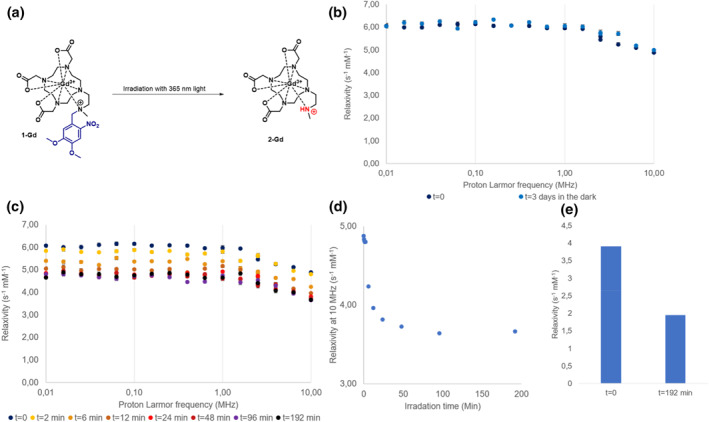
NMRD relaxometric analysis of **1‐Gd** (1.0 mM) in water at 37°C, pH 7.4. Error bars represent the uncertainty of fitting the *T*_1_ curve to the experimental data. (a) The activation of **1‐Gd** with irradiation of 365 nm light towards **2‐Gd**. (b) NMRD profile of **1‐Gd** at *t* = 0 and after 3 days in the dark (c) NMRD profiles of **1‐Gd** upon irradiation of the sample with light (*λ* = 365 nm). (d) The decrease in the relaxivity of **1‐Gd** at 10 MHz in response to irradiation with light (*λ* = 365 nm) for the indicated times. (e) The molar relaxivity of **1‐Gd** with and without light irradiation (*λ* = 365 nm) at time points *t* = 0 min and *t* = 192 min, at 4.7 T.

The NMRD profile of compound **1‐Gd** is presented in Figure 3, showing a typical shape for small molecule contrast agents, with a plateau at low fields and a drop in relaxivity in the 1–10 MHz range.^[40]^ We did not observe major changes in the NMRD profile upon incubation in water in the dark for up to 72 h at 37°C, demonstrating the excellent stability of **1‐Gd** in the absence of light (Figure 3b). However, when the sample was irradiated with UV‐light (*λ* = 365 nm), we observed an overall change in relaxivity across the entire studied range of magnetic fields (Figure 3c). This finding is consistent with the anticipated reduction of the number of water molecules that can coordinate to the metal center due to the uncaging of **1‐Gd** to **2‐Gd**. At 10 MHz, the total decrease in relaxivity from 4.88 s^−1^ mM^−1^ to 3.66 S^−1^ mM^−1^ was measured, giving a 25% change in total (Figure 3d, Table S6). Our findings align with previous work from our group by Reeβing et al.,^[41]^ who reported a 17% reduction in relaxivity upon activation of a light‐responsive contrast agent. Furthermore, we measured the relaxivity of the MRI contrast agent at 4.7 T magnetic field (Figure 3e, Table S7), which is a more representative of the magnetic field strengths used in (pre)clinical application (1.5–7 T). In this magnetic field, we were delighted to observe an even higher difference in relaxivity between **1‐Gd** and **2‐Gd** (∼50%).

The relaxivity of compound **2‐Gd** has been shown to depend on the protonation state of the amine (pK_a_ was estimated to be 7.5).[^42^, ^43^] Since we measured at pH conditions close to the pK_a_ of compound **2‐Gd**, we closely followed the pH during the NMRD measurements (see Table S5). The slight change in pH (from pH 7.40 at *t* = 0 min to pH 7.37 at *t* = 96 min) can be attributed to the products formed during the uncaging process of the DMNB cage.[^25^, ^44^] Using Henderson‐Hasselbalch equation, we estimated the associated change in the concentration of protonated **2‐Gd** to be <5%. At the same time, the observed difference in relaxivity was 25%, which led us to exclude the possibility that the change in relaxivity is caused by the change in pH.

### 2.5 Using CEST to follow light activation: Analysis of the *Z*‐spectra.

After having observed a difference in the MRI relaxivity that was indicative of light activation of the photo‐responsive contrast agent **1‐Gd**, we proceeded to investigate the performance of **1‐Yb** and **3‐Yb** as CEST‐MRI imaging agents by collecting their light‐dependent *Z*‐spectra. During the collection of a *Z*‐spectrum, a specific radiofrequency is applied every 1 ppm over a wide range of Larmor frequencies (typically between −100 and +100 ppm). The spectrum features a prominent signal at 0 ppm corresponding to the water signal. Additionally, if the protons of the contrast agents are available to exchange with the water protons, peaks originating from these protons are anticipated to appear in the spectrum.^[19]^

**FIGURE 4:**
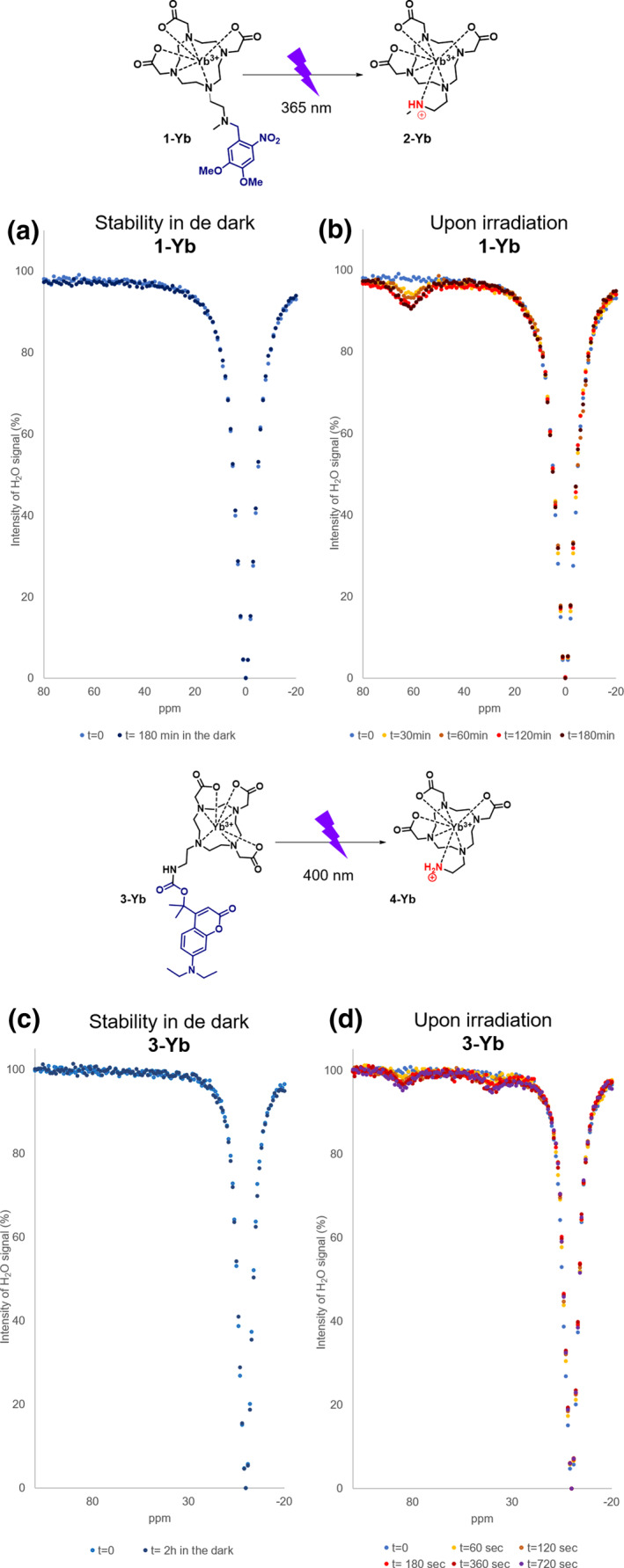
The NMR‐Z profiles for **1‐Yb** and **3‐Yb**. (a) *Z*‐spectra of the solutions of **1‐Yb** at *t* = 0 and *t* = 3 h incubation at 37°C in the dark (50 mM in water with 10% D_2_O, pH 7.40, *B*0 = 11.7 T, satpwr = 28 dB, satdly = 2 s). (b) *Z*‐spectra of the solution of **1‐Yb** at *t* = 0 min, and after irradiation (*λ* = 365 nm) for *t* = 30, 60, 120 and 180 min at 37°C (50 mM in water with 10% D_2_O, pH 7.40, *B*0 = 11.7 T, satpwr = 28 dB, satdly = 2 s). (c) *Z*‐spectra of the solutions of **3‐Yb** at *t* = 0 and *t* = 2 h at 37°C in the dark (30 mM in water with 10% D_2_O, pH 7.40, *B*0 = 11.7 T, satpwr = 28 dB, satdly = 2 s). (d) *Z*‐spectra of the solution of **3‐Yb** at *t* = 0 s, and after irradiation (*λ* = 400 nm) *t* = 60, 120, 180, 360 and 720 s at 37°C (30 mM in water with 10% D_2_O, pH 7.40, *B*0 = 11.7 T, satpwr = 28 dB, satdly = 2 s). The complete spectra are presented in the Supporting Information file (Figures S6–S7 for **1‐Yb** and Figures S10 and S11 for **3‐Yb**).

In Figure 4, the NMR‐*Z* spectra of **1‐Yb** are shown, where in Figure 4a stability of the compound is confirmed, as no new signals were observed after incubation for 3 h in the dark in water. However, after irradiation with light (*λ* = 365 nm, Figure 4b), a signal at ∼60 ppm is emerging, which originates from the exchange of a proton of the amine group, as was shown before by Krchová et al. for compound **2‐Yb.** ^[45]^ The uncaging process in the studied sample was also confirmed using UPLC‐MS analysis (see Figures S8 and S9).

For **3‐Yb**, the NMR‐Z spectra are shown in Figure 4c,d. No signal was observed to emerge in the dark during incubation of up to 2 h (Figure 4c). However, to our delight, upon irradiation with light (*λ* = 400 nm), two signals formed rapidly (within seconds), centered around ∼40 and ∼90 ppm (Figure 4d), which originate from the protons of the amine, as indicated by Krchová et al.,^[45]^ although the intensity of **3‐Yb** is lower than **1‐Yb** due to the side products formed by the attack of water on the coumarin moiety. The uncaging process was also confirmed using UPLC‐MS analysis (see Figures S12 and S13). Finally, we measured the Z‐spectra of compound **4‐Yb** under different pH conditions (see Figure S62). Since in certain situations (including tumor or infections) the pH is lowered, we measured **4‐Yb** at pH 6 and 7 and found that the amine we uncage still shows signals around ∼40 and ∼90 ppm, as also reported by Krchová et al.^[45]^

### 2.6 Stability of the light‐responsive contrast agents

To test the stability of the complexes presented in this work, and to rule out that the observed difference in relaxivity and arising CEST signal is coming from liberation of the lanthanide ion from the complex, we performed a colorimetric assay to assess the free Gd^3+^ and Yb^3+^ ions in the solution before and after irradiation with light. The assay confirmed that there is no significant release of Gd^3+^ or Yb^3+^ into the solution (see Supporting Information Section S5, Tables S8 and S9, Figures S14 and S15). Besides the colorimetric assay, we also performed the initial stability test in a more biologically relevant medium. Inspired by the studies performed by Duimstra et al.^[46]^ on related enzyme activated derivatives of compound **4‐Gd**, we incubated the solution of **1‐Gd**, **1‐Yb** and **3‐Yb** in human plasma‐like medium (HPLM) and performed a UPLC‐MS analysis before and after 24 h of incubation at 37°C (see Supporting Information Section S10, Tables S10–S12 and Figures S58 and S59). For the contrast agents **1‐Gd** and **1‐Yb,** we observed that >95% of the intact molecule was still present in the solution after 24 h incubation. Conversely, **3‐Yb** was unstable upon incubation at 37°C for 24 h (see Supporting Information Section S10, Table S12 and Figure S60), although we observed that its decomposition was not due to the release of the PPG from the CA itself, and therefore did not interfere with the CEST signal (Figure S60). However, when the solution of **3‐Yb** in HPLM was irradiated (*λ* = 400 nm) after 24 h incubation at 37°C, it did not result in the removal of the PPG. To eliminate the possibility of HPLM interfering with the uncaging process of the PPG, a freshly prepared solution of **3‐Yb** in HPLM was irradiated with light (*λ* = 400 nm), and to our delight, the CA was liberated within seconds (Supporting Information Section S10, Figure S61). This suggests that the incubation of **3‐Yb** in HPLM induces the formation of a light unresponsive molecule on the timescale of hours.

## 3 CONCLUSION

In summary, we designed, synthesized, and evaluated two model light responsive contrast agents for the determination of light penetration and drug activation via MRI and CEST imaging. Upon irradiation with light, a substantial decrease in relaxivity was observed (25% at 0.23T, 50% at 4.7T), making **1‐Gd** a turn‐off agent. Conversely, an off–to‐on activation of the signal could for the first time be obtained using the CEST effect when the Yb^3+^ complexes **1‐Yb** and **3‐Yb** were employed. Future research will prioritize the incorporation and evaluation of novel photocages that exhibit light absorption in the near‐infrared (NIR) region of the spectrum.[^47^, ^48^, ^49^, ^50^] This focus stems from the restricted skin penetration of ultraviolet (UV) light, which hinders the activation of the photocages presented in this study deep inside the human body. Additionally, the preclinical characteristics of these molecules, encompassing their ADME profile, and toxicity, will be thoroughly investigated.

## CONFLICT OF INTEREST STATEMENT

The authors declare no conflicts of interest.

## ETHICS STATEMENT

No animal or human experiments were involved in this study.

## Supporting information

Supporting Information S1

## Data Availability

Data are contained within the article and the supplementary materials. The raw data are available on request from the corresponding author.
