# Supplementary material for: Towards medical imaging of drug photoactivation: Development of light responsive magnetic resonance imaging and chemical exchange saturation transfer contrast agents
Source: Smart Mol. 2024 Jun 1;2(2):e20230029. doi: 10.1002/smo.20230029 (PMC12118296; doi:10.1002/smo.20230029)
Supplement: Supplementary file 1 — Supporting Information S1 [file SMO2-2-e20230029-s001.docx]

Supporting Information

The development of light responsive MRI and CEST contrast agent

Ilse. M. Welleman ,Carlijn L. F van Beek, Ioana Belcin, Albert M. Schulte, Rudi A. J. O. Dierckx, Ben L. Feringa, Hendrikus H. Boersma and Wiktor Szymański*

1. General information

All solvents used for extraction, filtration and chromatography were of commercial grade, and used without further purification. Reagents were purchased from Sigma-Aldrich, TCI, Boom and Combi-Blocks and were used without further purification. For purification with column chromatography silica gel from Macherey-Nagel (Silica 60 M, 0.04-0.063 nm, 230-400 mesh) was used.

The TLC analysis were performed on Merck silica gel 60, 0.25 mm plates and stained with potassium permanganate stain (a mixture of KMnO_4_ (3 g), K_2_CO_3_ (10 g), water (300 mL)) or Seebach’s stain (a mixture of phosphomolybdic acid (2.5 g), Ce(SO_4_)2 (1 g), H_2_SO_4_ (con 6 ml) and water 94 ml) or were visualized under a UV-lamp (λ = 254 nm).

NMR spectra were obtained using Agilent Technologies 400 MR (400/54 Premium Shielded) (^1^H: 400 MHz, ^13^C: 101 MHz) using CDCl_3_, (CD_3_)_2_SO or D_2_O as solvent. Chemical shift values are reported in ppm with the solvent resonance as the internal standard (CDCl_3_: δ 7.26 for ^1^H, δ 77.16 for ^13^C, (CD_3_)_2_SO: δ 2.50 for ^1^H and δ 39.52 for ^13^C and D_2_O: δ 4.79 for 1H). Data are reported as follows: chemical shifts (δ), multiplicity (s = singlet, d = doublet, t = triplet, q =quartet, br = broad, m = multiplet), coupling constant J (Hz), integration.

High Resolution Mass measurements were performed using a Thermo Scientific LTQ OribitrapXL spectrometer.

The determination of free Yb^3+^ and Gd^3+^ concentration was performed using a Synergy H1 microplate reader from Agilent Biotek.

NMRDs were recorded on a Stelar 0.25T FFC SMARtracer relaxometer from Stelar.

Relaxivity at 4.7 Tesla was recorded on a Varian Oxford 200 MHz.

UPLC–MS analysis was performed using:

1. ThermoFisher Scientific Vanquish UPLC System (Waltham, MA, USA) with a reversed phase C18 column (Acquity UPLC BEH C18 (1.7 µm, 2.1 150 mm) in combination with an LCQ Fleet mass spectrometer and UV–vis detector at 254 nm.

**Program 1**: Eluent A: acetonitrile Eluent B: water, both with 0.1% v/v formic acid added. Program 0.0-2.0 min 5% of eluent A, 2.0-10.0 min gradient 5-90% eluent A, 10.0-10.1 min gradient 90-100 % of eluent A, 10.1-12.0 min flush of 100% A, 12.0-18.0 min gradient 100-5% eluent A.

2. Acquity UPLC-MS with TQD fitted with a BEH C18 column (1.7 uM 2.1 x 50 mm).

**Program 2**: Eluent A: acetonitrile Eluent B: water, both with 0.1% v/v formic acid added. Program 0.0-2.0 min 5% of eluent A, 2.0-10.0 min gradient 5-90% eluent A, 10.0-10.1 min gradient 90-100 % of eluent A, 10.1-15.0 min flush of 100% A, 15.0-20.0 min gradient 100-5% eluent A.

**Program 3:** Eluent A: acetonitrile Eluent B: water, both with 0.1% v/v formic acid added. Program 0.0-2.0 min 2% of eluent A, 2.0-10.0 gradient 2-90% eluent A, 10.0-10.1 min gradient 90-100 % of eluent A, 10.1-15.0 min flush of 100% A, 15.0-20.0 min gradient 100-2% eluent A.

3. Preparative HPLC purification was performed on a Shimadzu HPLC system with a Phenomenex® Kinetex 5 μm EVO C18 100 Å column, using

**Program 4**: Eluent A: acetonitrile Eluent B: 10 mM triethylammonium acetate in water. Program: 0-4 min: 2% eluent A, 4-20 min: gradient 2-25% eluent A, 20-21 min: gradient 25-100% eluent A, 21-24 min: 100 % eluent A, 24-25 min: gradient 100-2% of eluent A, 25-30 min: 2% A.

**Program 5**: Eluent A: acetonitrile Eluent B: 10 mM triethylammonium acetate in water. Program: 0-4 min: 10 % eluent A, 4-15 min: gradient 10-50% eluent A, 15-20 min: gradient 50-95% eluent A, 20-21 min: gradient 95-100% eluent A, 21-24 min: 100 % eluent A, 24-25 min: gradient 100-2% of eluent A, and from 25-30 min 10% of eluent A.

**Program 6**: Eluent A: acetonitrile Eluent B: water, both with 0.1% v/v formic acid added. Program: 0-4 min: 10% eluent A, 4-15 min gradient 10-50% eluent A, 15-20 min gradient 50-95% eluent A, 20-21 min: gradient 95-100% eluent A, 21-24 min 100 % eluent A, 24-25 min: gradient from 100- 10% eluent A, and 25-30 min: 10% of eluent A.

NMR-Z spectra were recorded on Varian Oxford AS 500 MHz.

The pH measurements were performed on a FiveEasy pH meter from Mettler Toledo

UV-vis spectra were recorded with an Agilent 8543 spectrophotometer. Raw data were processed using Agilent UV-vis Chemstation B.02.01 SP1, Spectragryph 1.2, OriginPro 8.5 and MS Excel.

For irradiation experiments outside of the spectrophotometer, the following lamps were used

For **1-Yb** and **1-Gd**: λ = 365 nm, spectroline ENB-280C/FE UV lamp.

For **3-Yb**: λ = 400 nm, 3 x Roithner VL-400 Emitter, 3 x 333 mW, FWHM 13 nm.

2. Synthetic procedures and spectroscopic data

**Figure S1.** Overview of the synthetic route towards compound **1-Gd** and **1-Yb**.

**Compound S1: 2-((4,5-dimethoxy-2-nitrobenzyl)(methyl)amino)ethan-1-ol:** Prepared by a modification of a literature procedure.^1^ A 50 mL round bottom flask under nitrogen was charged with 2-(methylamino)ethan-1-ol (0.58 mL, 7.3 mmol, 2.0 eq), Et_3_N (0.92 mL, 6.6 mmol, 1.8 eq) and anhydrous DCM (14 mL). The reaction mixture was while stirring placed on an ice bath. In a second 50 mL round bottom flask, under nitrogen, a solution of 1-(bromomethyl)-4,5-dimethoxy-2-nitrobenzene (1.02 g, 3.62 mmol, 1.0 eq) in dry DCM (6 mL) was prepared. This second solution was added gradually to the first solution and the reaction was stirred for one hour on ice bath. Upon completion, the solvent was evaporated at low pressure. The brown residue was then dissolved in DCM (30 mL) and was washed with water (3x20 mL) and once with brine (1x20 mL) followed by a drying step with sodium sulfate. After concentrating in vacuo, product **S1** was obtained as a brown oil in 73% (0.733 g, 2.71 mmol). ^1^H NMR (400 MHz, CDCl_3_) δ 7.54 (s, 1H, ArH), 7.23 (s, 1H, ArH), 3.98 (s, 6H, 2xCH_3_O), 3.93 (s, 2H, ArCH_2_), 3.69 (t, J = 5.2 Hz, 2H, CH_2_OH), 2.68 (s, 2H, CH_2_N), 2.34 (s, 3H, CH_3_N). ^13^C NMR (101 MHz, CDCl_3_) δ 152.9, 141.6, 132.6, 112.5, 108.15, 59.1, 58.6, 58.53, 56.6, 56.4, 42.1. IR data (cm-1): 3403 (O-H stretch), 3091 (aromatic C-H stretch), 2942, 2845 (alkyl C-H stretch), 1267 (alkyl aryl ether C-O stretch), 1216, 1062 (C-N amine). HRMS (ESI+) calc. for [M+H]^+^ (C_12_H_19_O_5_N_2_) 271.1288, found: 271.1289

**Compound 5: 2-bromo-*N*-(4,5-dimethoxy-2-nitrobenzyl)-*N*-methylethan-1-amine**: Prepared by a modification of a literature procedure.^2^ A solution of compound **S1** (0.73 g, 2.71 mmol, 1.0 eq) in anhydrous DCM (18 mL) was cooled down to 0 °C with an ice bath. Afterwards, NBS (0.622 g, 3.50 mmol, 1.30 eq) and TPP (0.85 g, 3.24 mmol, 1.20 eq) were added to the solution. The reaction mixture was stirred at 0°C for 10 minutes followed by 90 minutes at rt. The reaction was followed with TLC (DCM/acetone = 98:2). Upon completion, the reaction mixture was quenched with a saturated solution of NaHCO_3_ (0.8 g/10 mL) and the layers were separated. The aqueous layer was extracted with DCM (3x20 mL). The combined organic layers were washed once with a Na_2_S_2_O_3_ solution (0.5g/10 mL), once with brine (20 mL) and then dried over MgSO_4_. The filtrate was concentrated under reduced pressure. The crude residue was purified by recrystallization from ether (50 mL) affording the desired compound **5**. Analytical sample was purified via column chromatography (DCM/acetone=98:2) giving 10 mg of material for NMR analysis. Compound **5** was used in the next step without further purification. ^1^H NMR (400 MHz, CDCl_3_) δ 7.57 (s, 1H, ArH), 7.49 (s, 1H, ArH), 4.00 (s, 2H, ArCH_2_), 3.94 (s, 6H, 2xCH_3_O), 3.47 (t, *J* = 6.7 Hz, 2H, CH_2_Br), 2.86 (t, *J* = 6.6 Hz, 2H, NCH_2_), 2.32 (s, 3H, CH_3_N) ^13^C NMR (101 MHz, CDCl_3_) δ 153.3, 147.4, 141.1, 139.2, 130.4, 111.8, 107.9, 58.7, 58.4, 56.6, 56.3, 42.2, 30.5. IR data (cm-1): 2940, 2846 (alkyl C-H stretch), 1270 (alkyl aryl ether C-O stretch), 1186 (amine C-N stretch), 537 (C-Br stretch). HRMS (ESI+) calc. for [M+H]^+^ (C_12_H_18_O_4_N_2_Br) 335.0424, found: 335.0426.

**Compound 6: Tri-*tert*-butyl 2,2',2''-(1,4,7,10-tetraazacyclododecane-1,4,7-triyl)triacetate**: Prepared via a literature procedure.^3^ A round neck flask was charged with cyclen (1.00 g, 5.81 mmol, 1.00 eq.) and sodium acetate (1.57 g, 19.1 mmol, 3.30 eq.) and dimethylacetamide (DMA) (12 mL). The resulting solution was cooled down to -20 °C with an ice bath. Afterwards, a solution of *tert*-butyl bromoacetate (3.71 g, 19.1 mmol, 3.30 eq.) in DMA (4 mL) was added dropwise. The resulting white suspension was allowed to warm up to room temperature and was stirred overnight. Upon completion of the reaction (UPLC-MS), the reaction mixture was poured into water (65 mL), resulting in a colorless solution. To this solution potassium bicarbonate (3.00 g, 30.0 mmol) was added in portions. The formed precipitate was collected by filtration and dissolved in chloroform (50 mL). The solution was washed 3 times with water (25 mL) and dried with magnesium sulfate. The filtrate was concentrated under reduced pressure. The crude residue was purified by recrystallization with ether (50 mL) affording the desired pure compound **6** as a white solid. Isolated yield 64% (2.27 g, 3.80 mmol). Mp. 176-178 °C; ^1^H NMR (400 MHz, CDCl_3_) δ 10.18 (s, 2H), 3.32 (s, 4H), 3.24 (s, 2H), 3.04 (t, *J* = 4.8 Hz, 4H), 2.91 – 2.78 (m, 12H), 1.40 (d, *J* = 3.1 Hz, 27H) ppm.^13^C NMR (101 MHz, CDCl_3_) δ 170.5, 169.6, 81.7, 81.56, 58.2, 51.3, 49.2, 47.5, 28.2, 28.1 ppm. NMR spectra are in agreement with the literature data.^3^ HRMS (ESI+) calc. for [M+H]^+^ (C_26_H_51_N_4_O_6_): 515.3803, found: 515.3801.

**Compound 7: tri-*tert*-butyl 2,2',2''-(10-(2-((4,5-dimethoxy-2-nitrobenzyl)(methyl)-amino)ethyl)-1,4,7,10-tetraazacyclododecane-1,4,7-triyl)triacetate:** Prepared via a modification of a published procedure for another product.^4^ A two necked flask was placed under a nitrogen atmosphere and charged with a solution of compound **6** (161 mg, 0.27 mmol, 1.0 eq.) in 15 mL ACN. Additionally, Cs_2_CO_3_ (527 mg, 1.62 mmol, 6.0 eq) and compound **5** (360 mg, 1.08 mmol, 4.0 eq) were added. The reaction mixture was stirred at room temperature under N_2_ for 6 h. The reaction was followed by UPLC-MS, upon completion, the reaction mixture was filtered, and the solvent was evaporated to obtain crude **7** as yellowish oil. Compound **7** was used in the next step without further purification.

**Compound 1: 2,2',2''-(10-(2-((4,5-dimethoxy-2-nitrobenzyl)(methyl)amino)ethyl)-1,4,7,10-tetraazacyclodode-cane-1,4,7-triyl)triacetic acid:** To a solution of compound **7** (500 mg, 0.65 mmol, 1.0 eq.) in 6 mL of DCM, tri-iso-propylsilane (TIPS) (0.90 mL, 4.40 mmol, 6.77 eq.) and TFA (6 mL) was added. The reaction mixture was stirred at rt overnight. Upon completion (checked by UPLC-MS) the product reaction mixture was concentrated at low pressure. The precipitated solid was washed with ether (3 x 15 mL), DCM (3 x 15 mL) and pentane (3 x 15 mL). Crude Product **1** was obtained as an orange solid And was used in the next step without further purification. ^1^H NMR (400 MHz, Deuterium Oxide) δ 7.81 (s, 1H), 7.13 (s, 1H), 3.99 – 3.77 (m, 16H), 3.38 (s, 15H), 3.20 – 2.65 (m, 17H), 2.08 (s, 1H). ESI- HRMS (ESI+) calc. for [M+H]^+^ (C_26_H_43_N_6_O_10_): 599.3035, found: 599.3017

**Compound 1-Yb**: Compound **1** (185 mg, 0.31 mmol 1 eq) was dissolved in water (5 mL), afterwards a solution of YbCl_3_ (108 mg, 0.27 mmol, 0.90 eq) in water (4 mL) was added. The pH was adjusted to pH 6.5 with 1 M LiOH and the level of complexation was checked with UPLC-MS. The reaction mixture was purified by preparative HPLC, using program 4 (see general information section 1). The product (Rt = 12.1 min) was collected and freeze dried affording the desired compound **1-Yb** as a fluffy yellow solid. Isolated yield 36% (50 mg, 0.06 mmol). For ^1^H-NMR see Figure S25. HRMS (ESI+) calc. for [M+H]^+^ (C_26_H_40_N_6_O_10_Yb): 770.2189, found: 770.2169.

**Compound 1-Gd:** Compound **1** (50 mg, 0.08 mmol 1.0 eq) was dissolved in water (5 mL), afterwards a solution of YbCl_3_ (108 mg, 0.27 mmol, 0.90 eq) in water (4 mL) was added. The pH was adjusted to pH 6.5 with 1 M LiOH and the level of complexation was checked with UPLC-MS. The reaction mixture was purified by preparative HPLC, using program 4 (see general information section 1). The product (Rt = 12.4 min) was collected and freeze dried affording the desired compound **1-Gd** as a fluffy yellow solid. Isolated yield 45% (17 mg, 0.022 mmol). For 1H-NMR see Figure S26. HRMS (ESI+) calc. for [M+H]^+^ (C_26_H_40_N_6_O_10_Gd): 754.2049, found: 754.2041

**Figure S2.** Synthetic overview of the route towards compound **3-Gd** and **3-Yb**.

**Compound 8: 7-(diethylamino)-4-(2-hydroxypropan-2-yl)-2H-chromen-2-one:** To a solution of 4-acetyl-7-(diethylamino)-2H-chromen-2-one prepared according to literature procedure^6^ (878 mg, 3.38 mmol, 1.00 eq.) in dry THF (27 mL) under nitrogen atmosphere at -78 °C was slowly added methyl magnesium bromide in Et_2_O (3M, 1.25 mL, 1.10 eq.). The reaction mixture was stirred for 15 min at -78 °C, allowed to warm to room temperature and stirred for another 2 h. Subsequently, sat. aq. NH_4_Cl was added, and the aquas layer was extracted with EtOAc (3x). The combined organic layers were washed with brine , dried with MgSO_4_ and concentrated under reduced pressure. The crude material was purified by silica gel chromatography (DCM/acetone 98:2 to 95:5) to yield compound **8** as a dark orange solid (584 mg, 2.1 mmol, 62 %). ^1^H-NMR (400 MHz, CDCl_3_) δ 8.08 (d, *J* = 9.3 Hz, 1H, ArH), 6.55 (dd, *J* = 9.3, 2.7 Hz, 1H, ArH), 6.46 (d, *J* = 2.7 Hz, 1H, ArH), 6.12 (s, 1H, C=CH), 3.39 (q, *J* = 7.1 Hz, 4H, 2x NCH_2_), 2.44 (s, 1H, OH), 1.67 (s, 6H, 2x HOCC**H_3_**), 1.19 (t, *J* = 7.1 Hz, 6H, 2 x CH_2_C**H_3_**). ^13^C-NMR (101 MHz, CDCl_3_) δ 162.9, 161.3, 157.1, 145.0,6 129.0, 108.3, 106.4, 105.7, 98.1, 73.0, 44.7, 30.4, 12.6. HRMS (ESI+): calc. for [M+H^+^] (C_16_H_22_NO_3_) :276.1594; found: 276.1593. mp. 106.5-107.0 °C.

**Compound 9: Tri-tert-butyl 2,2',2''-(10-(2-aminoethyl)-1,4,7,10-tetraazacyclododecane-1,4,7-triyl)triacetate:** Prepared via a literature procedure.^4^ A three necked flask was placed under nitrogen atmosphere and charged with a solution of compound **6** (653 mg, 1.10 mmol, 1.00 eq.) in MgSO_4_ dried ACN (10 mL). Afterwards, *N*-(2-Bromoethyl)phthalimide (653 mg, 1.1 mmol, 1.0 eq.) and cesium carbonate (720 mg, 2.20 mmol, 2.00 eq.) were added. The resulting yellow suspension was heated to 80 °C and stirred for 24 h. Upon completion (UPLC-MS), the crude reaction mixture was filtered. The yellow filtrate was evaporated under reduced pressure and chloroform (20 mL) was added. The impurities were filtered off and the solvent was evaporated, affording the desired crude compound **11** as a yellow oil. Rf = 0.65 (DCM/MeOH, 9:1, v/v); ^1^H-NMR (400 MHz, CDCl_3_): δ 7.82-7.84 (m, 2H), 7.68–7.71 (m, 2H), 3.74-3.77 (t, *J*=6.7Hz, 2H), 3.21 (s, 6H), 2.69-2.79 (m, 18H), 1.44 (s, 27H) ppm. Compound **11** was used in the next step without further purification. The crude compound **11** was dissolved in methanol (14 mL) and hydrazine hydrate (reagent grade 50-60%, 120 µL) was added while stirring. The reaction mixture was heated to 70 °C and stirred for 4 h. Upon reaction completion (UPLC-MS), the solvent was evaporated. DCM (20 mL) was added, and the resulting solids were filtered out. The filtrate was washed first with distilled water (three times, 30 mL) and then with an aqueous solution of KOH (20%, 20 mL). The organic layer was dried with magnesium sulfate. Evaporation of the solvent under reduced pressure yielded compound **9** as an amber oil (465 mg, 88% over two steps from compound **6**). Rf = 0.64 (DCM/MeOH, 9:1, v/v) ^1^H NMR (400 MHz, Chloroform-d) δ 3.45 – 3.24 (m, 6H), 3.13 – 2.33 (m, 20H), 1.42 (s, 27H).^13^C NMR (101 MHz, Chloroform-d) δ 171.5, 170.6, 81.6, 81.1, 56.5, 56.1, 51.6, 50.5, 50.0, 49.6, 46.2, 37.5, 28.2, 28.1. NMR spectra in agreement with the literature data.^4^ HRMS (ESI+) calc. for [M+H]^+^ (C_28_H_56_N_5_O_6_^+^): 558.4225, found: 558.4222.

**Compound 3: tri-*tert*-butyl 2,2',2''-(10-(2-((((2-(7-(diethylamino)-2-oxo-2H-chromen-4-yl)propan-2-yl)oxy)carbonyl)amino)ethyl)-1,4,7,10-tetraazacyclododecane-1,4,7-triyl)triacetate:** A three necked flask was put under nitrogen atmosphere and charged with molecular sieves (3 Å beads, 4-8 mesh). A solution of compound **8** (15 mg, 0.055 mmol, 1.00 eq.) and 4-dimethylaminopyridine (DMAP) (10.1 mg, 0.0825 mmol, 1.50 eq.) in dry DCM (2 mL) was added. While stirring, diphosgene (6 μL, 0.038 mmol, 0.9 eq.) was added and the reaction mixture was stirred and followed by NMR. Upon 80% conversion (1h), a solution of compound **9** (30 mg, 0.053 mmol, 1.00 eq.) in dry DCM (1 mL) was added, and the reaction mixture was followed by UPLC-MS. After completion, the reaction mixture was transferred to a 20 mL glass vial, and the molecular sieves were removed, subsequently the solvent was evaporated to obtain crude compound **10** as dark amber oil. To the 20 mL vail containing compound **10** was added 0.4 mL TFA and TIPS (0.011 mL, 0.055 mmol, 1.0 eq.). The resulting slightly yellow reaction mixture was left stirring at rt overnight. Upon complete deprotection (UPLC-MS), 15 mL of ether was added, and the solvent was decanted off, this was repeated 2 more times. Afterwards the solid was washed with pentane (3x15 mL), DCM (3x15 mL) and ethyl acetate (3x 15 mL). The compound was dried in vacuo affording the crude compound **3** as an eggshell white powder.

The reaction was once purified via prep-HPLC with method 6 (see general information section 1) giving 8.5 mg (22% yield) of material for ^1^H analysis. Compound **3** was used in the next step without further purification. ^1^H NMR (400 MHz, Deuterium Oxide) δ 7.98 (d, *J* = 9.3 Hz, 1H), 6.82 (dd, *J* = 9.5, 2.6 Hz, 1H), 6.70 (d, *J* = 2.6 Hz, 1H), 6.12 (s, 1H), 3.79 – 2.52 (m, 31H), 1.74 (s, 6H), 1.13 (t, *J* = 7.0 Hz, 6H). HRMS (ESI+) calc. for [M+H]^+^ (C_33_H_51_N_6_O_10_):691.3661, found: 691.3655.

**Compound 3-Yb:** A 20 mL flask was charged with all the crude of compound **3** and water was added (2 mL). Ytterbium chloride (12 mg, 0.042 mmol, 0.80 eq) was added to the solution. Afterwards, the mixture was stirred, and the pH was adjusted to 6.0 with 1 M LiOH (very slowly 10 μL at a time on a total of 110 μL) After reaching pH 6 the complexation was confirmed by UPLC-MS, after conformation the reaction mixture was purified by preparative HPLC, using program 5 (see general information section 1). The product (Rt = 13.5 min) was collected and freeze dried affording the desired compound **3-Yb** as a pale yellow fluffy solid. Isolated yield 11% (5.1 mg, 0.006 mmol). For ^1^H-NMR see Figure S29. HRMS (ESI+) calc. for [M+H]^+^ (C_33_H_46_YbN_6_O_10_^+^):862.2826, found: 862.2815.

**Compound 3-Gd:** A 20 mL flask was charged with all the crude of compound **3** and water was added (2 mL). Gadolinium chloride (11 mg, 0.042 mmol, 0.80 eq) was added to the solution. Afterwards, the mixture was stirred, and the pH was adjusted to 6.0 with 1 M LiOH (very slowly 10 μL at a time on a total of 120 μL) After reaching pH 6 the complexation was confirmed by UPLC-MS, after conformation the reaction mixture was purified by preparative HPLC, using program 5 (see general information section 1). The product (Rt = 13.4 min) was collected and freeze-dried affording impure compound **3-Gd** as a fluffy slightly yellow fluffy solid. See also UPLC-MS Figure S55.

**Compound 4**: 2,2',2''-(10-(2-ammonioethyl)-1,4,7,10-tetraazacyclododecane-1,4,7-triyl)triacetate: Compound **9** (260 mg, 0.47 mmol, 1.00 eq) was dissolved in 10 mL DCM, and tri-iso-propylsilane (TIPS) (0.96 mL, 4.49 mmol, 10 eq.) was added. The reaction mixture was stirred and 2 M HCl in ether (10 mL) was added. The resulting slightly white reaction mixture was stirred overnight at rt. Upon complete deprotection (UPLC-MS), the solid was filtered off and washed with 3x 30 mL DCM, 3x 30 mL ether, 3x 30 mL ethyl acetate and 3x 30 mL pentane, affording the compound **4** as slightly yellow powder. Isolated yield 90% (163 mg, 0.42 mmol). ^1^H NMR (400 MHz, Deuterium Oxide) δ 4.30 – 3.95 (m, 4H), 3.91 – 3.29 (m, 11H), 3.26 – 2.76 (m, 11H) ppm. ^13^C NMR (151 MHz, Deuterium Oxide) δ 174.4, 168.5, 90.4, 85.8, 54.9, 53.0, 51.9, 50.5, 49.9, 48.5, 36.0 ppm. NMR spectra in agreement with the literature data.^7^ HRMS (ESI+) calc. for [M+H]+ (C_16_H_32_N_5_O_6_^+^): 390.2347, found: 390.2345.

**Compound 4-Yb:** A 20 mL vial was charged with compound **4** (100 mg, 0.256 mmol, 1.0 eq) dissolved in water (6 mL). Ytterbium chloride (79.4 mg, 0.205 mmol, 0.80 eq) was added, the vial was shaken, and the pH was adjusted to pH 6.0 with 1 M LiOH. Afterwards the reaction mixture was stirred at 60 °C for 24 h, the pH was adjusted to pH 6.5 and the complexation was checked with LCMS. The crude complex was freeze dried affording the desired compound **4-Yb** as a slightly yellow solid. Isolated yield 95 % (136.0 mg, 0.242 mmol). For ^1^H-NMR see Figure S43. HRMS (ESI+) calc. for [M+H]+ (C_16_H_29_N_5_O_6_Yb^+^): 561.1500, found:561.1505.

3. NMRD profiles

3.1. NMRD profile of 1-Gd

NMRDs were recorded on a Stelar 0.25T FFC SMARtracer relaxometer (Mede, Italy). The relaxation rates were determined over a (proton) Larmor frequency range of 0.01–10 MHz at 37 °C with 17 data points collected.

A stock solution of **1-Gd** was prepared (1 mM in 3 mL Milli Q, pH 7.4) and divided into two samples: blank and the uncaging reaction with 365 nm light. NMRD profiles were recorded before and after irradiation with light (10 sec, 20 sec, 30 sec, 60 sec, 240 sec, 6 min (Table S1), 12 min, 24 min,48 min, 96 min and 192 min (Table S3)). In addition, the stability of the blank was assessed by repeating the analysis after leaving the sample for 72 h at 37 °C without light. Samples were taken to analyses compounds present via UPLC-MS. (Figure S3-5)

The pH was measured after the irradiation of the samples, see table S5.

The relaxation rates of **1-Gd**, at 4.7T were recorded with inversion recovery method at 37 °C. The solutions of the time point t0 and t192 were then transferred to a 3 mm sample tubes (0.25 mL per tube) and measured. Every data point measured in duplicate (table S7).

Table S1. Molar relaxivity (s^-1^ mM^-1^) profiles of a sample of **1-Gd** at pH 7.4. Irradiation times 0-6 min. The uncertainty of the measurement is shown in Table S2.

| MHz | t0 | t1 10 sec | t2 30 sec | t3 60 sec | t4 2 min | t5 6 min |
| --- | --- | --- | --- | --- | --- | --- |
| 10.00 | 4.8762 | 4.8361 | 4.8174 | 4.7996 | 4.7989 | 4.2372 |
| 6.31 | 5.1106 | 5.1788 | 5.1104 | 4.9184 | 4.9479 | 4.5878 |
| 3.98 | 5.2502 | 5.3213 | 5.4542 | 5.2642 | 5.2579 | 4.6365 |
| 2.51 | 5.4640 | 5.7012 | 5.5578 | 5.5813 | 5.6436 | 4.8412 |
| 2.51 | 5.6125 | 5.5294 | 5.6336 | 5.5233 | 5.6036 | 4.9101 |
| 1.59 | 5.9445 | 5.9342 | 5.7385 | 5.6716 | 5.3892 | 5.0695 |
| 1.00 | 5.9756 | 6.1152 | 5.8619 | 5.8947 | 5.8080 | 5.3801 |
| 0.63 | 5.9618 | 6.0029 | 5.9416 | 6.0283 | 5.7170 | 5.2488 |
| 0.40 | 6.0728 | 5.9822 | 5.8134 | 5.8129 | 5.6726 | 5.4672 |
| 0.25 | 6.0774 | 6.0911 | 5.9905 | 5.8321 | 5.8339 | 5.3603 |
| 0.16 | 6.0728 | 5.9350 | 5.8177 | 5.8532 | 5.7841 | 5.3727 |
| 0.10 | 6.1455 | 6.0166 | 6.0022 | 5.8312 | 5.8763 | 5.3650 |
| 0.06 | 6.1484 | 6.0079 | 6.1658 | 6.0367 | 5.8233 | 5.5225 |
| 0.04 | 6.1094 | 6.1070 | 6.0291 | 5.8412 | 5.7734 | 5.2095 |
| 0.03 | 6.0013 | 6.1732 | 5.9676 | 5.7338 | 5.7824 | 5.3464 |
| 0.02 | 5.9969 | 5.7418 | 5.9046 | 5.8012 | 5.9039 | 5.3529 |
| 0.01 | 6.0612 | 5.8616 | 5.9627 | 5.7439 | 5.8326 | 5.3943 |

Table S2. The error bars represent the uncertainty of fitting the T_1_ curve to the experimental data of irradiation times 0-6 min (For data presented in table S1).

| t0 | t1 10 sec | t2 30 sec | t3 60 sec | t4 2 min | t5 6 min |
| --- | --- | --- | --- | --- | --- |
| 4.25E-02 | 2.96E-02 | 3.24E-02 | 2.56E-02 | 3.40E-02 | 2.78E-02 |
| 5.69E-02 | 4.67E-02 | 4.44E-02 | 4.27E-02 | 4.93E-02 | 4.92E-02 |
| 7.11E-02 | 7.51E-02 | 8.02E-02 | 8.51E-02 | 7.69E-02 | 5.37E-02 |
| 7.21E-02 | 8.89E-02 | 8.44E-02 | 7.61E-02 | 7.42E-02 | 6.81E-02 |
| 9.05E-02 | 1.04E-01 | 6.99E-02 | 7.02E-02 | 7.39E-02 | 7.78E-02 |
| 5.74E-02 | 6.68E-02 | 5.05E-02 | 4.52E-02 | 6.79E-02 | 7.94E-02 |
| 8.84E-02 | 6.29E-02 | 6.22E-02 | 5.89E-02 | 7.82E-02 | 4.82E-02 |
| 4.69E-02 | 5.39E-02 | 6.44E-02 | 6.52E-02 | 6.26E-02 | 4.79E-02 |
| 6.17E-02 | 4.65E-02 | 4.79E-02 | 6.77E-02 | 5.86E-02 | 4.77E-02 |
| 5.25E-02 | 6.20E-02 | 5.59E-02 | 5.74E-02 | 4.38E-02 | 5.41E-02 |
| 5.87E-02 | 6.02E-02 | 7.24E-02 | 3.80E-02 | 6.20E-02 | 5.04E-02 |
| 6.31E-02 | 5.96E-02 | 6.64E-02 | 5.28E-02 | 5.64E-02 | 4.10E-02 |
| 8.12E-02 | 6.57E-02 | 7.83E-02 | 5.72E-02 | 6.75E-02 | 7.27E-02 |
| 7.92E-02 | 8.78E-02 | 5.07E-02 | 7.23E-02 | 5.36E-02 | 5.92E-02 |
| 8.35E-02 | 7.07E-02 | 6.26E-02 | 7.17E-02 | 5.09E-02 | 6.74E-02 |
| 6.17E-02 | 6.77E-02 | 7.18E-02 | 6.79E-02 | 8.17E-02 | 7.32E-02 |
| 7.41E-02 | 8.36E-02 | 6.83E-02 | 5.92E-02 | 5.43E-02 | 7.27E-02 |

Table S3. Molar relaxivity (s^-1^ mM^-1^) profiles of a sample of **1-Gd** at pH 7.4. Irradiation times 12- 192 min. The uncertainty of the measurement is shown in Table S4.

| MHz | t6 12 min | t7 24 min | t8 48 min | t9 96 min | t10 192 min | t0 after 3 days in the dark |
| --- | --- | --- | --- | --- | --- | --- |
| 10.00 | 3.9592 | 3.8133 | 3.7279 | 3.6390 | 3.6635 | 4.9927 |
| 6.31 | 4.1563 | 3.9968 | 4.0336 | 3.9367 | 3.9956 | 5.1997 |
| 3.98 | 4.3601 | 4.1270 | 4.3348 | 4.2959 | 4.0708 | 5.7170 |
| 2.51 | 4.6668 | 4.7165 | 4.2863 | 4.5345 | 4.4048 | 5.7793 |
| 2.51 | 4.7269 | 4.3721 | 4.3461 | 4.4085 | 4.4085 | 5.7277 |
| 1.59 | 4.9974 | 4.6052 | 4.5696 | 4.4316 | 4.8359 | 6.0338 |
| 1.00 | 5.1595 | 4.9068 | 4.5994 | 4.7404 | 4.6588 | 6.0673 |
| 0.63 | 5.0484 | 4.7738 | 4.7940 | 4.4680 | 4.6349 | 6.0339 |
| 0.40 | 4.8860 | 4.8458 | 4.6990 | 4.4490 | 4.7868 | 6.2362 |
| 0.25 | 5.0245 | 4.8103 | 4.7896 | 4.6736 | 4.8439 | 6.0771 |
| 0.16 | 4.9717 | 4.7382 | 4.7403 | 4.7491 | 4.8224 | 6.3454 |
| 0.10 | 5.0188 | 4.7910 | 4.6521 | 4.7087 | 4.7453 | 6.2260 |
| 0.06 | 5.0245 | 4.8681 | 4.8418 | 4.5955 | 4.6843 | 5.9529 |
| 0.04 | 5.0234 | 4.7274 | 4.6693 | 4.6725 | 4.7982 | 6.2641 |
| 0.03 | 4.9654 | 4.7600 | 4.8244 | 4.7528 | 4.7949 | 6.1595 |
| 0.02 | 5.1161 | 4.8080 | 4.9542 | 4.7914 | 4.8845 | 6.2122 |
| 0.01 | 5.0524 | 4.8416 | 4.6697 | 4.7938 | 4.6546 | 6.0565 |

Table S4. The uncertainty of fitting the T_1_ curve to the experimental data of irradiation times 12- 192 min. (For data presented in table S3)

| t6 12 min | t7 24 min | t8 48 min | t9 96 min | t10 192 min | t0 after 3 days in the dark |
| --- | --- | --- | --- | --- | --- |
| 3.59E-02 | 2.80E-02 | 3.03E-02 | 2.80E-02 | 3.53E-02 | 4.22E-02 |
| 4.69E-02 | 3.89E-02 | 5.88E-02 | 5.45E-02 | 7.07E-02 | 4.34E-02 |
| 8.21E-02 | 6.46E-02 | 8.84E-02 | 9.16E-02 | 9.89E-02 | 9.76E-02 |
| 6.84E-02 | 6.88E-02 | 9.32E-02 | 8.54E-02 | 1.15E-01 | 8.53E-02 |
| 7.08E-02 | 7.30E-02 | 7.46E-02 | 6.47E-02 | 1.31E-01 | 8.14E-02 |
| 6.33E-02 | 6.75E-02 | 7.98E-02 | 8.91E-02 | 8.06E-02 | 7.25E-02 |
| 5.67E-02 | 5.89E-02 | 7.33E-02 | 5.81E-02 | 8.92E-02 | 8.11E-02 |
| 5.61E-02 | 5.68E-02 | 6.59E-02 | 5.79E-02 | 9.20E-02 | 5.64E-02 |
| 3.77E-02 | 4.71E-02 | 6.69E-02 | 4.74E-02 | 7.24E-02 | 4.79E-02 |
| 6.25E-02 | 5.85E-02 | 5.78E-02 | 6.04E-02 | 9.26E-02 | 5.34E-02 |
| 4.07E-02 | 3.92E-02 | 5.68E-02 | 4.05E-02 | 6.15E-02 | 5.58E-02 |
| 4.90E-02 | 4.93E-02 | 6.19E-02 | 5.68E-02 | 8.60E-02 | 6.58E-02 |
| 5.93E-02 | 4.66E-02 | 6.25E-02 | 8.10E-02 | 7.71E-02 | 7.60E-02 |
| 5.21E-02 | 6.47E-02 | 6.92E-02 | 6.42E-02 | 1.01E-01 | 7.18E-02 |
| 8.35E-02 | 6.35E-02 | 8.08E-02 | 7.36E-02 | 9.91E-02 | 7.92E-02 |
| 8.80E-02 | 5.35E-02 | 7.56E-02 | 6.93E-02 | 1.02E-01 | 8.91E-02 |
| 6.91E-02 | 6.55E-02 | 6.36E-02 | 1.04E-01 | 7.49E-02 | 1.07E-01 |

Table S5. The pH during the Molar relaxivity (s^-1^ mM^-1^) profiles of a sample of **1-Gd** over time (For data presented in table S1 and table S3)

| measurement | Measured pH | | |
| --- | --- | --- | --- |
| t=0 | | 7.40 | |
| t=1 (10 sec) | | 7.40 | |
| t=2 (30 sec) | | 7.40 | |
| t=3 (60 sec) | | 7.40 | |
| t=4 (120 sec) | | 7.40 | |
| t=5 (6 min) | | 7.40 | |
| t=6 (12 min) | | 7.39 | |
| t=7 (24 min) | | 7.37 | |
| t=8 (48 min) | | 7.37 | |
| t=9 (96 min) | | 7.37 | |
| t=10 (192 min) | | 7.37 | |
| t=0 after 3 days in the dark | | 7.39 |  |

Table S6. Molar relaxivity (s^-1^ mM^-1^) profile of a sample of **1-Gd** at pH 7.4 measured at 10 MHz * after 3 days in de dark.

| Min of irradation | Molar relaxivity (s^-1^ mM^-1^) |
| --- | --- |
| 0 | 4.8762 |
| 0* | 4.9927 |
| 0.1 | 4.8361 |
| 0.5 | 4.8174 |
| 1 | 4.7996 |
| 2 | 4.7989 |
| 6 | 4.2372 |
| 12 | 3.9592 |
| 24 | 3.8133 |
| 48 | 3.7279 |
| 96 | 3.6390 |
| 192 | 3.6635 |

Table S7. Molar relaxivity (s^-1^ mM^-1^) profiles of a sample of **1-Gd** at pH 7.4 measured at 4.7 T

| Measurement | t 0 min | t 192 min |  |
| --- | --- | --- | --- |
| 1 | 3,9575 | 1.9347 |  |
| 2 | 3.8531 | 1.9594 |  |
| average | 3.9053 | 1.9471 |  |


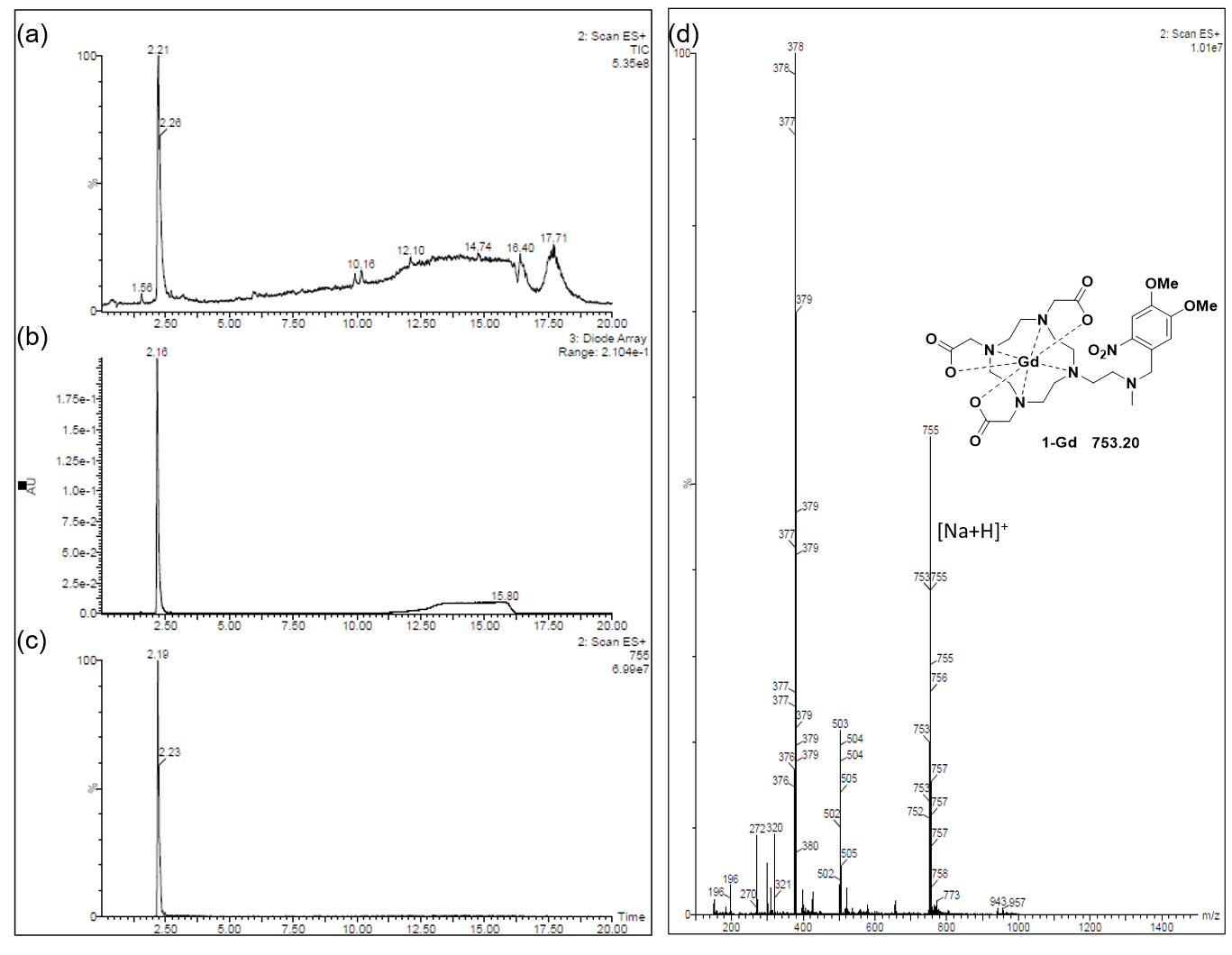


**Figure S3.** UPLC-MS analysis of **1-Gd** (the blank t=0), using UPLC-MS program 3 (see section 1), a) total ion current chromatogram (positive mode), b) UV-Vis at 365 nm chromatogram, c) 754-755 mass trace, d) mass spectrum of the peak at Rt = 2.19 min.


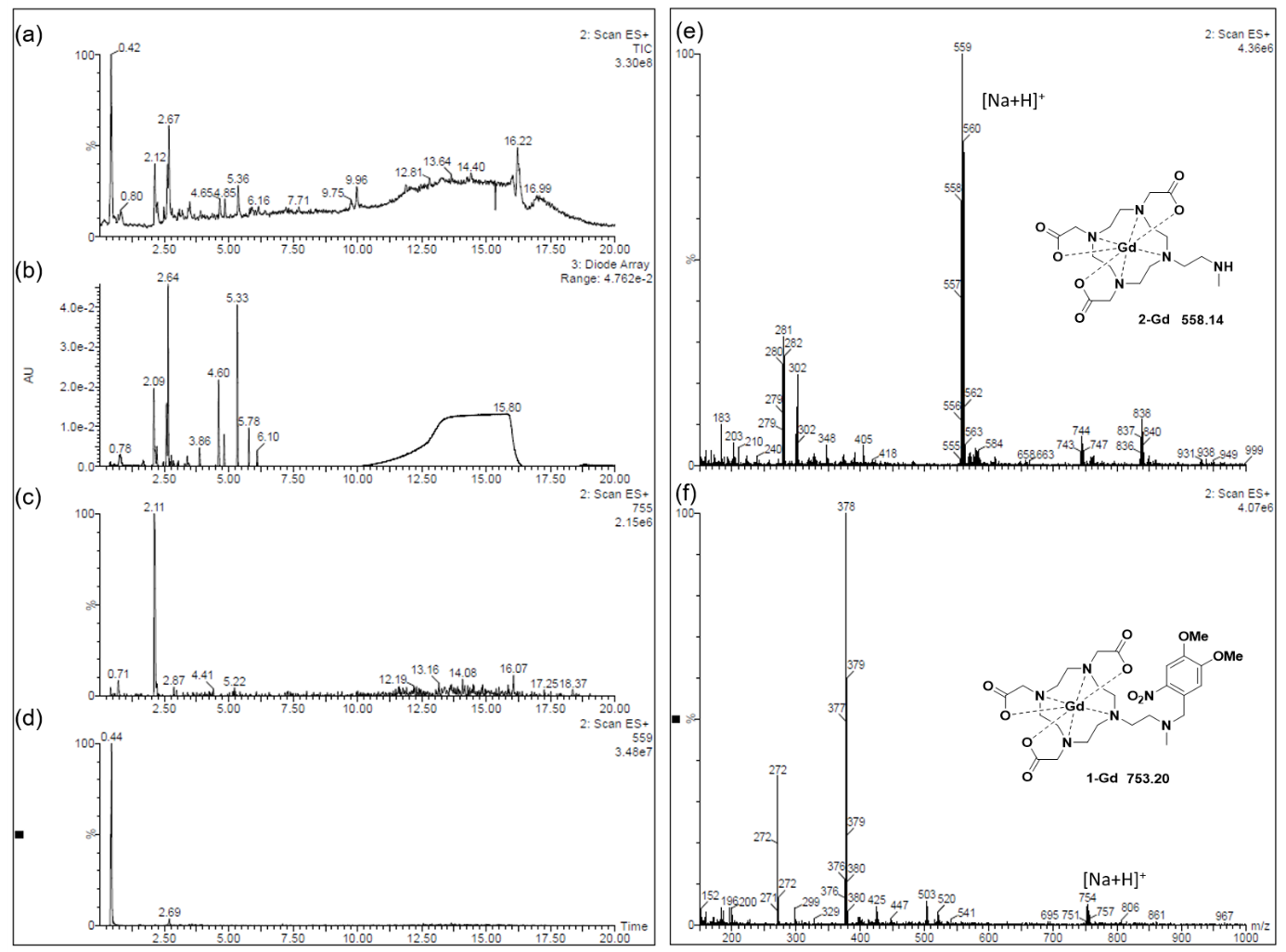


**Figure S4.** UPLC-MS analysis of **1-Gd** (t=192), using UPLC-MS program 3 (see section 1), a) total ion current chromatogram (positive mode), b) UV-vis at 365 nm chromatogram, c) 755 mass trace, d) 559 mass trace, e) mass spectrum of the peak at Rt = 0.44 min, f) mass spectrum of the peak at Rt = 2.11 min.


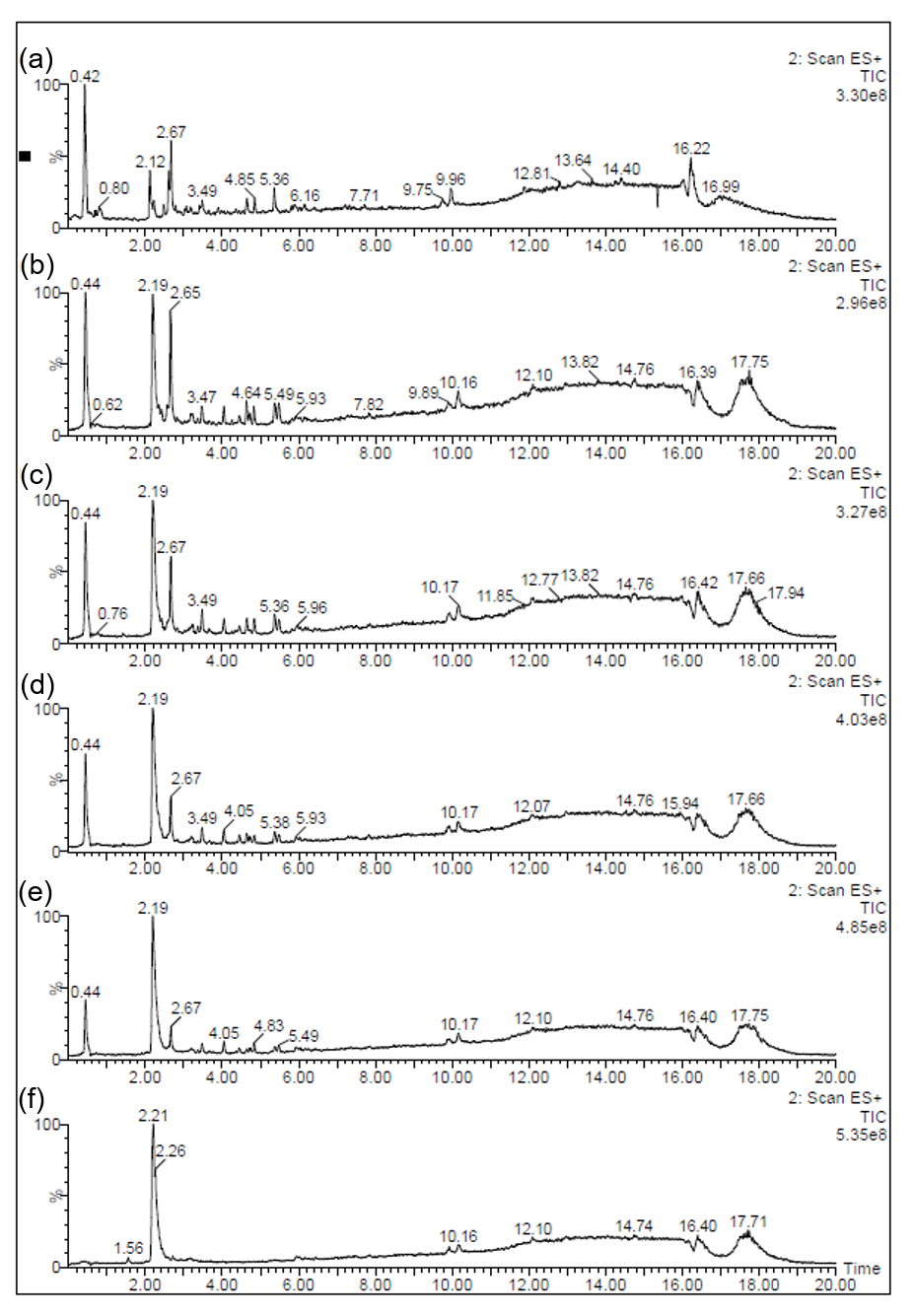


**Figure S5**. Overview of UPLC-MS analysis of **1-Gd** over irradiation time points. a) UPLC-MS TIC of t=192 min, b) UPLC-MS TIC of t=48 min, c) UPLC-MS TIC of t=24 min, (d) UPLC-MS TIC of t=12 min, e) UPLC-MS TIC of t=6 min and f) UPLC-MS TIC of t=0 min.

4. Z-spectra

All Z-spectra were recorded on a Varian Oxford AS 500 MHz (B0 = 11.7T), using 5 mm sample tubes. Stock solutions of **1-Yb** and **3-Yb** were prepared (see figure captions for exact concentrations) this stock solution was then divided into two samples, blank and the light uncaging reaction (0.6 mL each). The Z-spectra were recorded before and after irradiation with light (for exact wavelength see figure captions). In addition, the stability of the blank was assessed by repeating the analysis after leaving the sample for the same amount of time without the light in the solution. And all samples were analyzed by UPLC-MS at t=0 and t=last irradiation point (see Figures S8-9 and S12-13)

4.1. CEST of 1-Yb

**Figure S6.** Z-spectra of **1-Yb** (50 mM in water with 10% D_2_O, pH 7.4, B0 = 11.7 T, satpwr = 28 dB, satdly = 2 s) at T = 37 °C, time points: 0 min, 30 min, 60 min, 120 min and 180 min. Irradiation with 365 nm light.

**Figure S7**. Z-spectra of **1-Yb** (50 mM in water with 10% D_2_O, pH 7.4, B0 = 11.7 T, satpwr = 28 dB, satdly = 2 s) at T = 37 °C, time points: t=0 and t= after 180 min in the dark.


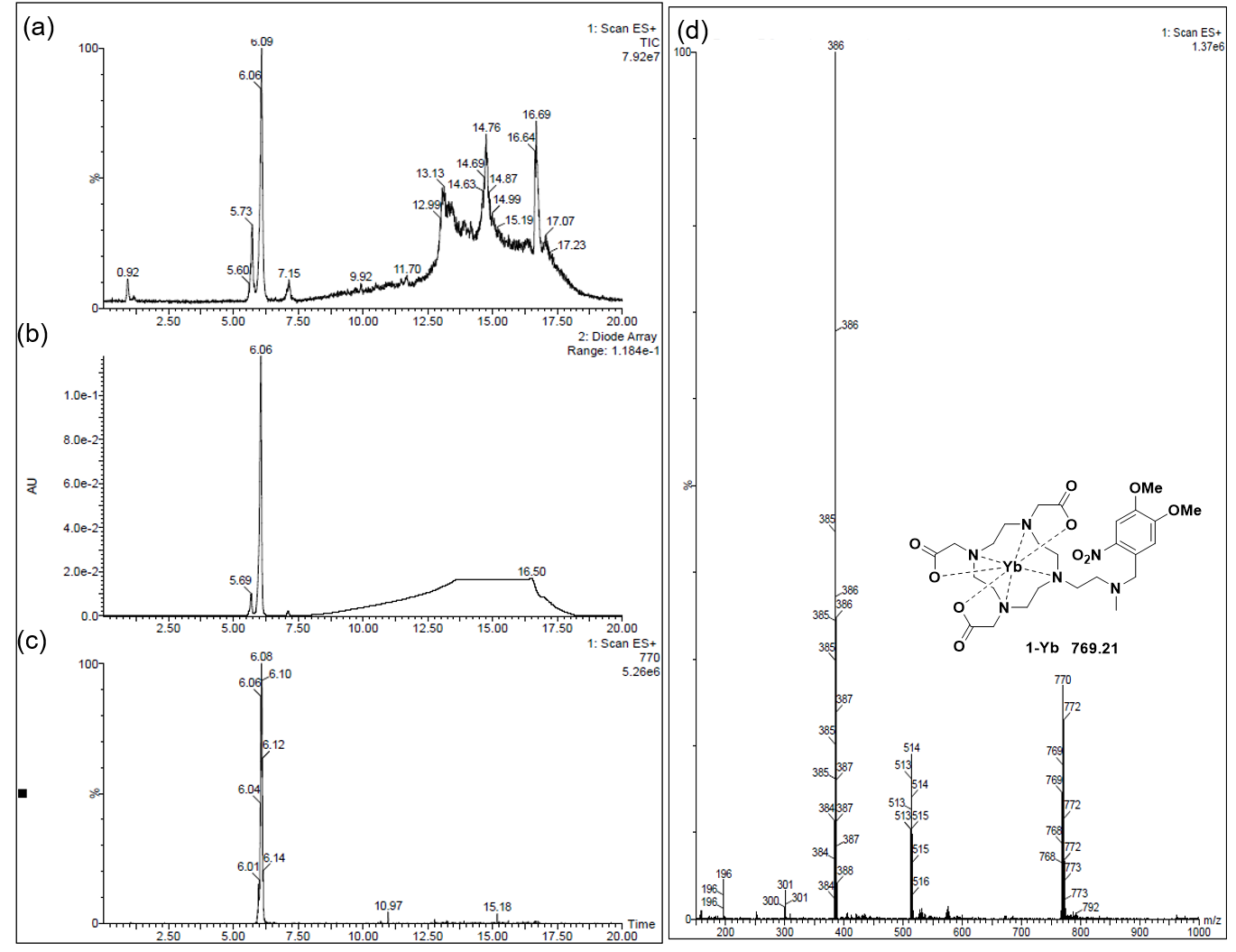


**Figure S8.** UPLC-MS analysis of **1-Yb** (sample t= 0 min) using UPLC-MS program 3 (see section 1), a) total ion current chromatogram (positive mode), b) UV-vis at 365 nm chromatogram, c) 769-770 mass trace, d) mass spectrum of the peak at Rt = 6.08 min.


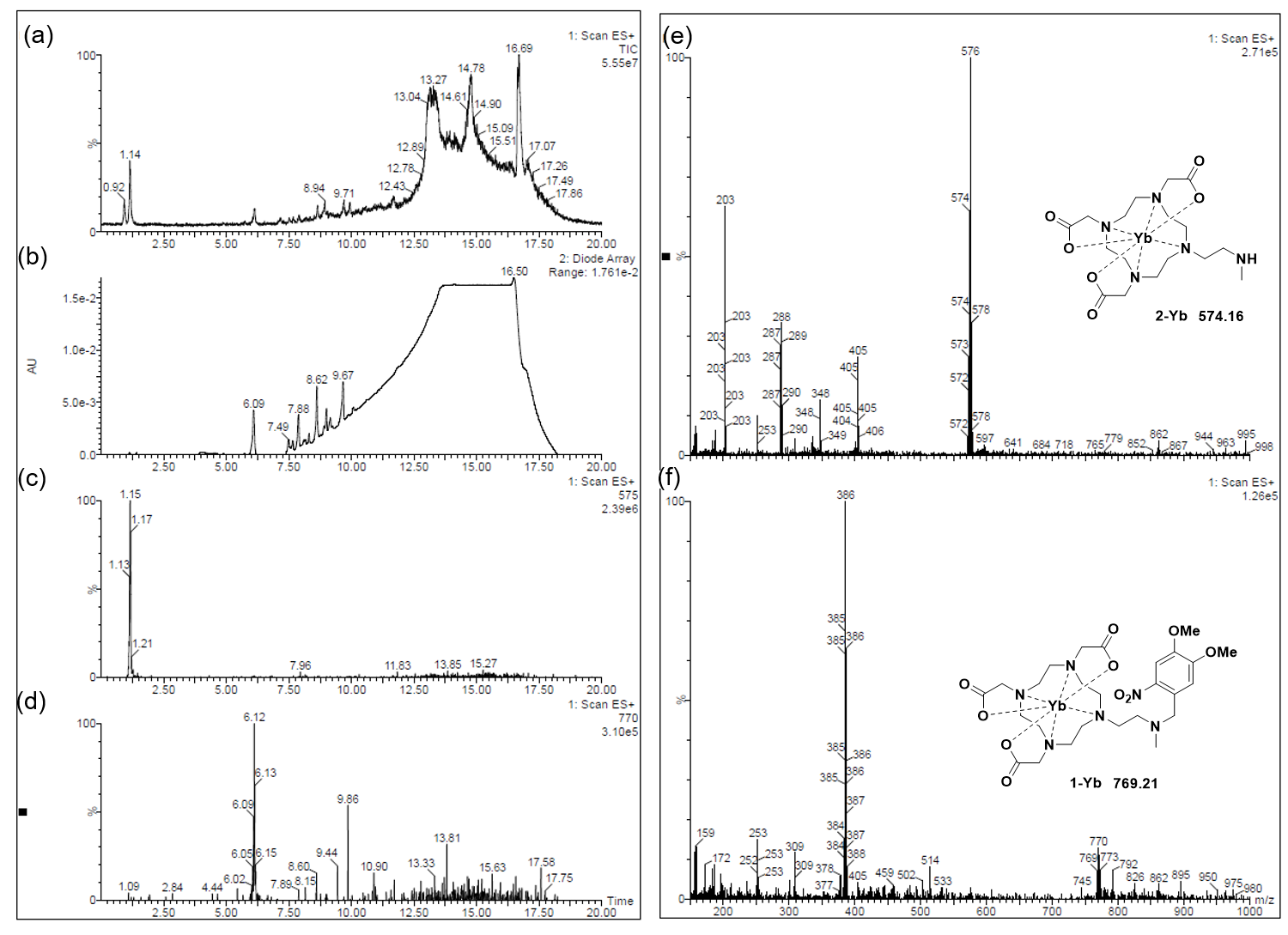


**Figure S9.** UPLC-MS analysis of **1-Yb** (t= 180 min) using UPLC-MS program 3 (see section 1), a) total ion current chromatogram (positive mode), b) UV-vis at 365 nm chromatogram, c) 574-575 mass trace, d) 769-770 mass trace, e) mass spectrum of the peak at Rt = 1.15 min, f) mass spectrum of the peak at Rt = 6.09 min.

4.2. CEST of 3-Yb

**Figure S10.** Z-spectra of **3-Yb** (30 mM in water with 10% D_2_O, pH 7.4), B0 = 11.7 T, satpwr = 28 dB, satdly = 2 s) at T = 37 °C, time points: 0 min, 60 sec, 120 sec, 180 sec and 360 sec and 720 sec. Irradiation with 400 nm light.

**Figure S11.** Z-spectra of **3-Yb** (30 mM in water with 10% D_2_O, pH 7.4, B0 = 11.7 T, satpwr = 28 dB, satdly = 2 s) at T = 37 °C, time points: t= 0 min and t= 2h in the dark.


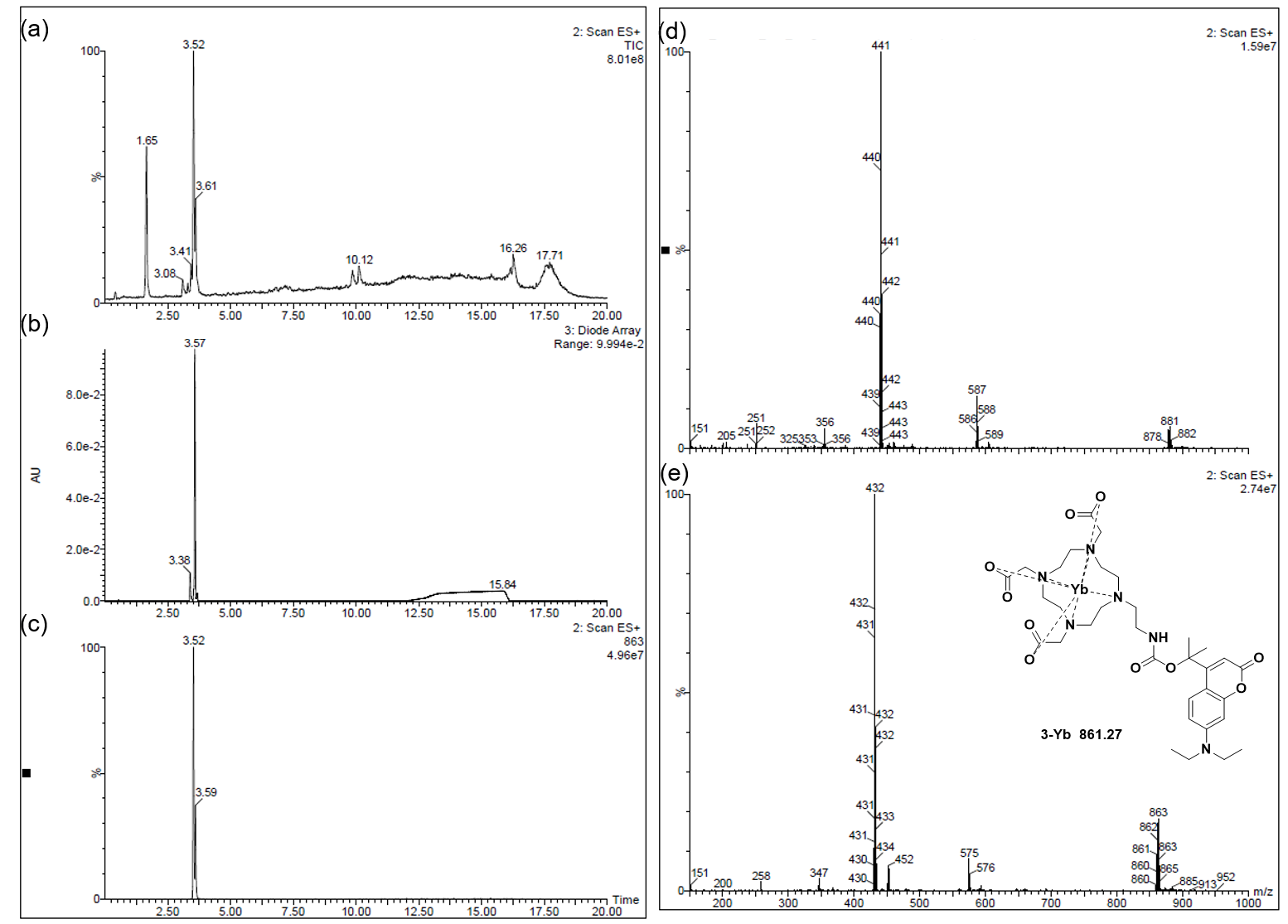


**Figure S12.** UPLC-MS analysis of **3-Yb** (the blank t=0 sec) using UPLC-MS program 3 (see section 1), a) total ion current chromatogram (positive mode), b) UV-vis at 390 nm chromatogram, c) 861-863 mass trace, d) mass spectrum of the impurity mass +18 at Rt = 1.65 min, f) mass spectrum of the peak at Rt = 3.52 min.


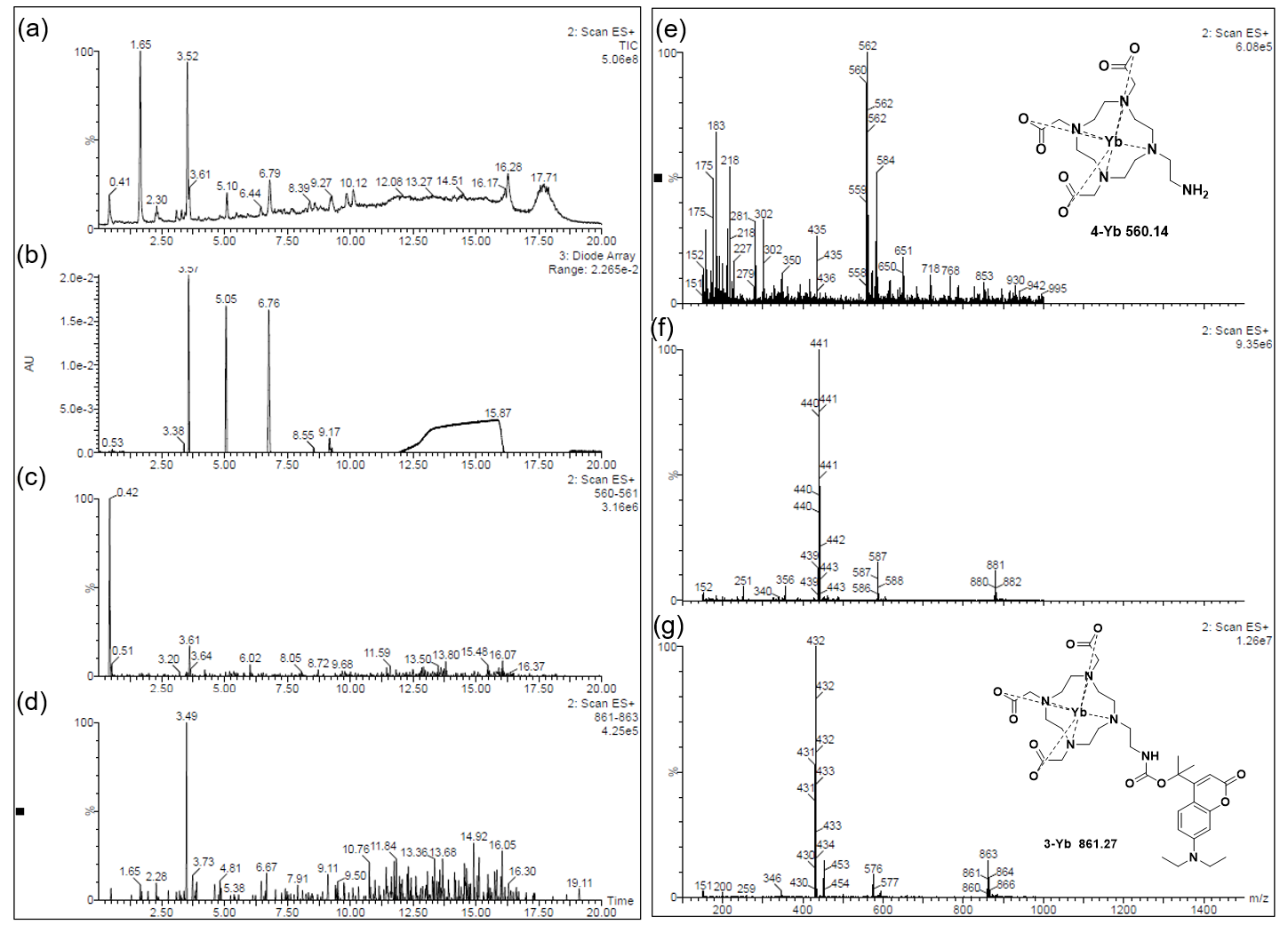


**Figure S13.** UPLC-MS analysis of **3-Yb** (t=720 sec) using UPLC-MS program 3 (see section 1), a) total ion current chromatogram (positive mode), b) UV-vis at 390 nm chromatogram, c) 560-561 mass trace, d) 861-863 mass trace, e) mass spectrum of the peak at Rt = 0.41 min, f) mass spectrum of the impurity mass +18 at Rt = 1.65 min, g) mass spectrum of the peak at Rt = 3.52 min.

5. Determination of free Yb^3+^ and Gd^3+^ concentration:

A previously published assay was adapted for the determination of free metal concentration.^5,7^ The concentration of free Yb^3+^ and Gd^3+^ was quantified by determination of the ratio of absorbance intensity at λ= 573 nm and λ = 433 nm of an Yb-xylenol orange complex and Gd-xylenol orange complex in ammonium acetate buffer (100 mM, pH 5.8, 0.60 mM xylenol orange) using a microplate reader. The contrast agents were diluted with ammonium acetate buffer before analysis.

5.1. Analysis of compound 1-Yb and 1-Gd

For the analysis the samples of **1-Yb** (50 mM concentration from the Z-spectra measurement before and after irradiation with light) and **1-Gd** (1 mM from the NMRD profiles measurements before and after irradiation with light) were diluted to a 20 μM concentration, the respectively free Yb^+3^ and Gd^+3^ was determined to be around 2-4% of each sample. All samples were measured in triplicate.


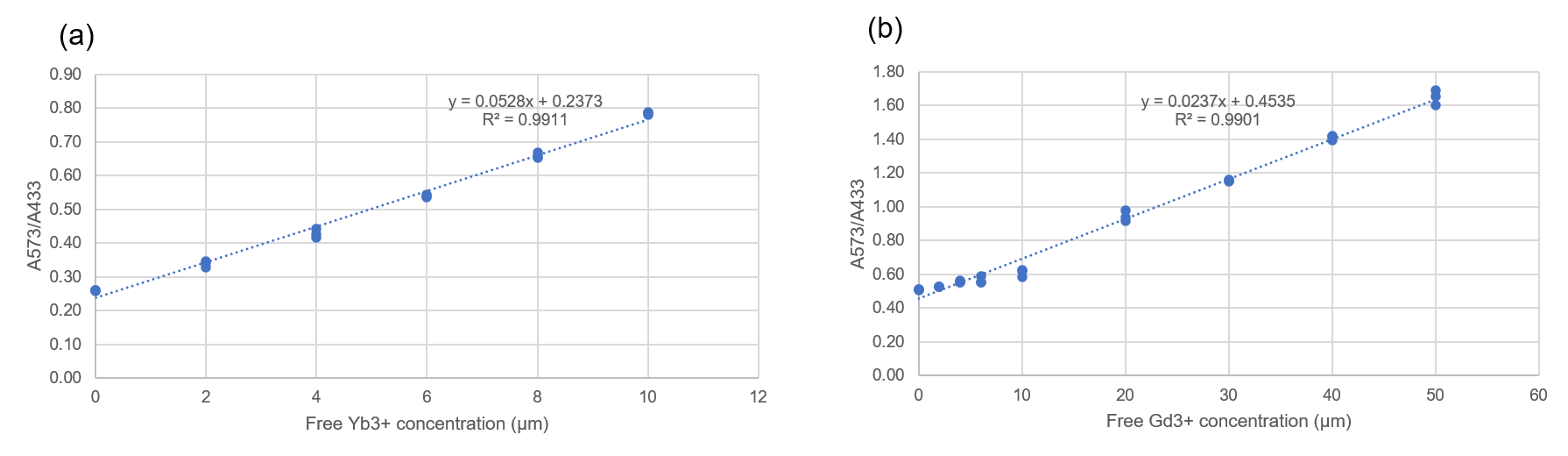


**Figure S14**. Quantification of free Yb^3+^ and Gd^3+^ for compound **1-Yb** and **1-Gd**. a) Calibration curve showing the ratio of absorbance intensity at λ = 573 nm and λ = 433 nm for increasing Yb^+3^ concentration in the presence of xylenol orange (0.60 mM). b) Calibration curve showing the ratio of absorbance intensity at λ = 573 nm and λ = 433 nm for increasing Gd^+3^ concentration in the presence of xylenol orange (0.60 mM).

Table S8. Results of the determination of free metal concentration. Column 1: sample name, column 2: ratio of absorbance intensity at λ = 573 nm and λ = 433 nm. Column 3: calculated free lanthanide concentration in μM. Column 4: recalculated concentration of free metal, taking into account the dilution of the sample. Column 5: % of free lanthanide in the sample.

| Sample | A573/A433 | uM | recalculated dilution | Free Ln^3+^ % |
| --- | --- | --- | --- | --- |
| 1-Yb | 0.2683 | 0.5878 | 587.8297 | 1.96 |
| 1-Yb | 0.2680 | 0.5816 | 581.6244 | 1.94 |
| 1-Yb | 0.2680 | 0.5816 | 581.6244 | 1.94 |
| 1-Yb after irradiation | 0.2874 | 0.9483 | 948.3020 | 3.16 |
| 1-Yb after irradiation | 0.2896 | 0.9912 | 991.1770 | 3.30 |
| 1-Yb after irradiation | 0.2969 | 1.1280 | 1127.9823 | 3.76 |
| 1-Gd | 0.4698 | 0.6864 | 15.00197 | 1.50 |
| 1-Gd | 0.4753 | 0.9189 | 26.29608 | 2.60 |
| 1-Gd | 0.4742 | 0.8728 | 8.981638 | 0.89 |
| 1-Gd after irradiation | 0.4606 | 0.3000 | 34.3195 | 3.43 |
| 1-Gd after irradiation | 0.4659 | 0.5259 | 45.94748 | 4.59 |
| 1-Gd after irradiation | 0.4578 | 0.1796 | 43.64106 | 4.36 |

5.2. Analysis of compound 3-Yb and 3-Gd


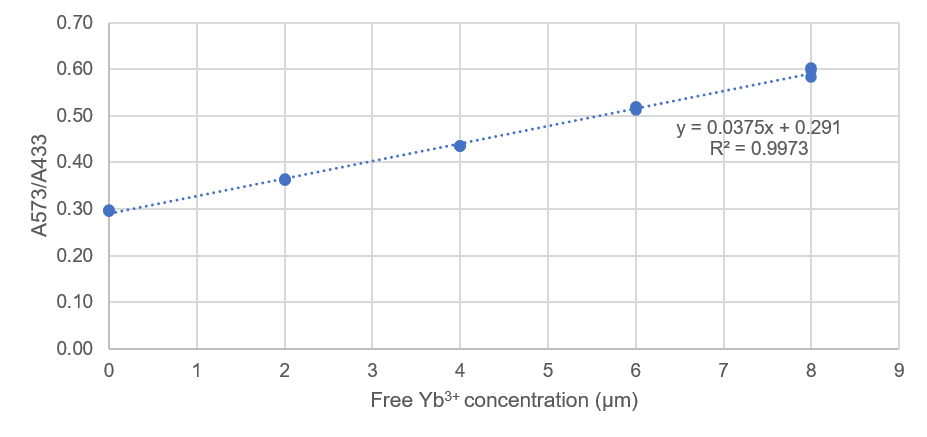


**Figure S15**. Quantification of free Yb^3+^ for compound **3-Yb**. Calibration curve showing the ratio of absorbance intensity at λ = 573 nm and λ = 433 nm for increasing Yb^+3^ concentration in the presence of xylenol orange (0.60 mM).

For the analysis the samples of **3-Yb** (30 mM concentration from the Z-spectra measurement before and after light irradiation) was diluted to a 20 μM concentration, the respectively free Yb^3+^ was determined to be around 2% of each sample. All samples were measured in triplicate.

Table S9. Results of the determination of free metal concentration. Column 1: sample name, column 2: ratio of absorbance intensity at λ = 573 nm and λ = 433 nm. Column 3: calculated free lanthanide concentration in μM. Column 4: recalculated concentration of free metal, taking into account the dilution of the sample. Column 5: % of free lanthanide in the sample.

| Sample | A573/A433 | μM | recalculated dilution | Free Ln^3+^ % |
| --- | --- | --- | --- | --- |
| 3-Yb | 0.3064 | 0.4118 | 411.8177 | 2.06 |
| 3-Yb | 0.3095 | 0.4946 | 494.6345 | 2.47 |
| 3-Yb | 0.3080 | 0.4537 | 453.7245 | 2.27 |
| 3-Yb after irradiation | 0.2941 | 0.0815 | 81.4869 | 0.41 |
| 3-Yb after irradiation | 0.3058 | 0.3946 | 394.6092 | 1.97 |
| 3-Yb after irradiation | 0.3032 | 0.3266 | 326.6426 | 1.63 |

6. UV-Vis absorption spectroscopy

UV-Vis absorption spectra are recorded via a similar procedure as previously described by Schulte et al. ^6^ A stirred 2 mL solution of a compound (100 μM for **1-Yb** and **1-Gd** and 20 μM for **3-Yb** ) in water was irradiated from the side in a fluorescence quartz cuvette (optical path = 1 cm), using a custom-built (Prizmatix/Mountain Photonics) multi-wavelength fiber coupled LED-system (FC6-LED-WL). The full width at half maximum (FWHM) for the 365&390 nm LED was ≤ 20 nm. The LED was connected through a 7 to 1 fiber bundle attached to a 3 mm liquid light guide (LLG-3) and a liquid light guide adapter (LLG-AC). The adapter was placed in a Thorlabs SMR1 lens mount which was adjusted to height using Thorlabs TR20/30 optical posts, AS6M4M adapters and a PJ302/M Offset Mounting Post Joist. The LED was controlled automatically via the built-in USB-controller using FC-LED-Ctrl 3.0 & Pulover’s MacroCreator 5.05. For all experiments, the temperature was maintained at 298.15 K using a Quantum Northwest TC1 temperature controller. Raw data was processed using Agilent UV-Vis ChemStation B.02.01 SP1, Spectragryph 1.2 and OriginPro 8.5.

**Figure S16**. UV-Vis absorption spectra of **1-Yb** (100 μM, water, 25 °C, pH 7). A freshly prepared solution (green) and solutions after irradiation (λ = 365 nm) for the times indicated.

**Figure S17**. UV-Vis absorption spectra of **1-Gd** (100 μM, water, 25 °C, pH 7). A freshly prepared solution (yellow) and solutions after irradiation (λ = 365 nm) for the times indicated.

**Figure S18.** UV-Vis absorption spectra of **3-Yb** (20 μM, water, 25 °C, pH 7 ). A freshly prepared solution (blue) and solutions after irradiation (λ = 390 nm) for the times indicated.

7. NMRs


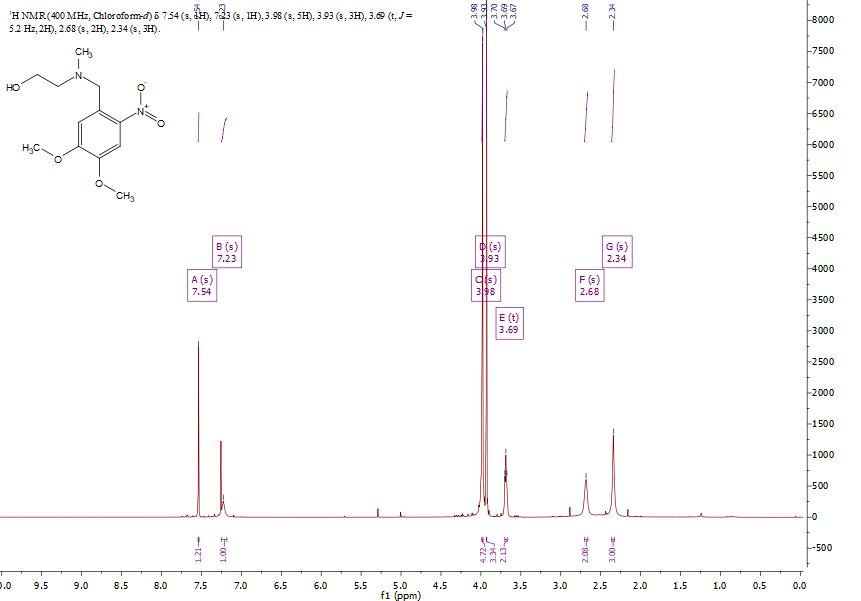


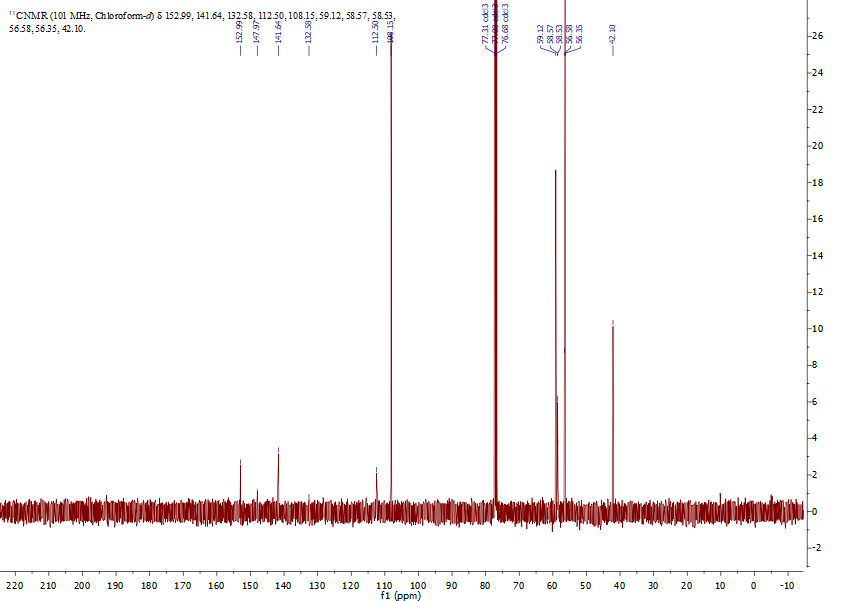
**Figure S19.** ^1^H NMR and ^13^C NMR spectra of 2-((4,5-dimethoxy-2-nitrobenzyl)(methyl)amino)ethan-1-ol (**S1**).


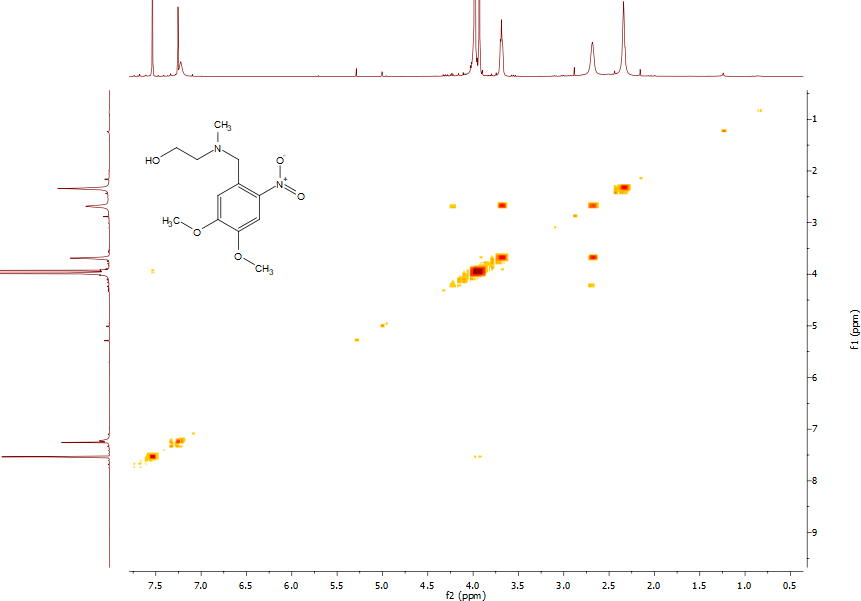


**Figure S20**. ^1^H COSY spectrum of 2-((4,5-dimethoxy-2-nitrobenzyl)(methyl)amino)ethan-1-ol (**S1**).


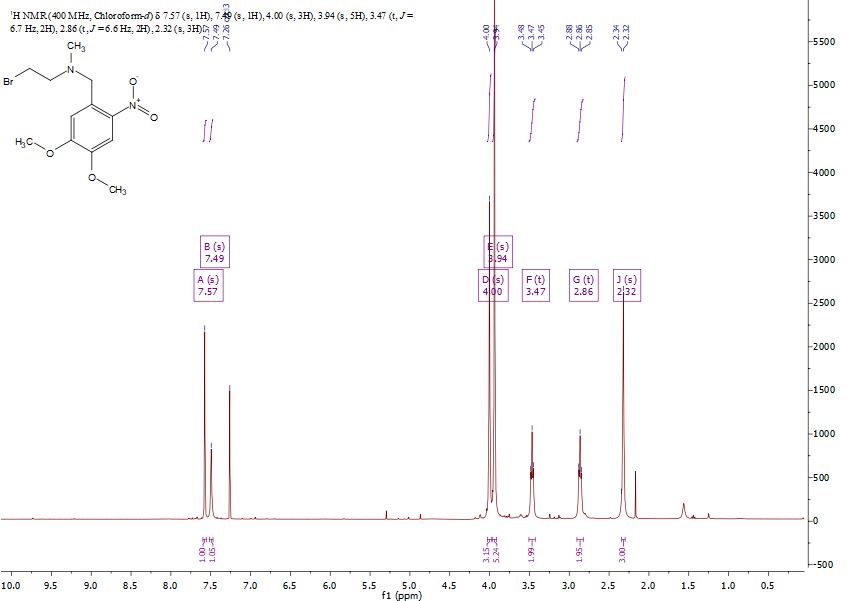


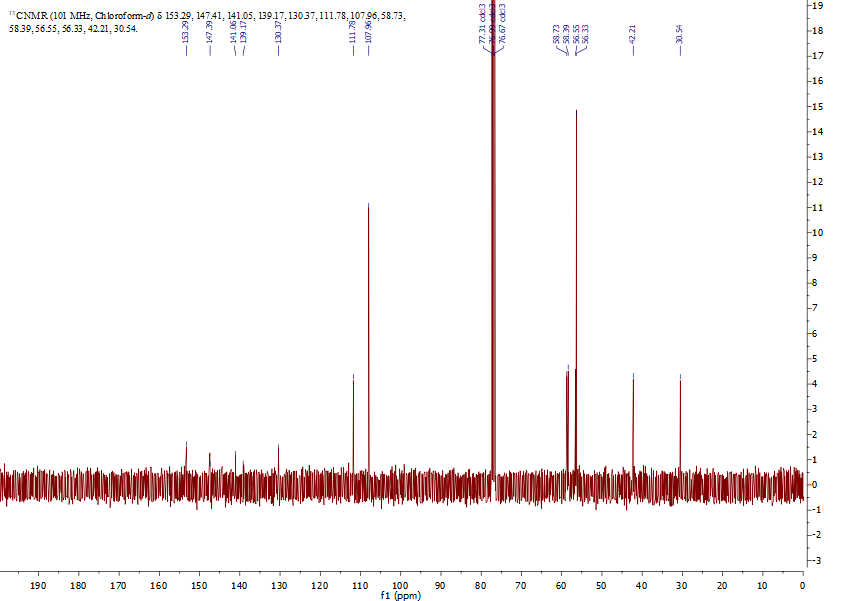


**Figure S21**. ^1^H NMR and ^13^C NMR spectra of 2-bromo-*N*-(4,5-dimethoxy-2-nitrobenzyl)-*N*-methylethan-1-amine (**5**).


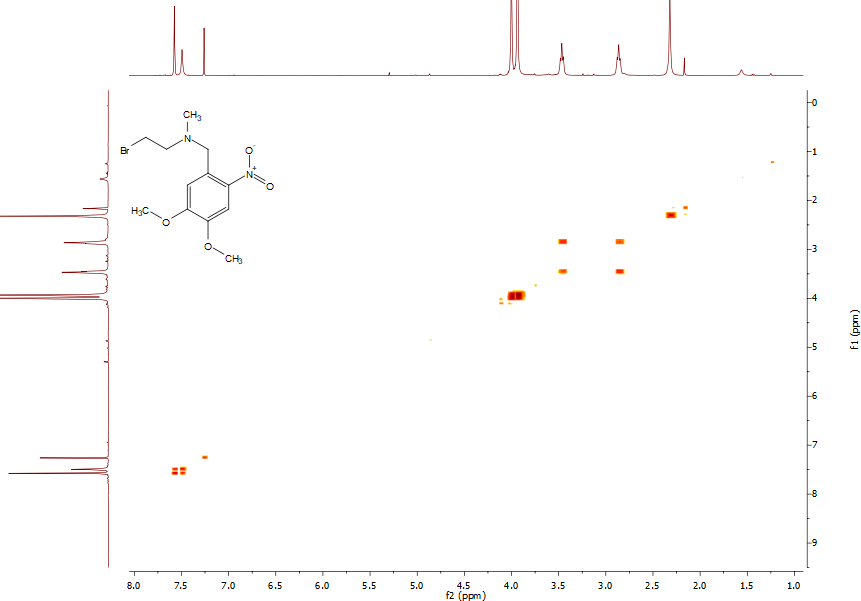


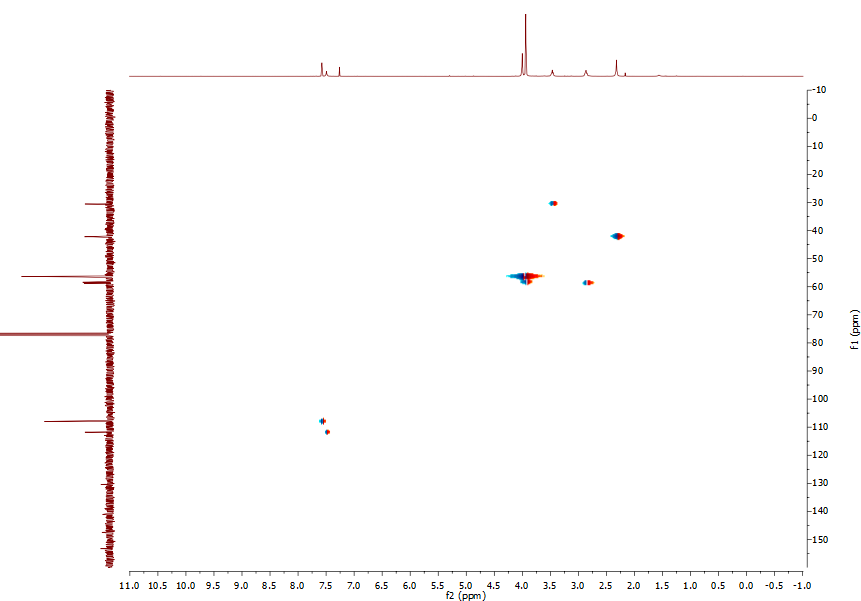


**Figure S22**. COSY and HSQC NMR spectra of 2-bromo-*N*-(4,5-dimethoxy-2-nitrobenzyl)-*N*-methylethan-1-amine (**5**).

 
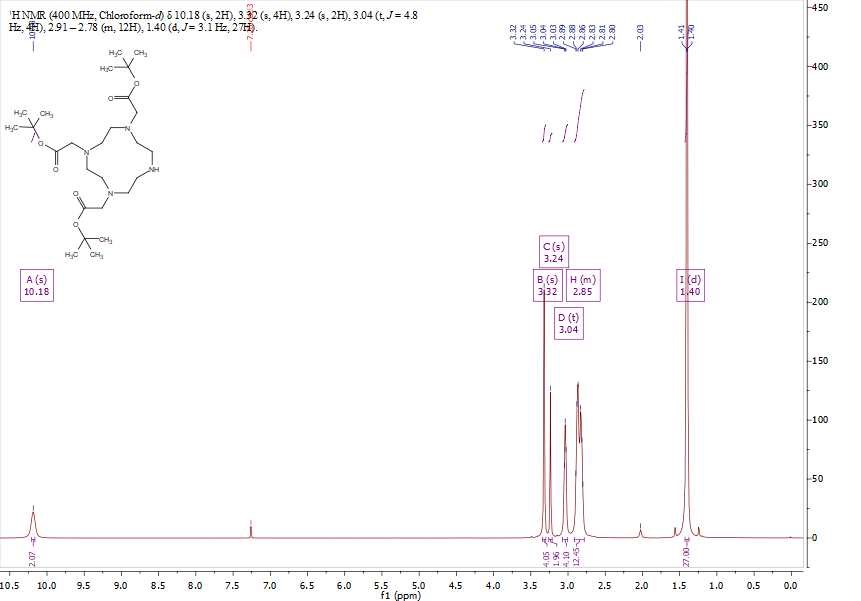


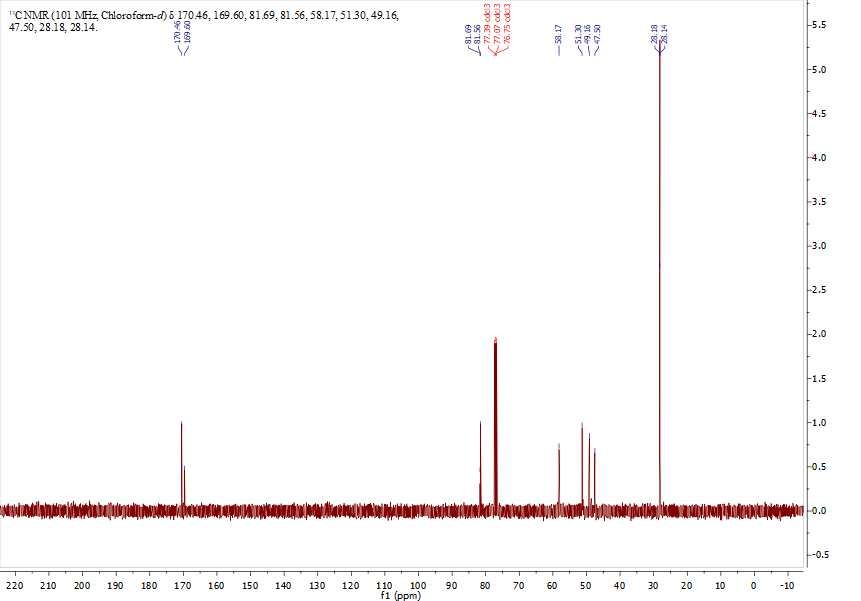


**Figure S23.** ^1^H NMR and ^13^C NMR spectra of Tri-*tert*-butyl 2,2',2''-(1,4,7,10-tetraazacyclododecane-1,4,7-triyl)triacetate (**6**).


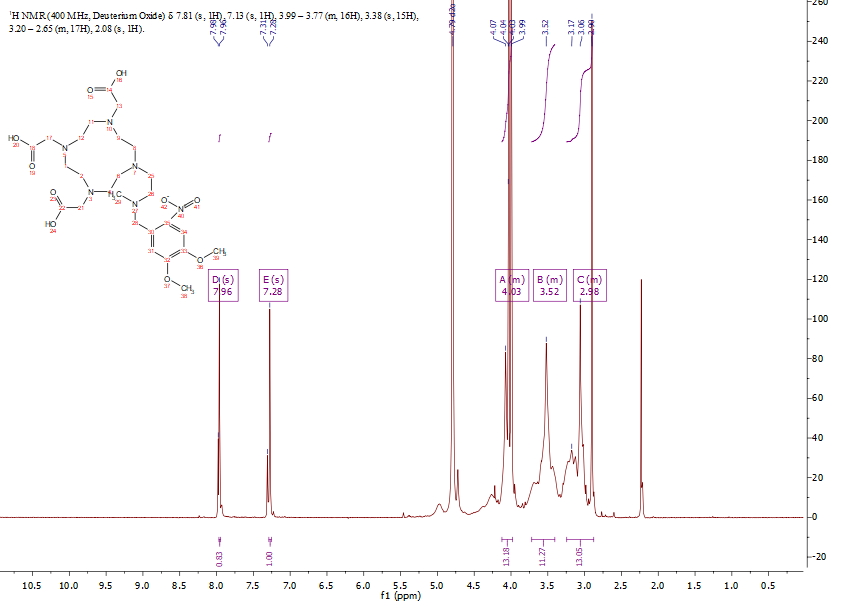


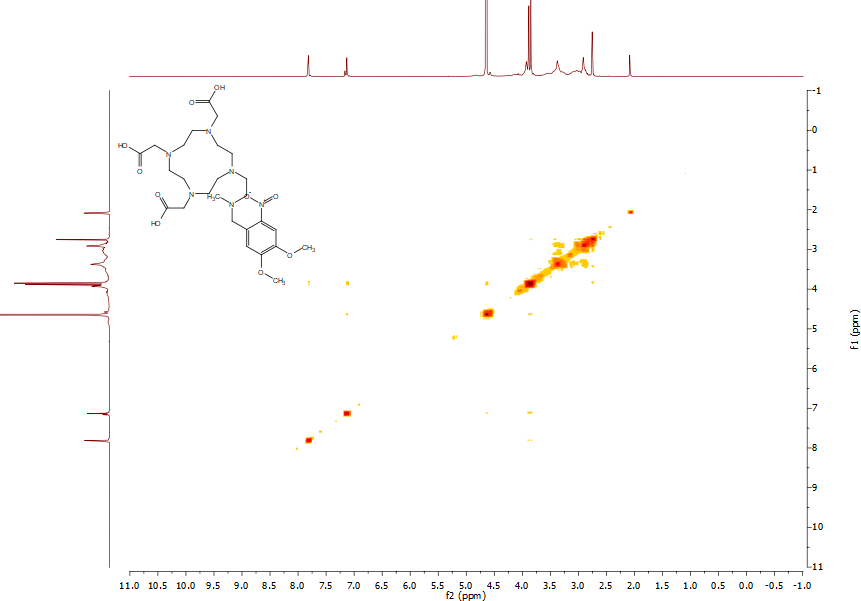


**Figure S24**. ^1^H NMR and COSY NMR spectra of 2,2',2''-(10-(2-((4,5-dimethoxy-2-nitrobenzyl)(methyl)amino)ethyl)-1,4,7,10-tetraazacyclodode-cane-1,4,7-triyl)triacetic acid (**1**).


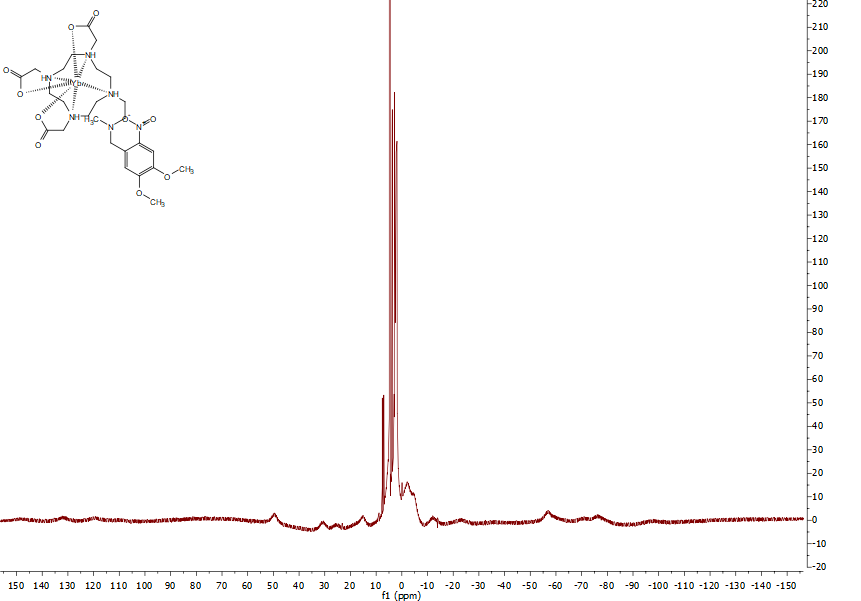


**Figure S25.** ^1^H NMR spectrum of **1-Yb**.


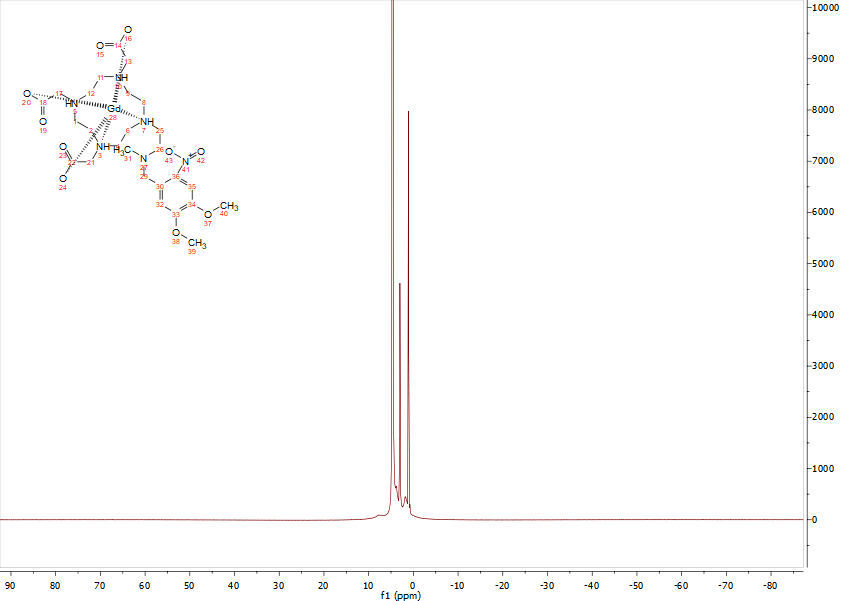


**Figure S26.** ^1^H NMR spectrum of **1-Gd**.


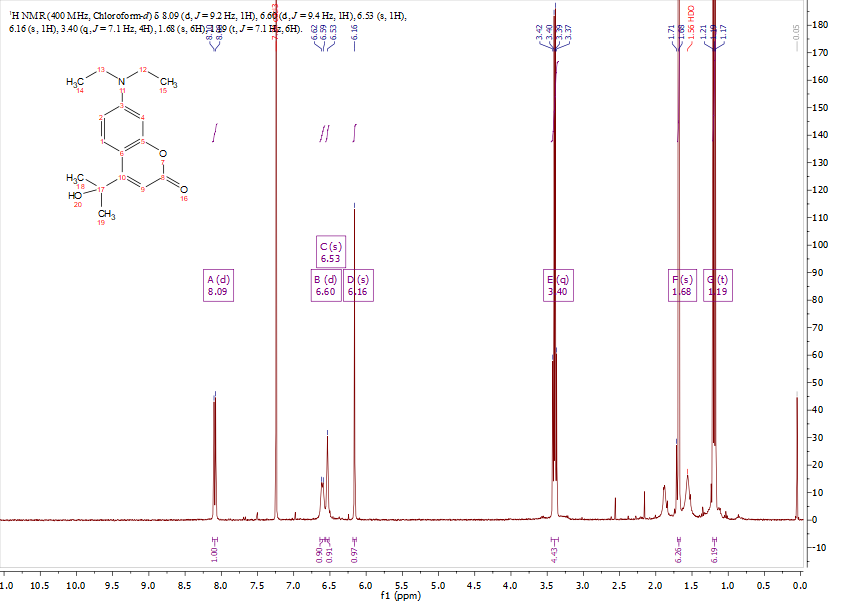


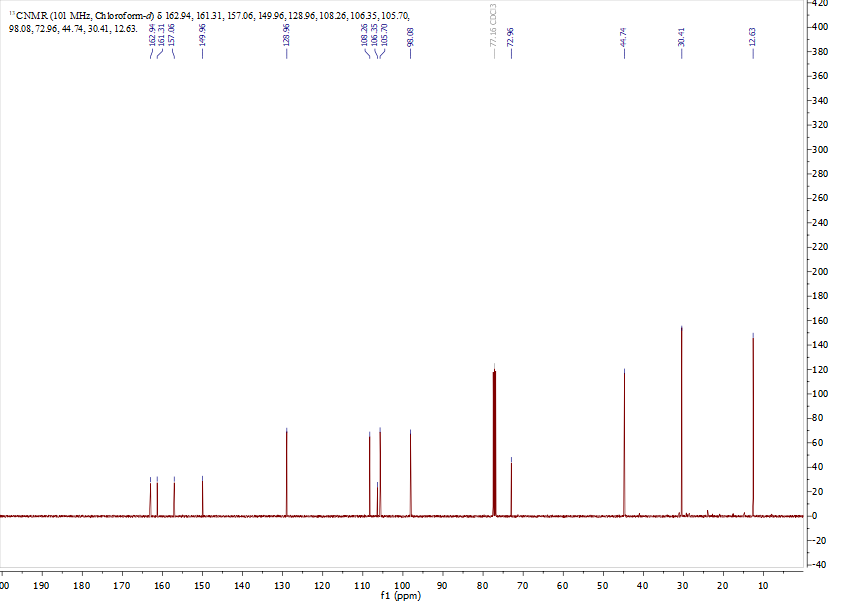


**Figure S27.** ^1^H NMR and ^13^C NMR spectra of 7-(diethylamino)-4-(2-hydroxypropan-2-yl)-2H-chromen-2-one (**8**)


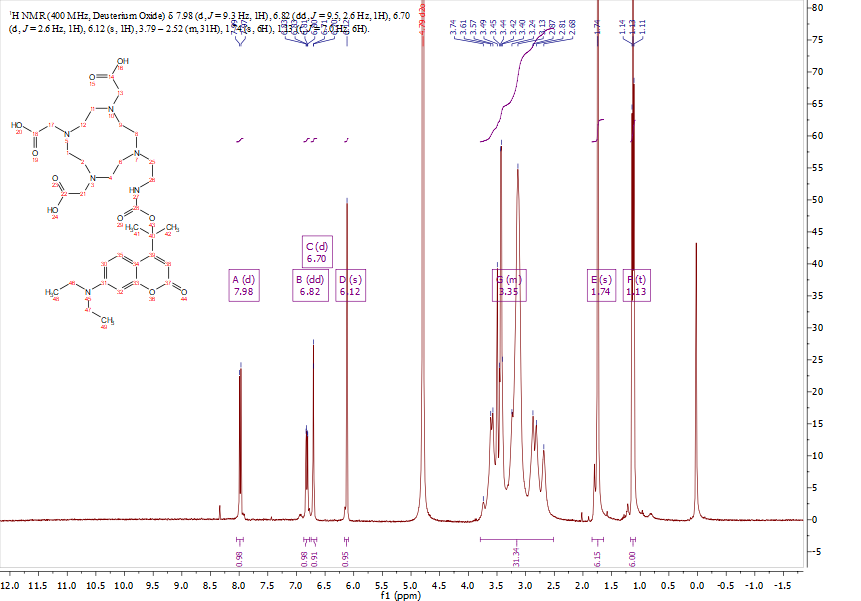


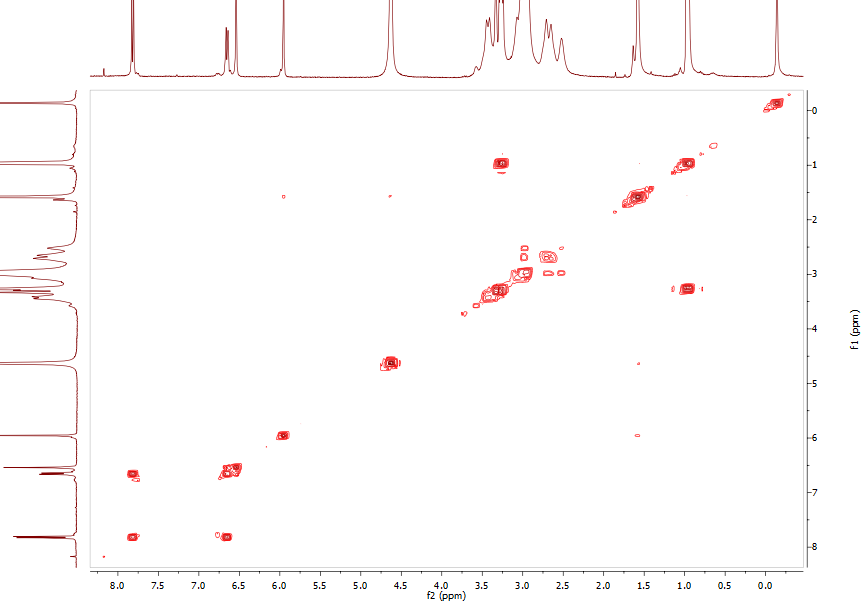


**Figure S28**. ^1^H NMR and COSY NMR spectra of 2,2',2''-(10-(2-((4,5-dimethoxy-2- nitrobenzyl)(methyl)amino)ethyl)-1,4,7,10-tetraazacyclodode-cane-1,4,7-triyl)triacetic acid (**3**).


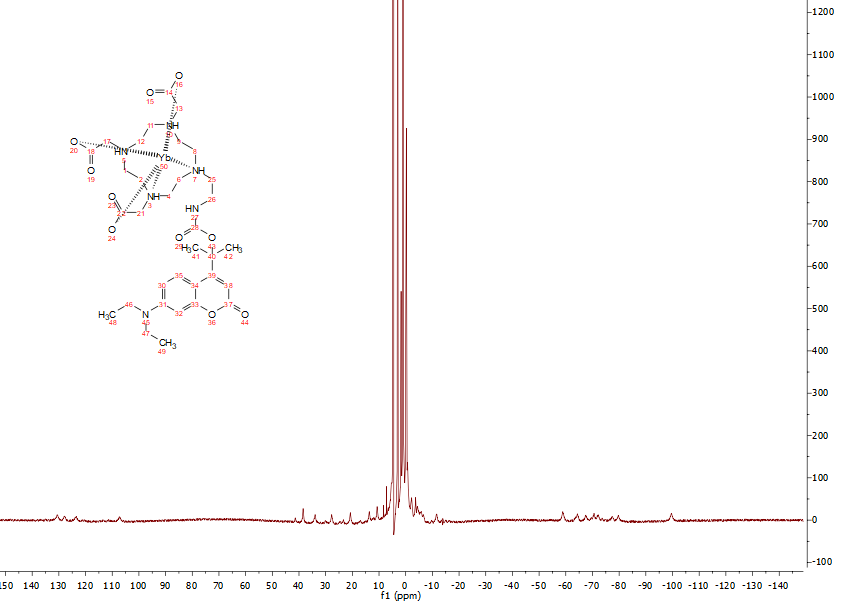


**Figure S29**. ^1^H NMR spectrum of **3-Yb**.


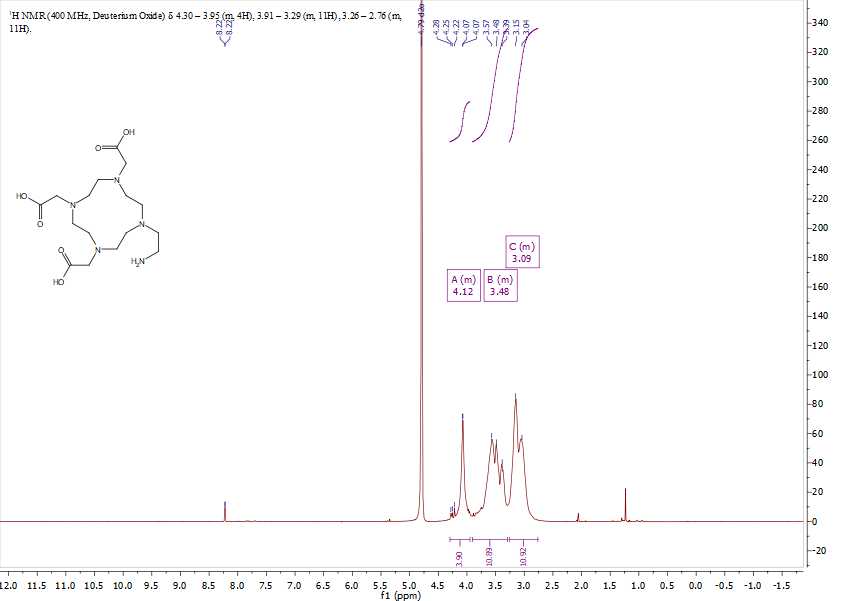


**
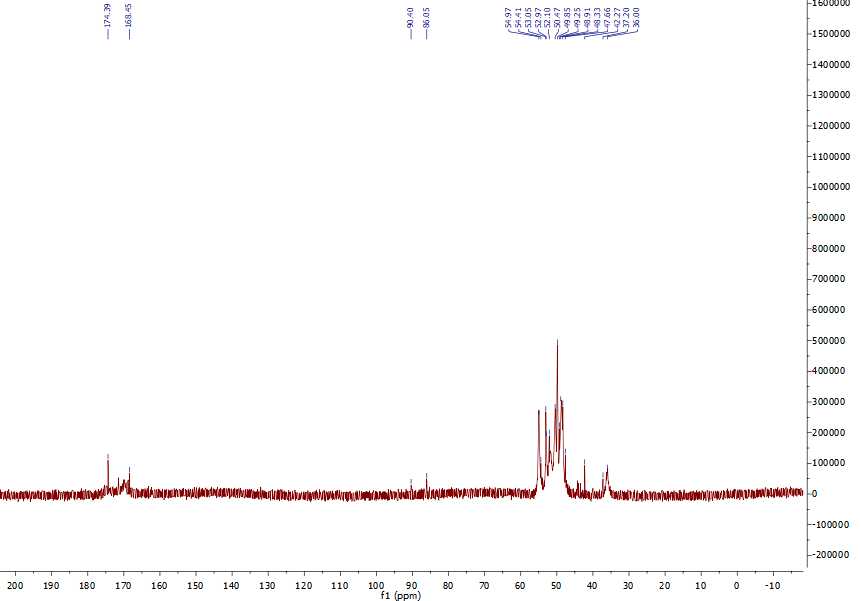
**

**Figure S30.** ^1^H NMR and ^13^C NMR of 2,2',2''-(10-(2-ammonioethyl)-1,4,7,10-tetraazacyclododecane-1,4,7-triyl)triacetate (**4**).

**
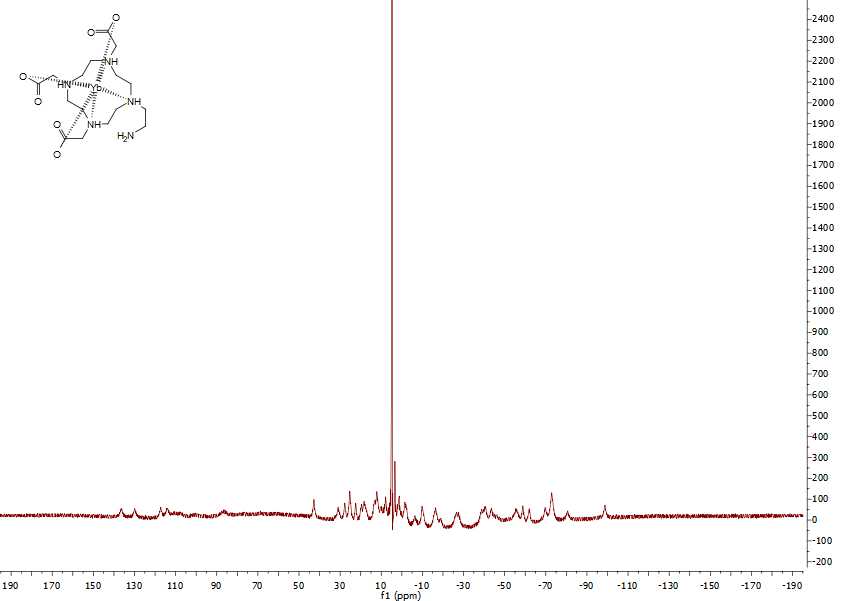
**

Figure S31. ^1^H NMR of **4-Yb**.

8. HRMS


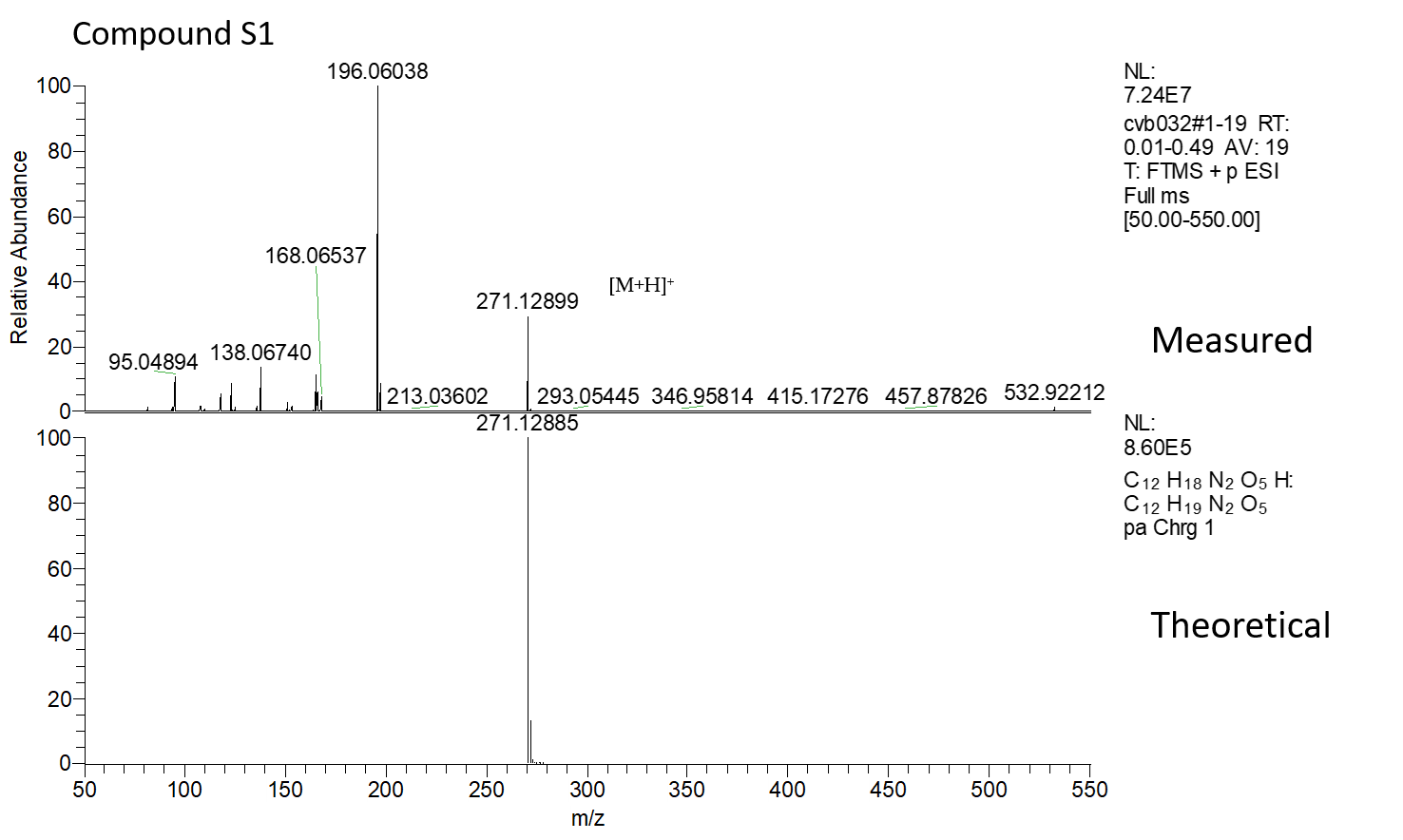


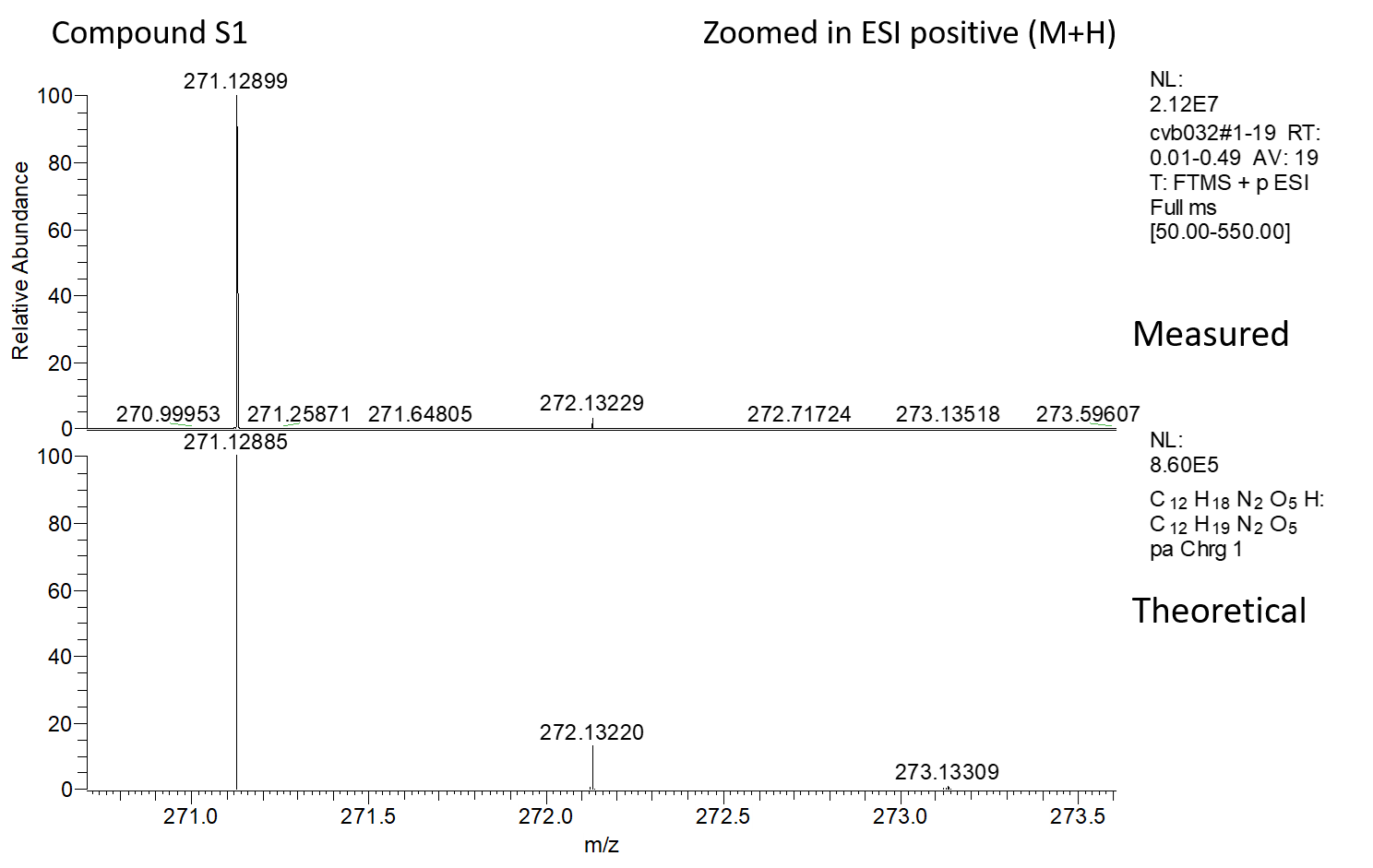


**Figure S32**, HRMS analysis of compound **S1**, HRMS (ESI+) calc. for [M+H]^+^. (C_12_H_19_O_5_N_2_^+^) 271.1288, found: 271.1289.


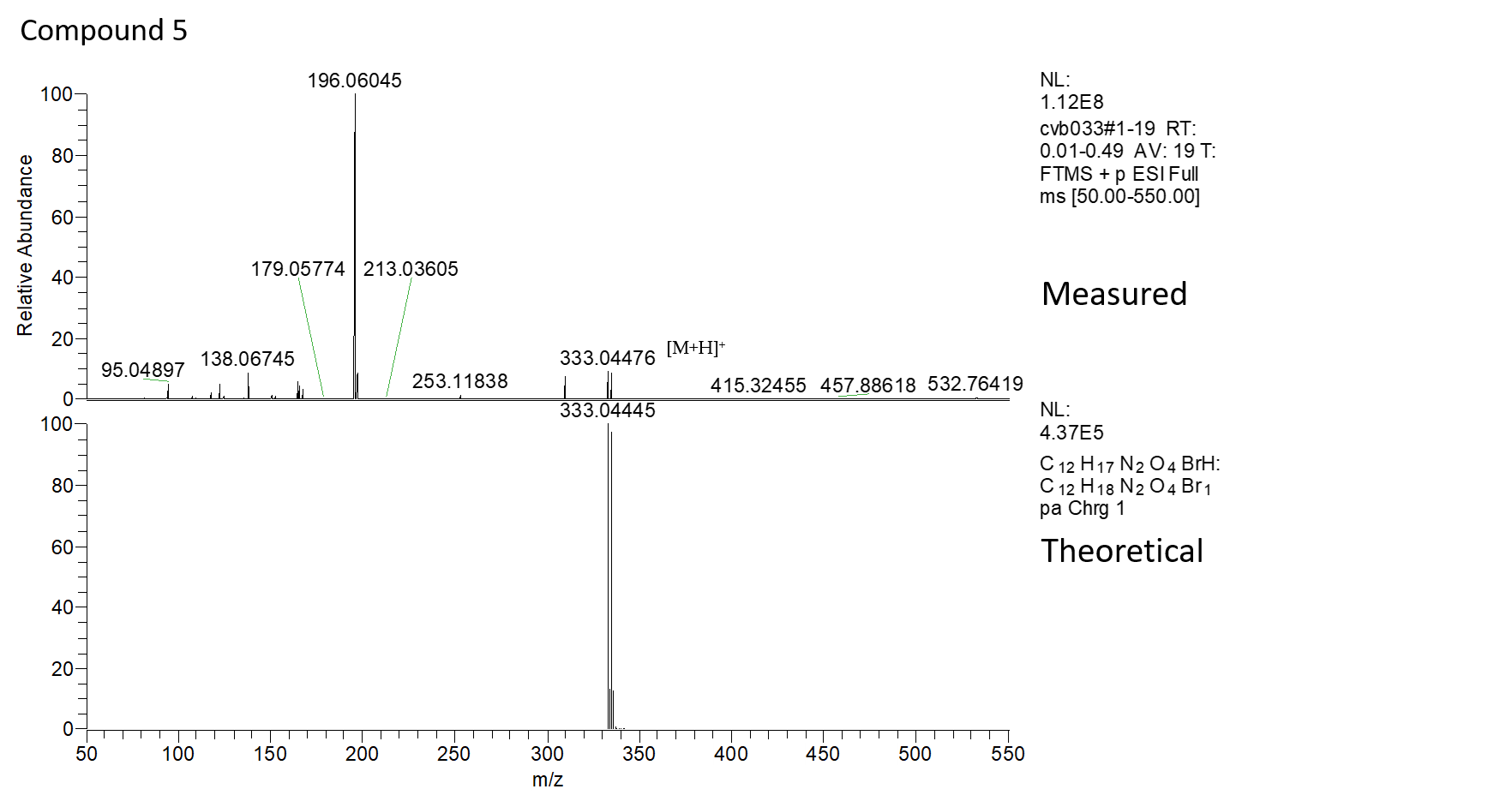


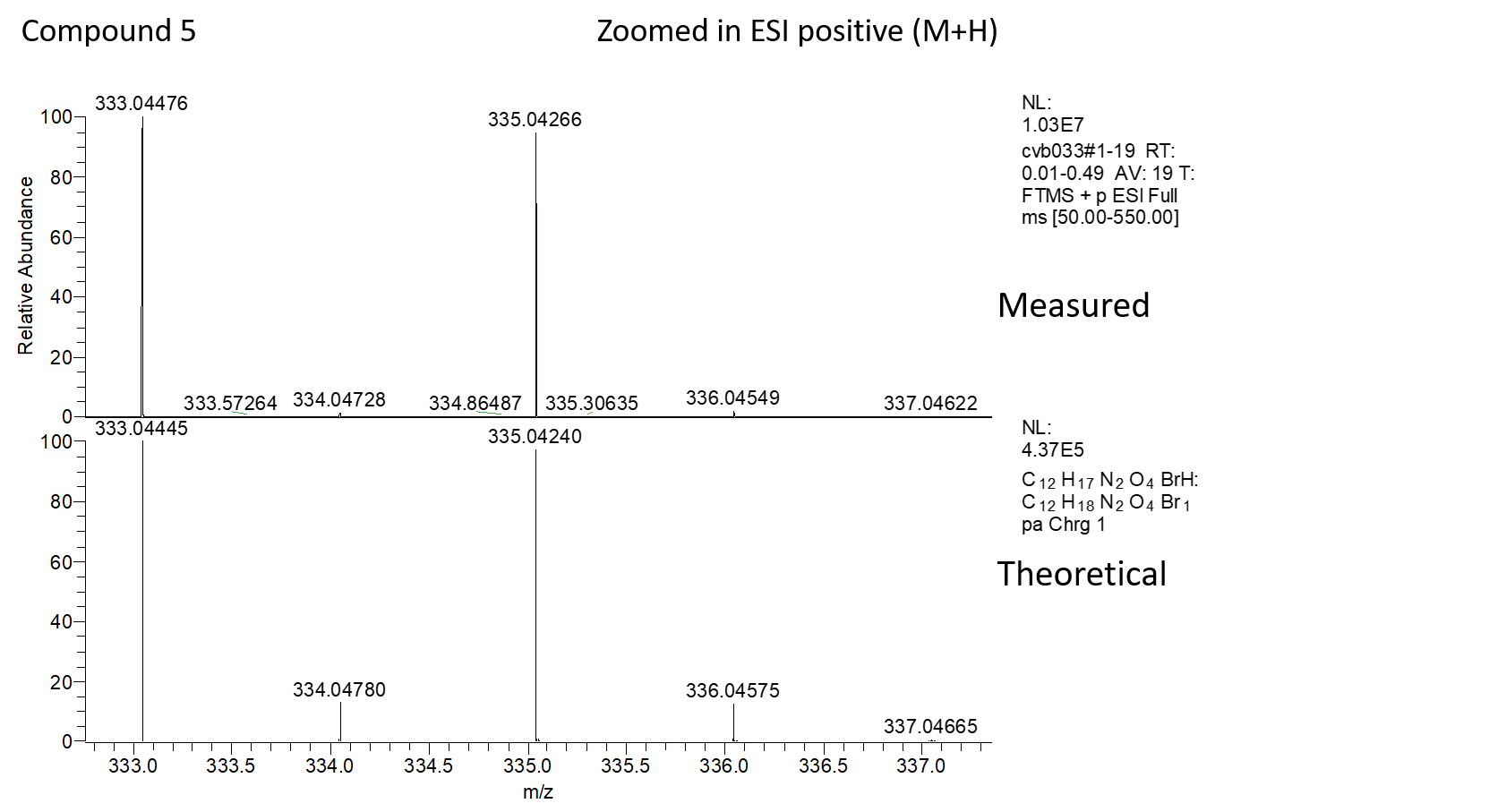


**Figure S33.** HRMS analysis of compound **5**, HRMS (ESI+) calc. for [M+H]^+^. (C_12_H_18_O4N_2_Br^+^) 335.0424, found: 335.0426


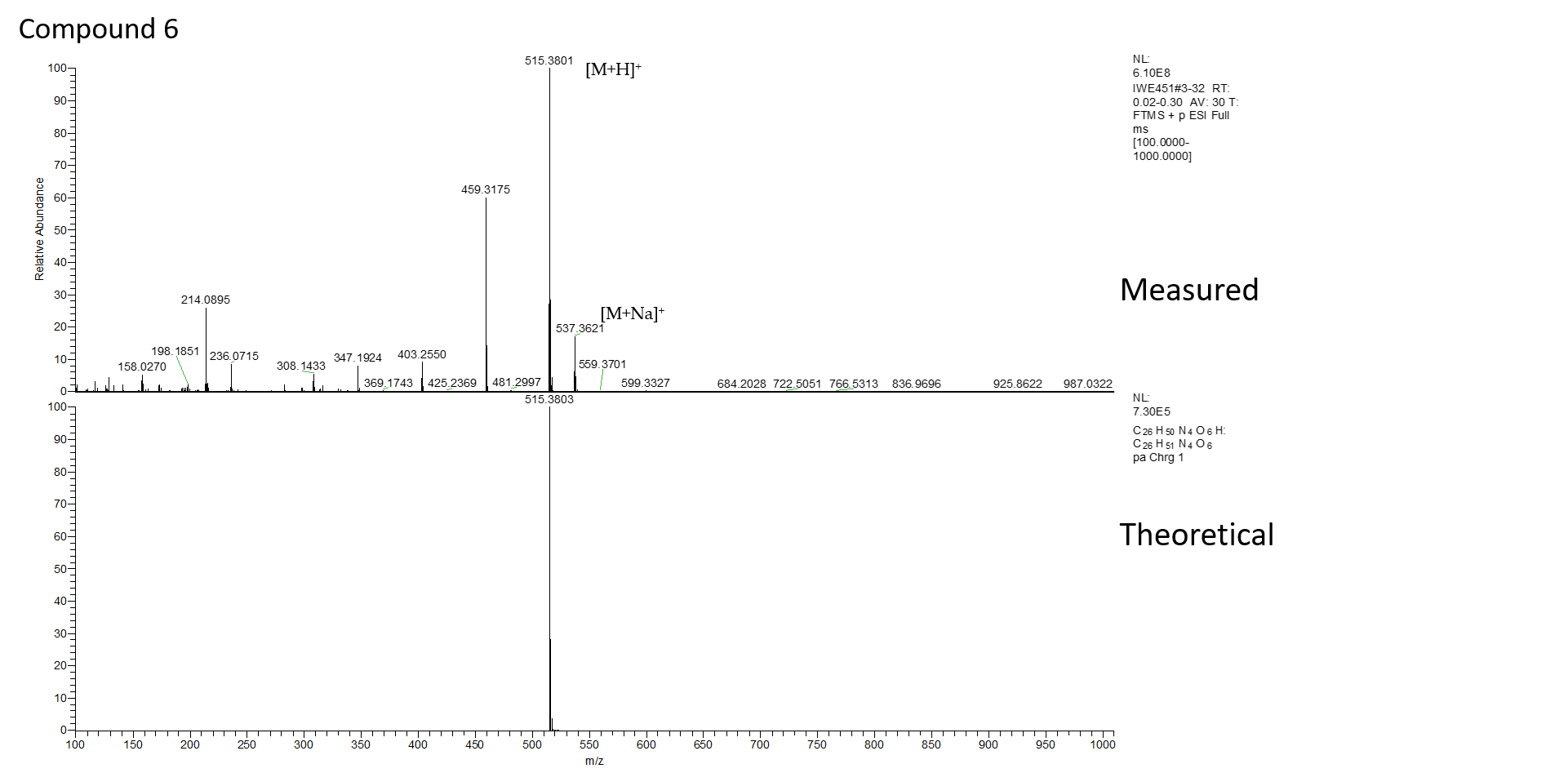


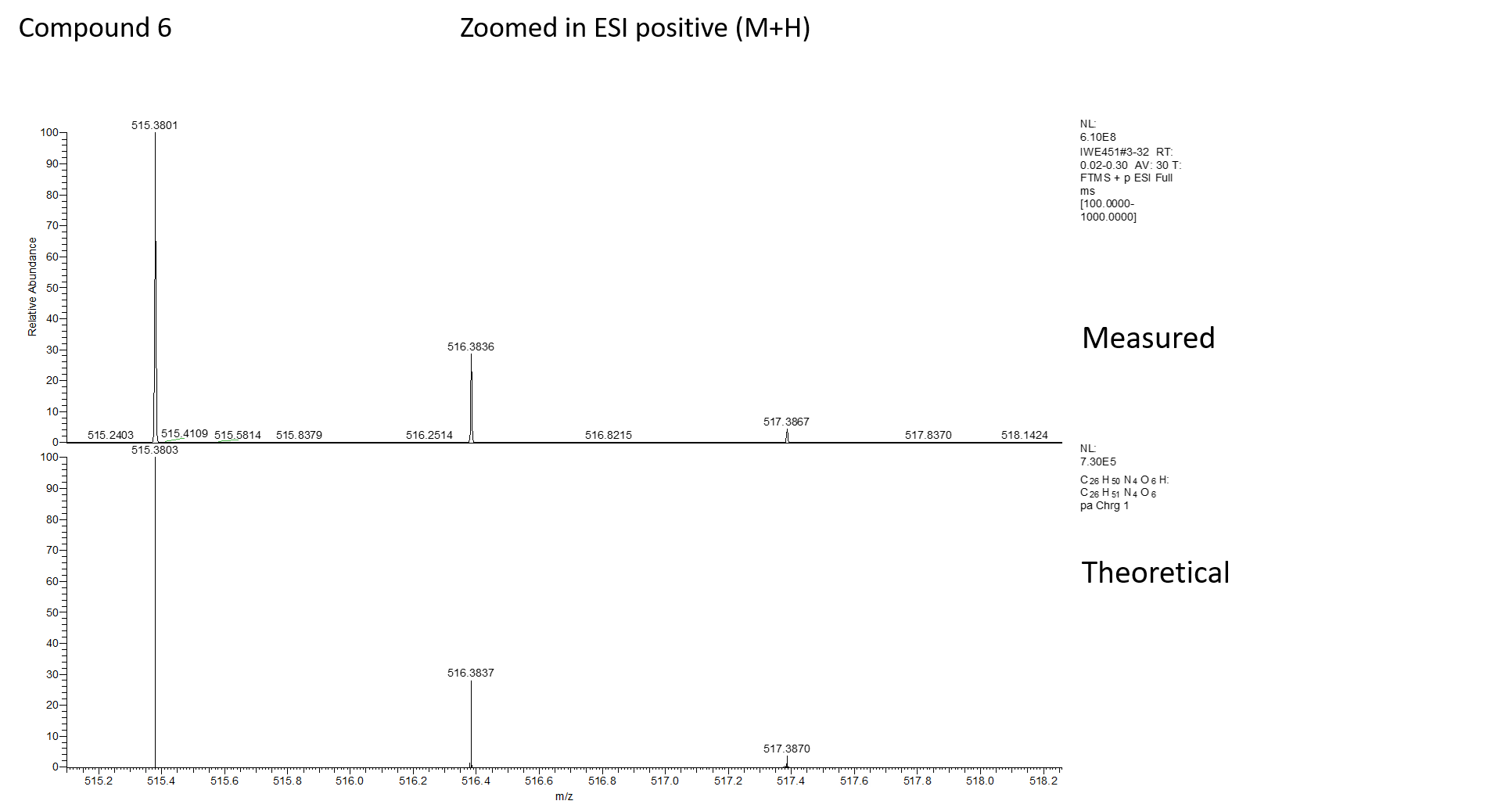


**Figure S34**. HRMS analysis of compound **6**, HRMS (ESI+) calc. for [M+H]^+^ (C_26_H_51_N_4_O_6_^+^): 515.3803, found: 515.3801.


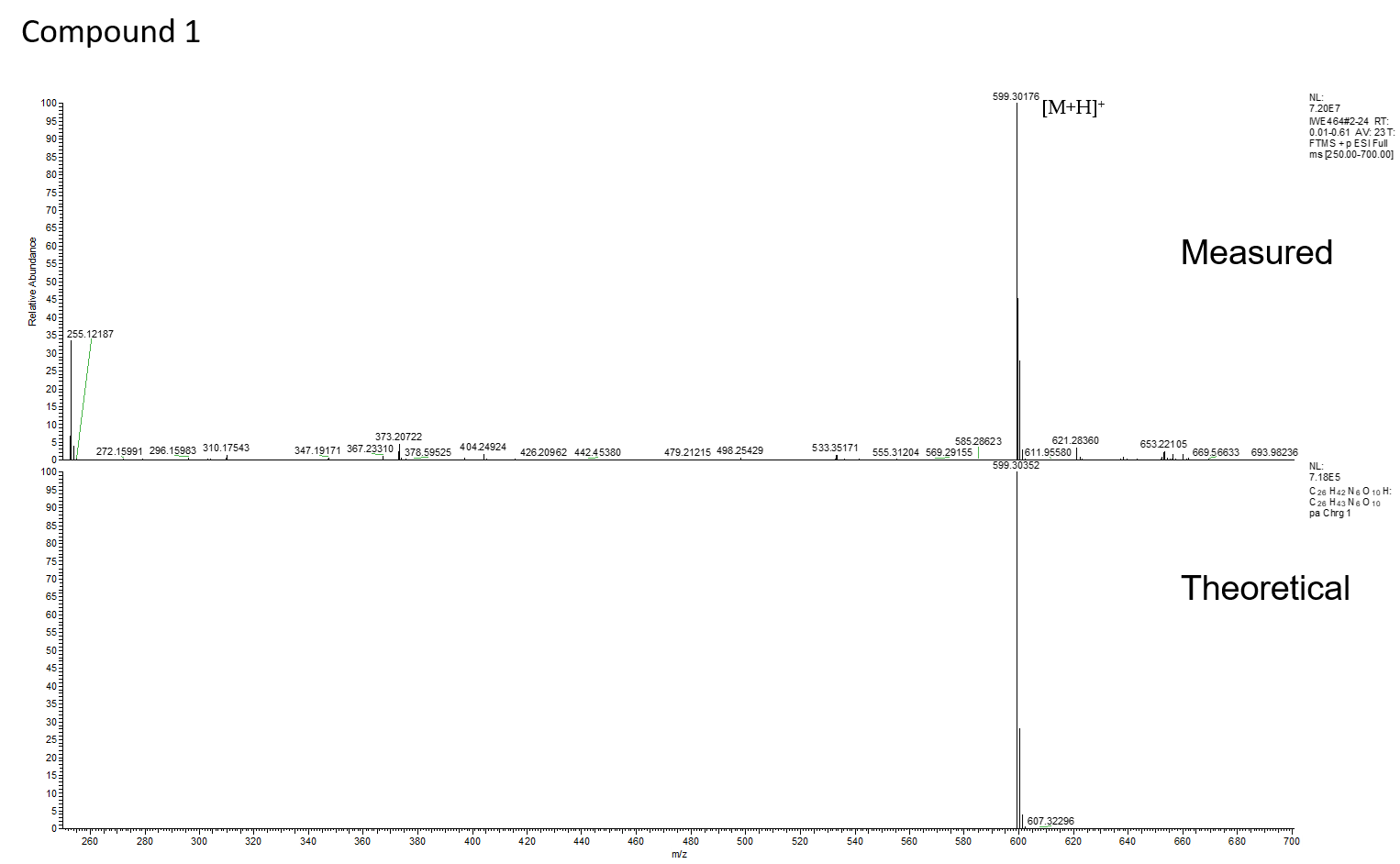


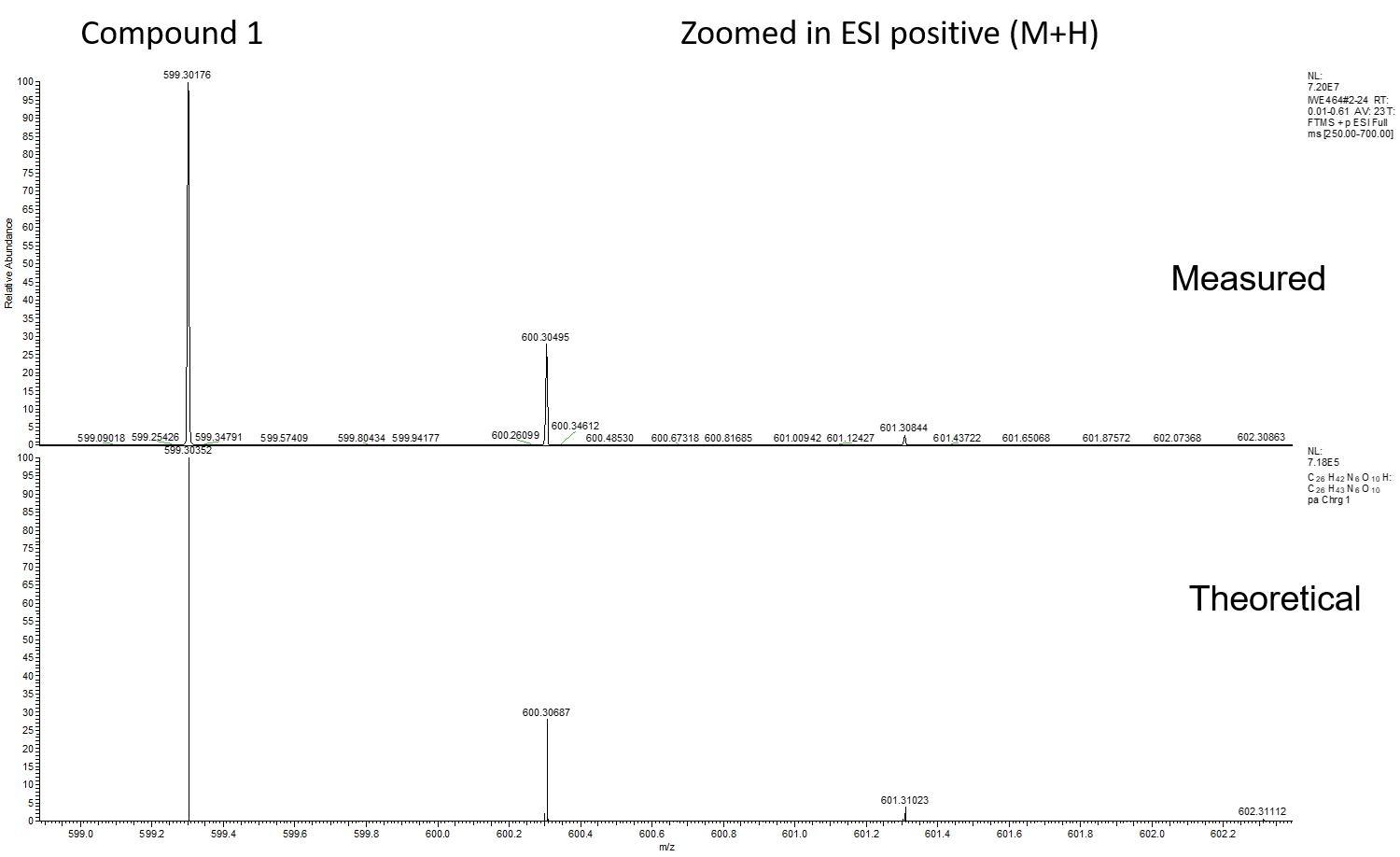


**Figure S35**. HRMS analysis of compound **1**, ESI- HRMS (ESI+) calc. for [M+H]^+^ (C_26_H_43_N_6_O_10_^+^): 599.3035, found: 599.3017.


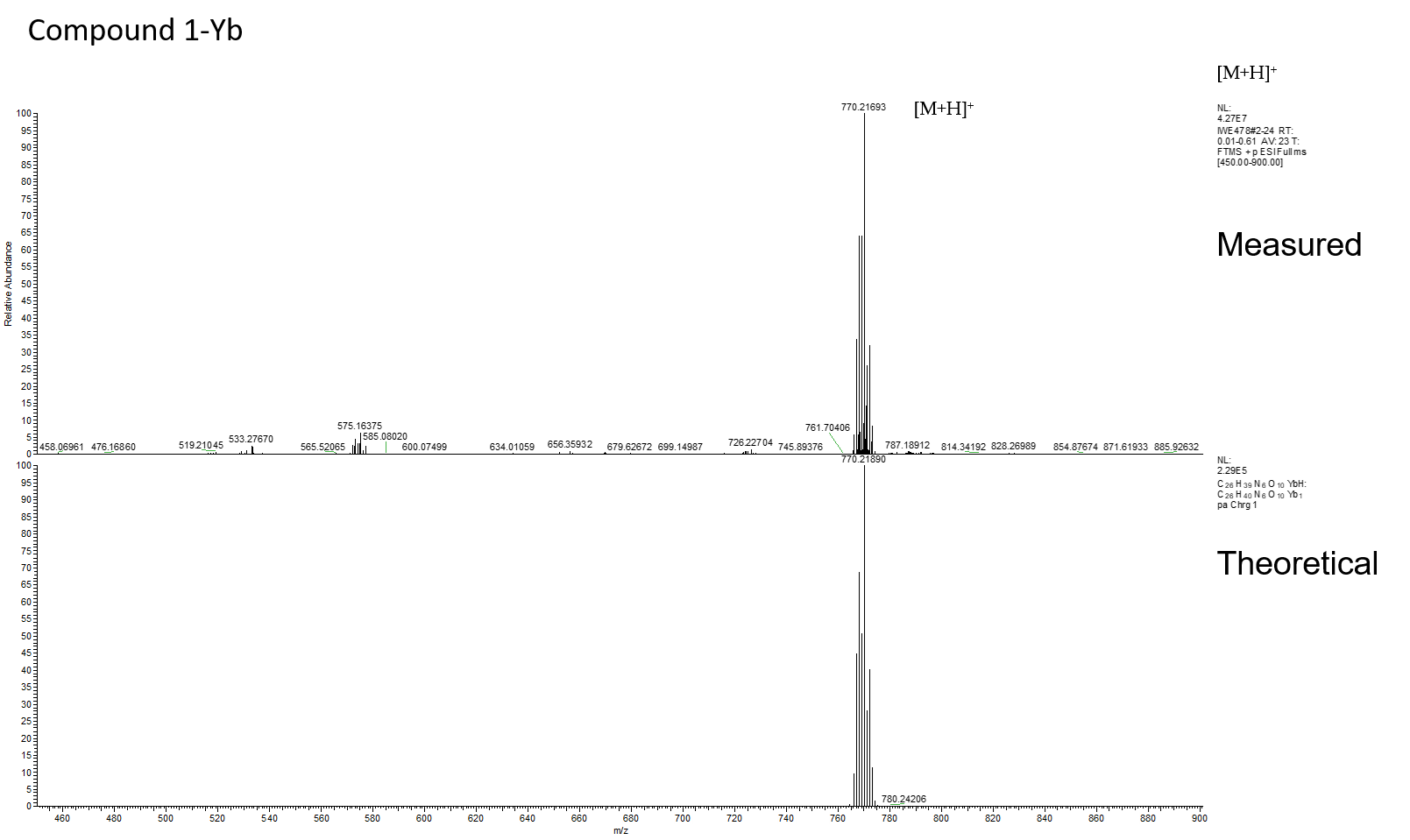


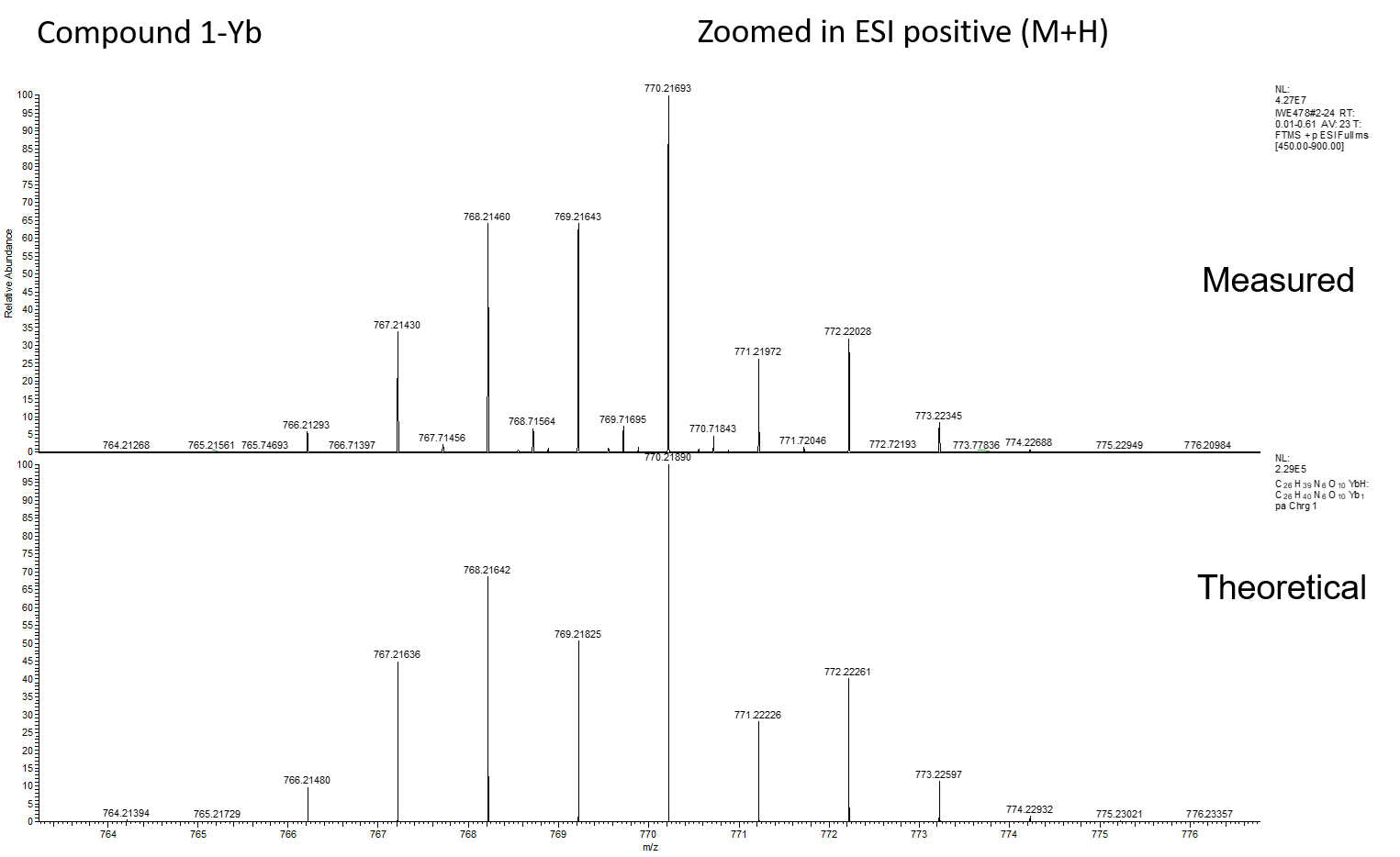


**Figure S36.** HRMS analysis of compound **1-Yb**, HRMS (ESI+) calc. for [M+H]^+^ (C_26_H_40_N_6_O_10_Yb^+^): 770.2189, found: 770.2169.


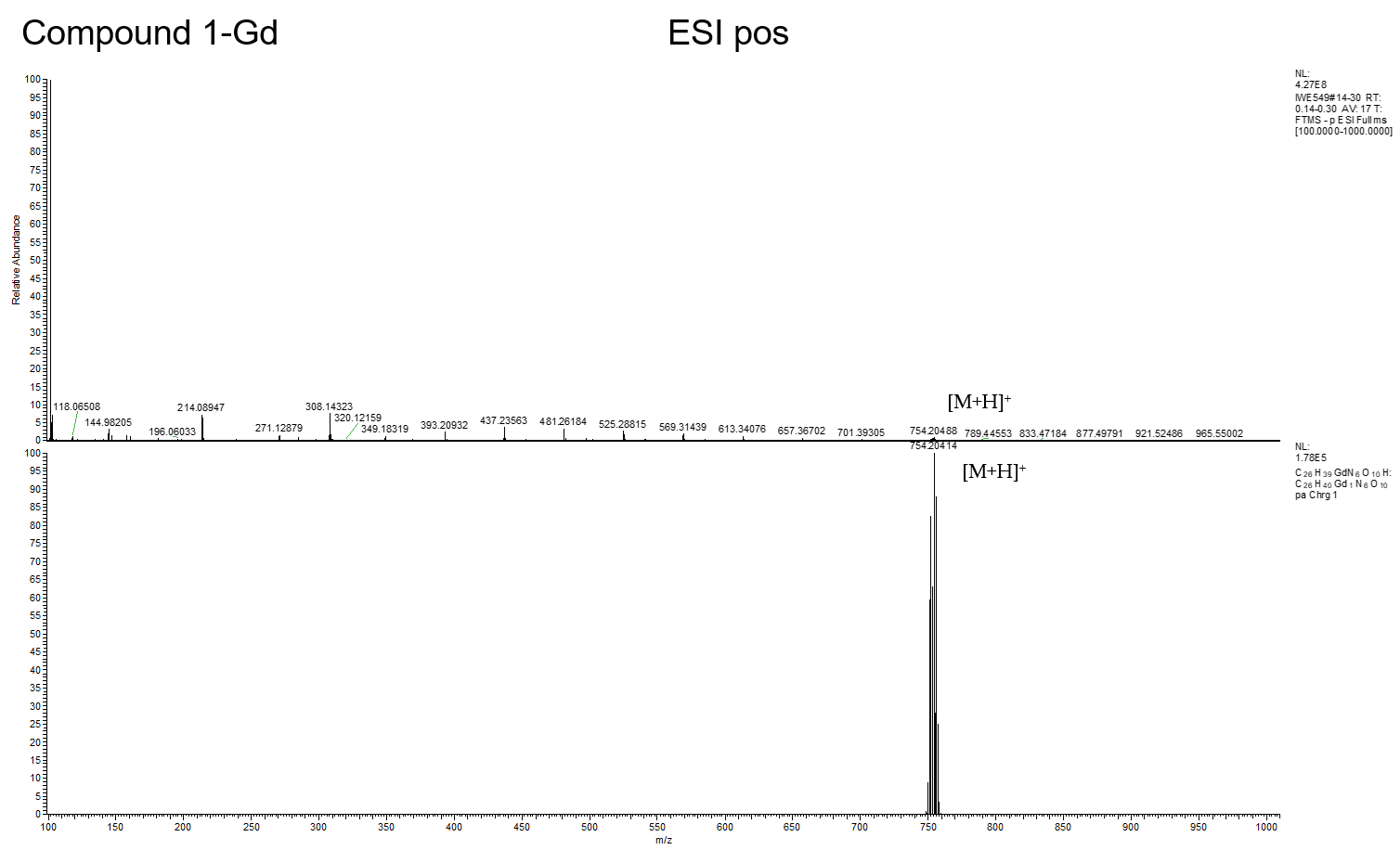


**Measured**

**Calculated**


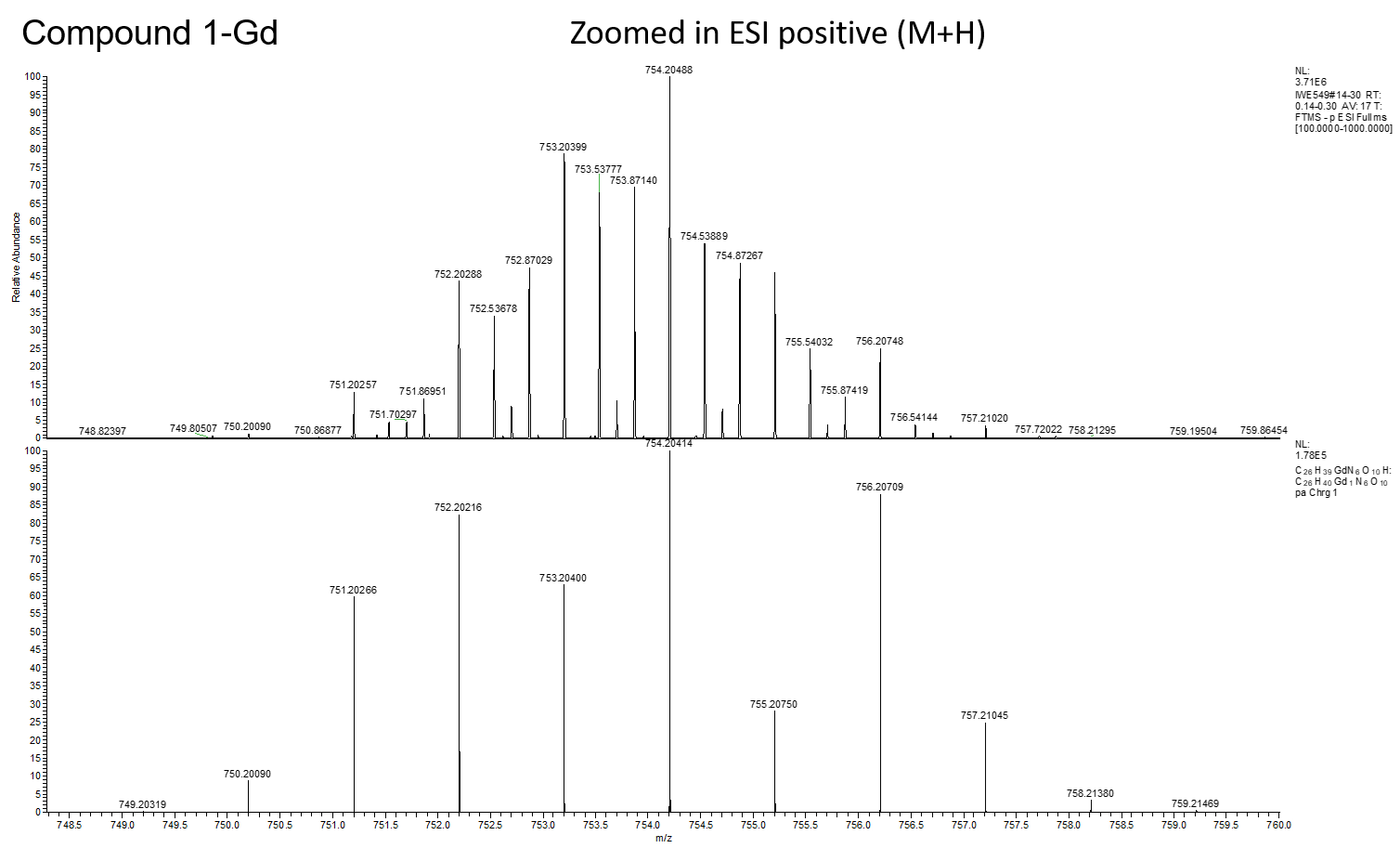


**Measured**

**Calculated**

**Figure S37**, HRMS analysis of compound **1-Gd**, HRMS (ESI+) calc. for [M+H]^+^ (C_26_H_40_N_6_O_10_Gd^+^): 754.2049, found: 754.2041.


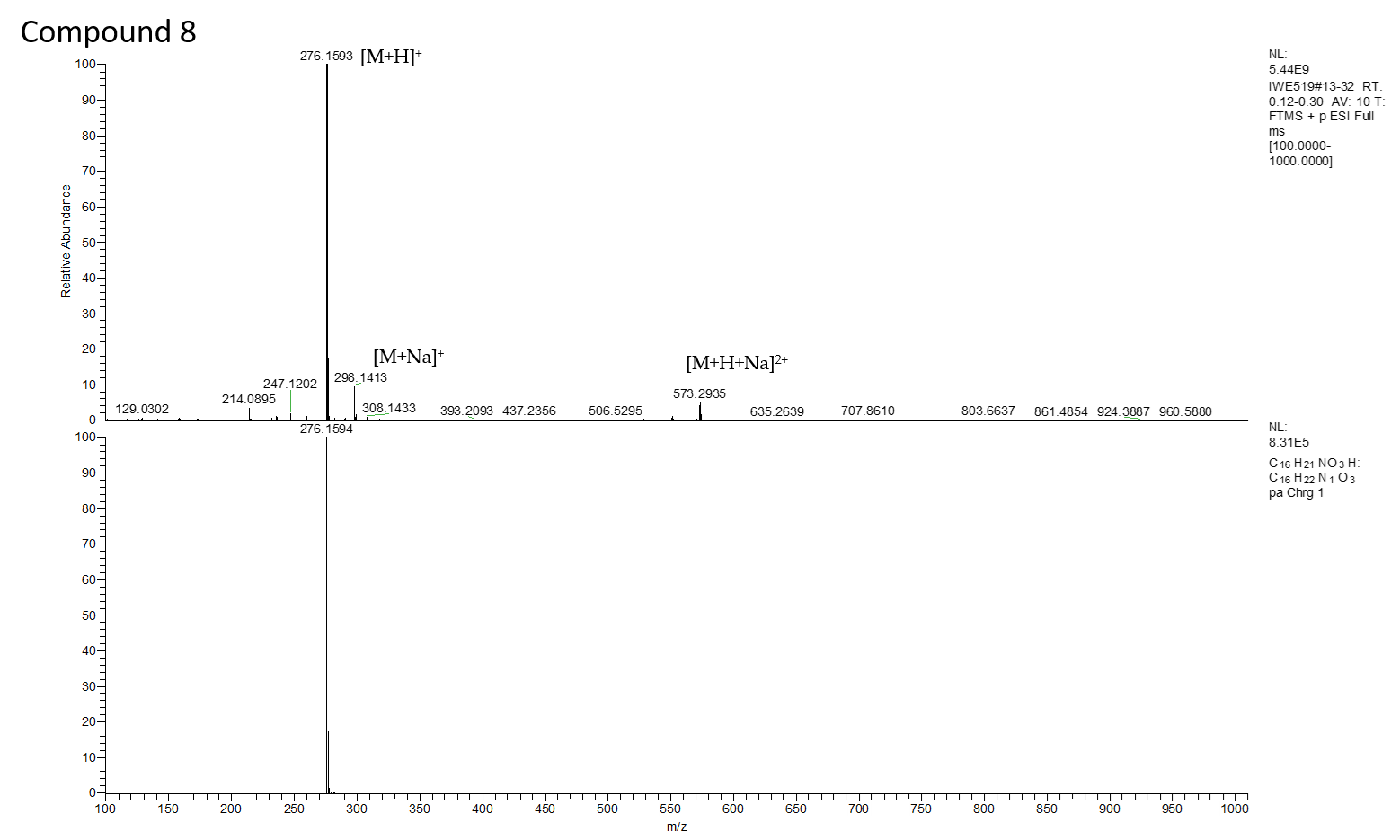


**Measured**

**Calculated**


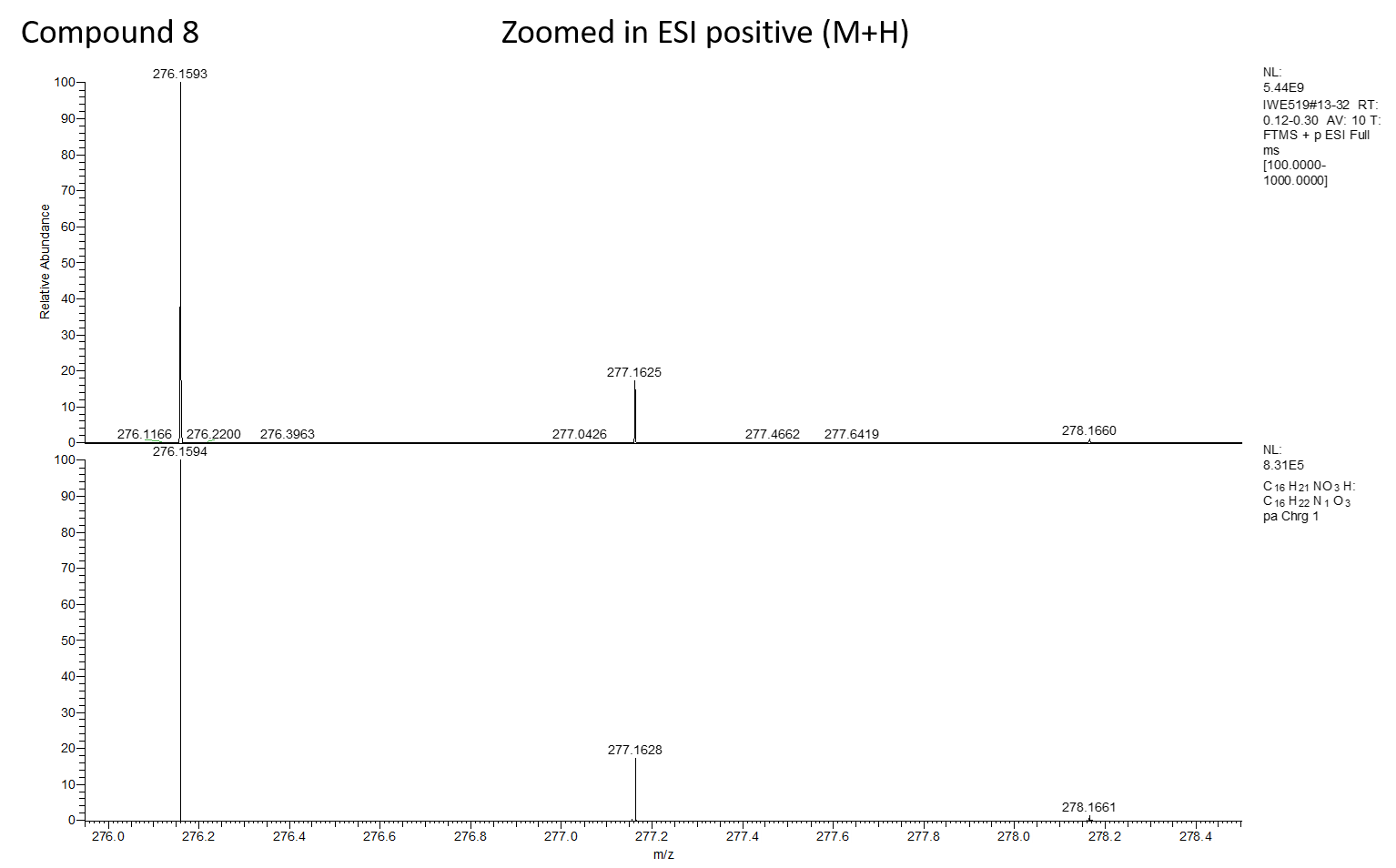


**Measured**

**Calculated**

**Figure S38**, HRMS analysis of compound **8**, HRMS (ESI+): calc. for [M+H]+ (C_16_H_22_NO_3_^+^):276.1594; found: 276.1593.


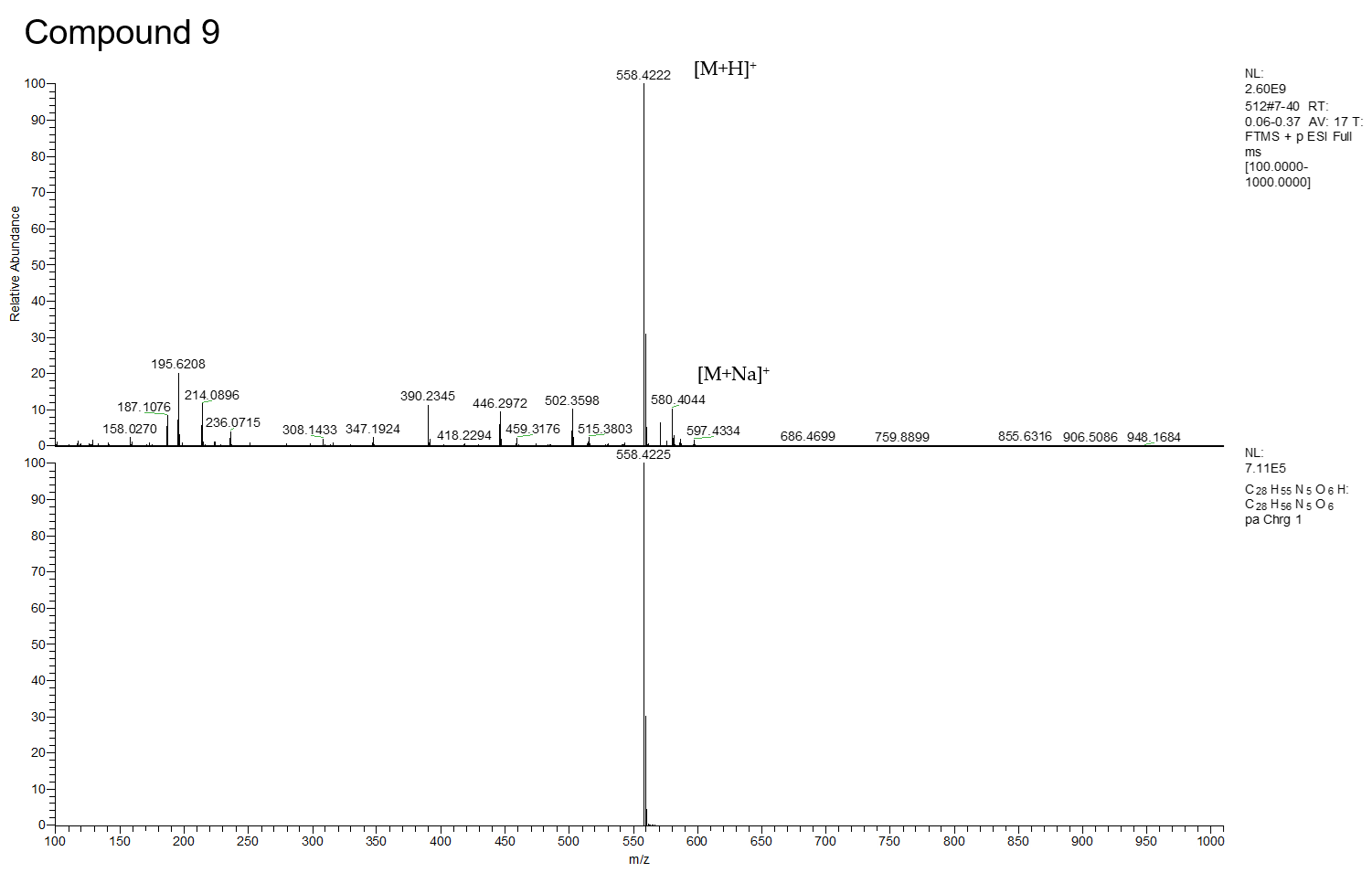


**Measured**

**Calculated**


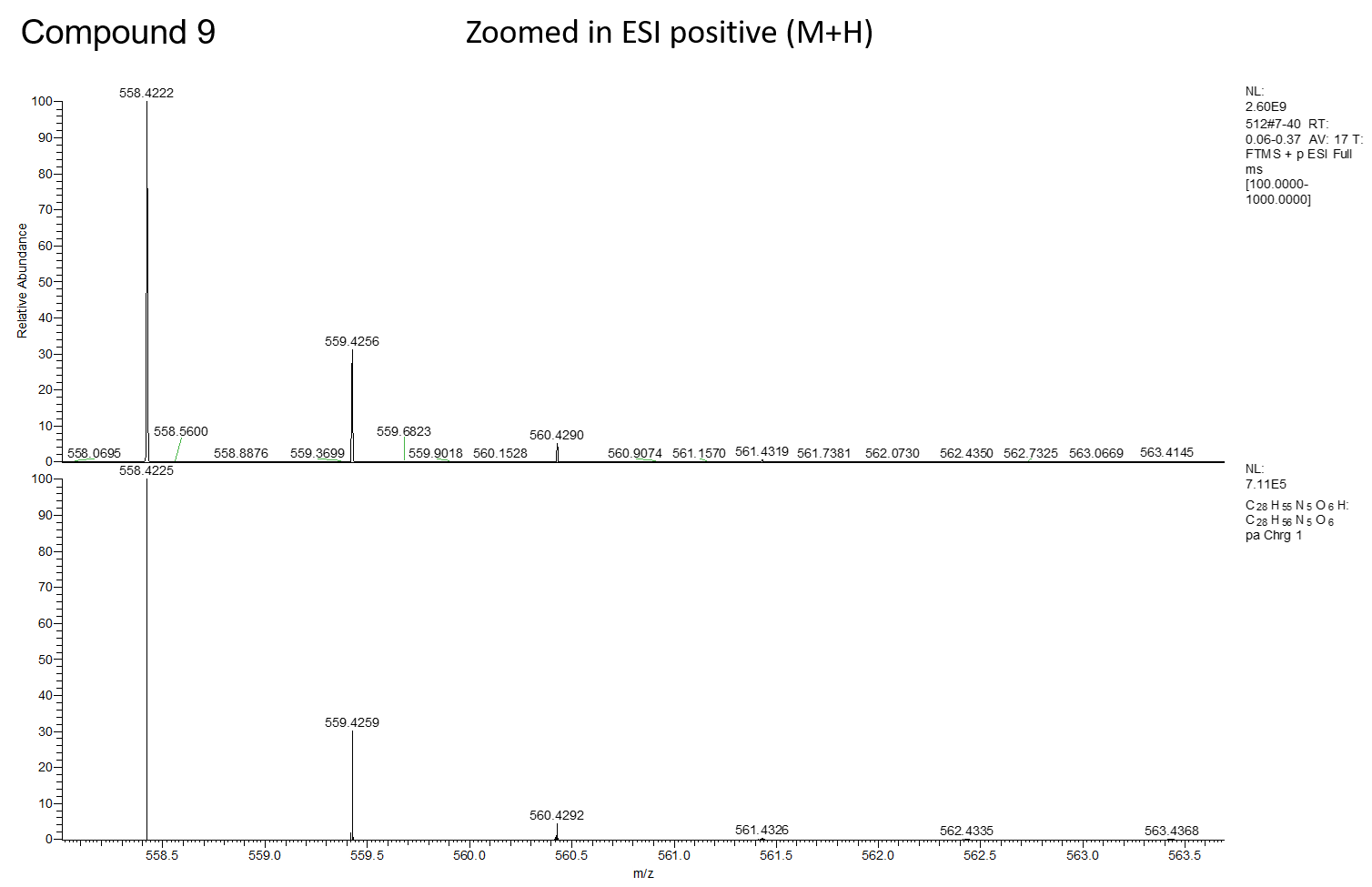


**Measured**

**Calculated**

**Figure S39**. HRMS analysis of compound **9**, HRMS (ESI+) calc. for [M+H]+ (C_28_H_56_N_5_O_6_^+^): 558.4225, found: 558.4222.


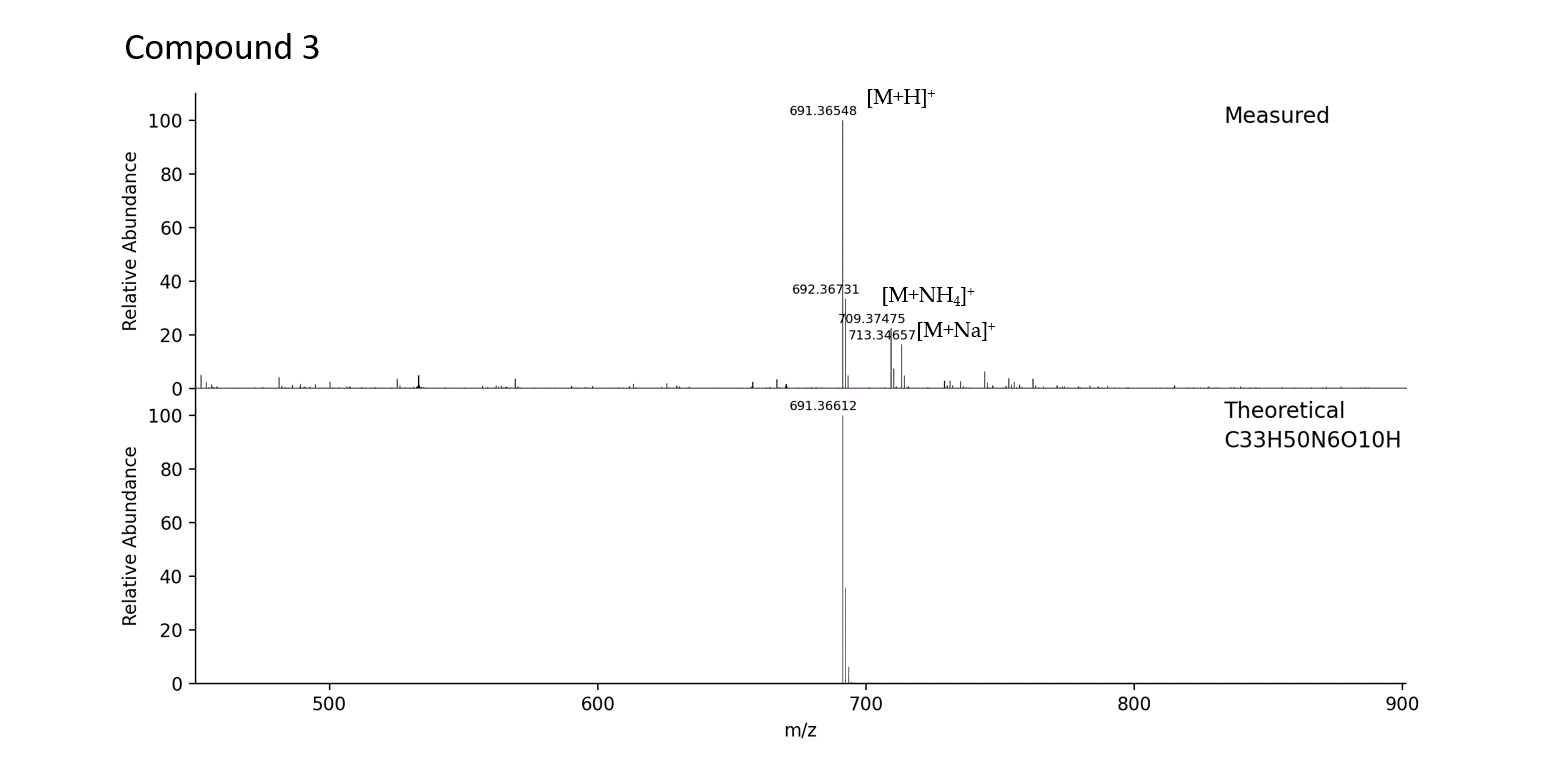


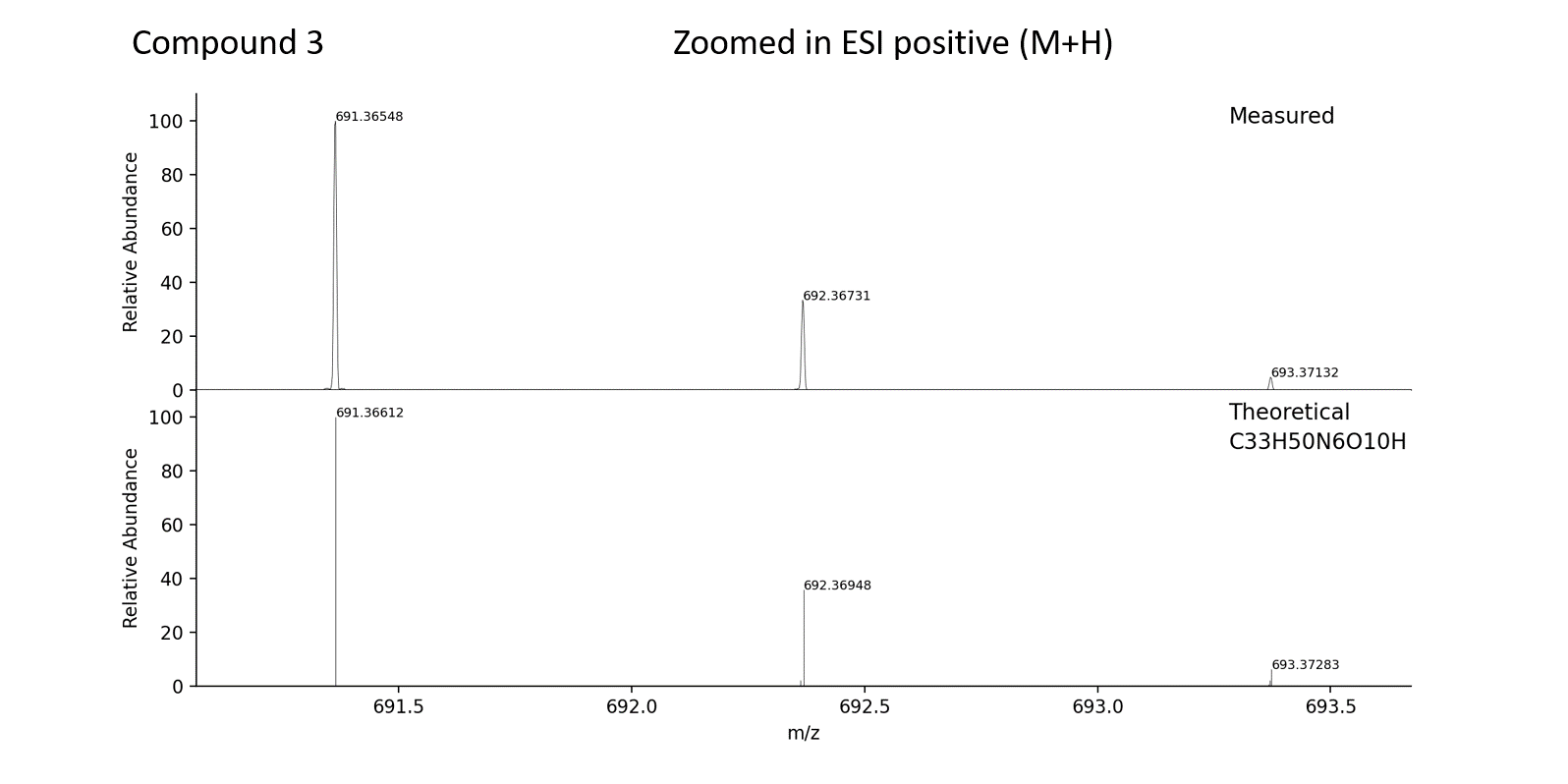


**Figure S40.** HRMS analysis of compound **3**, HRMS (ESI+) calc. for [M+H]^+^ (C_33_H_51_N_6_O_10_^+^):691.3661, found: 691.3655.


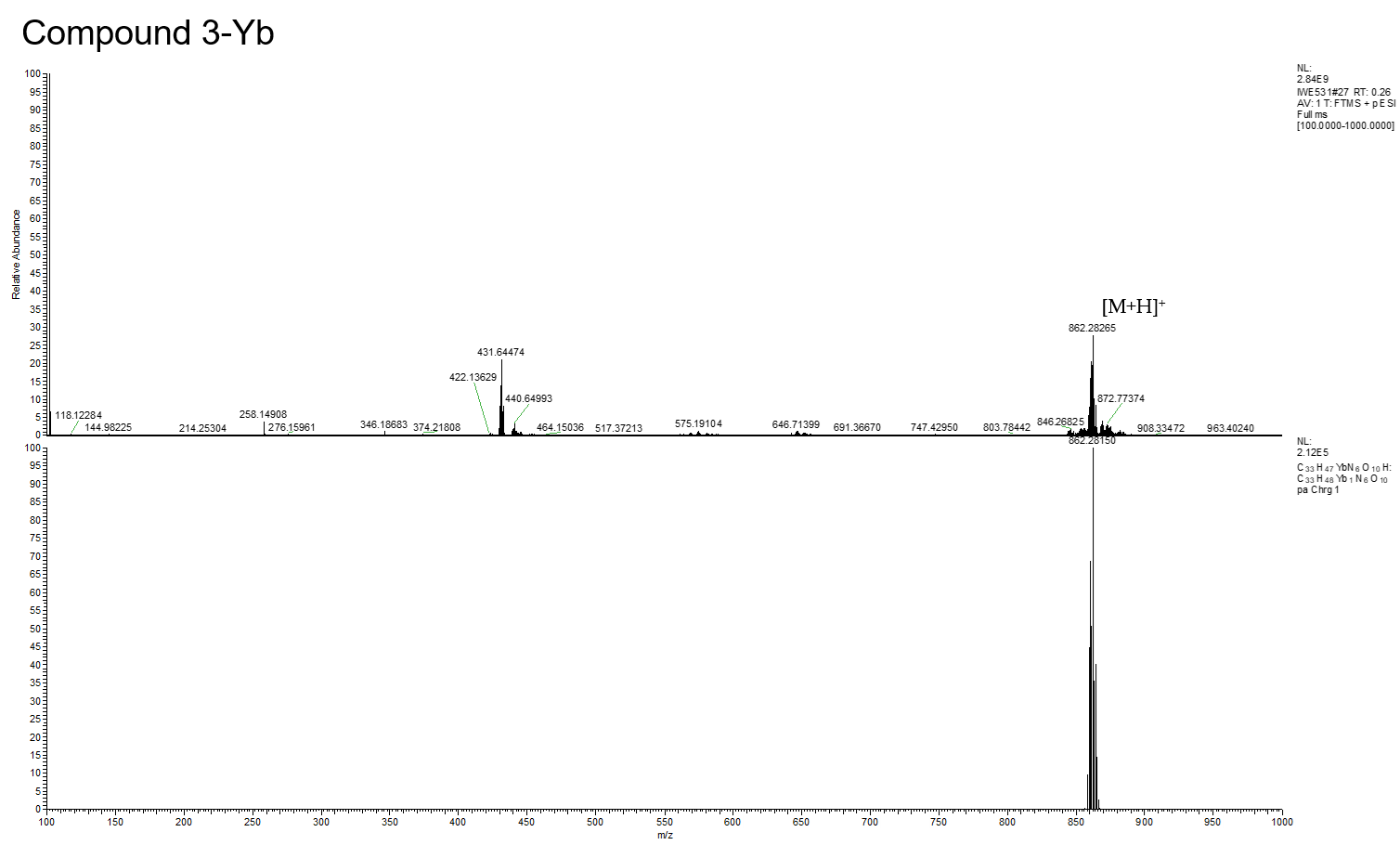


**Measured**

**Calculated**


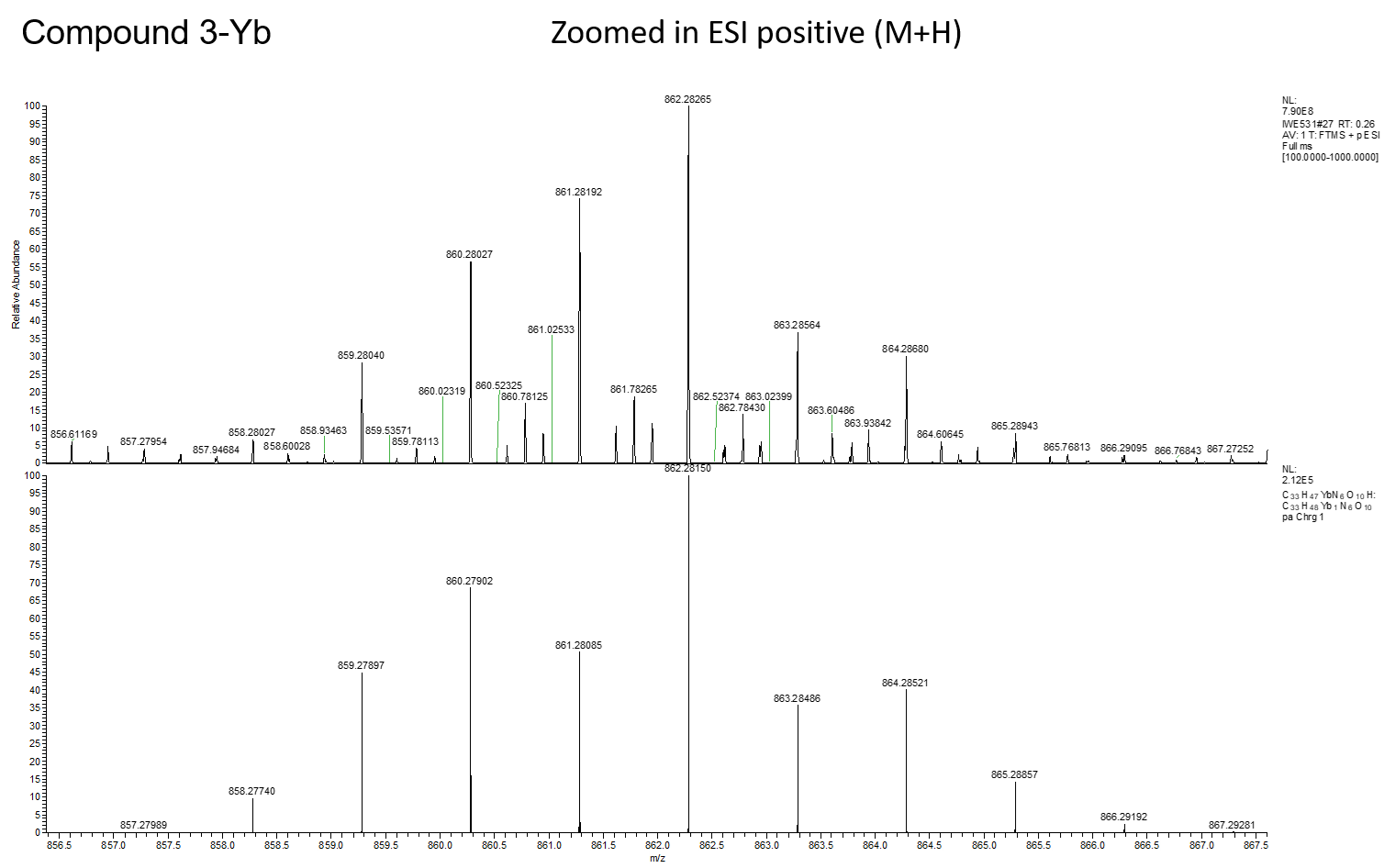


**Measured**

**Calculated**

**Figure S41**. HRMS analysis of compound **3-Yb**, HRMS (ESI+) calc. for [M+H]+ (C_33_H_46_YbN_6_O_10_^+^):862.2826, found: 860.2815.


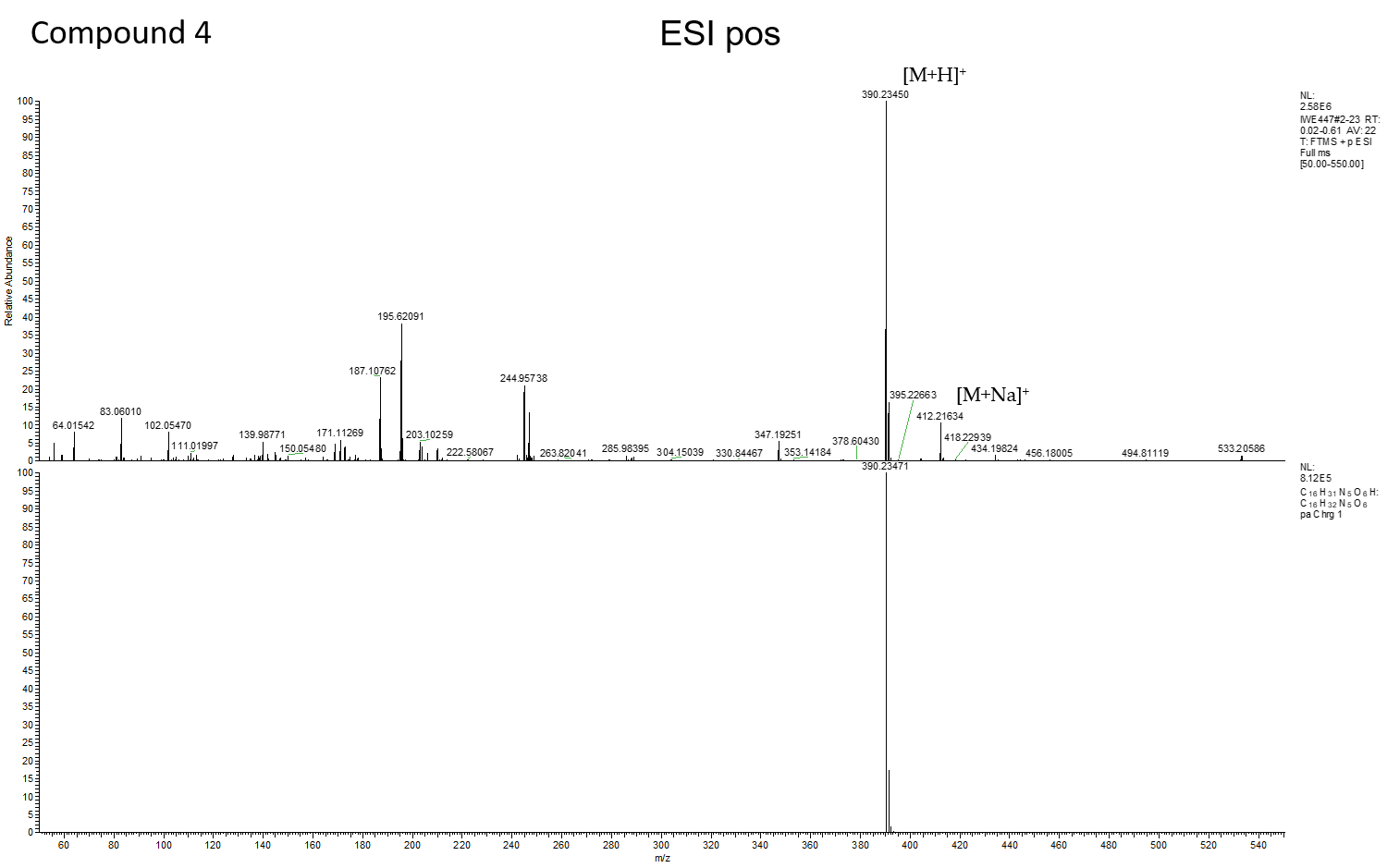


**Measured**

**Calculated**


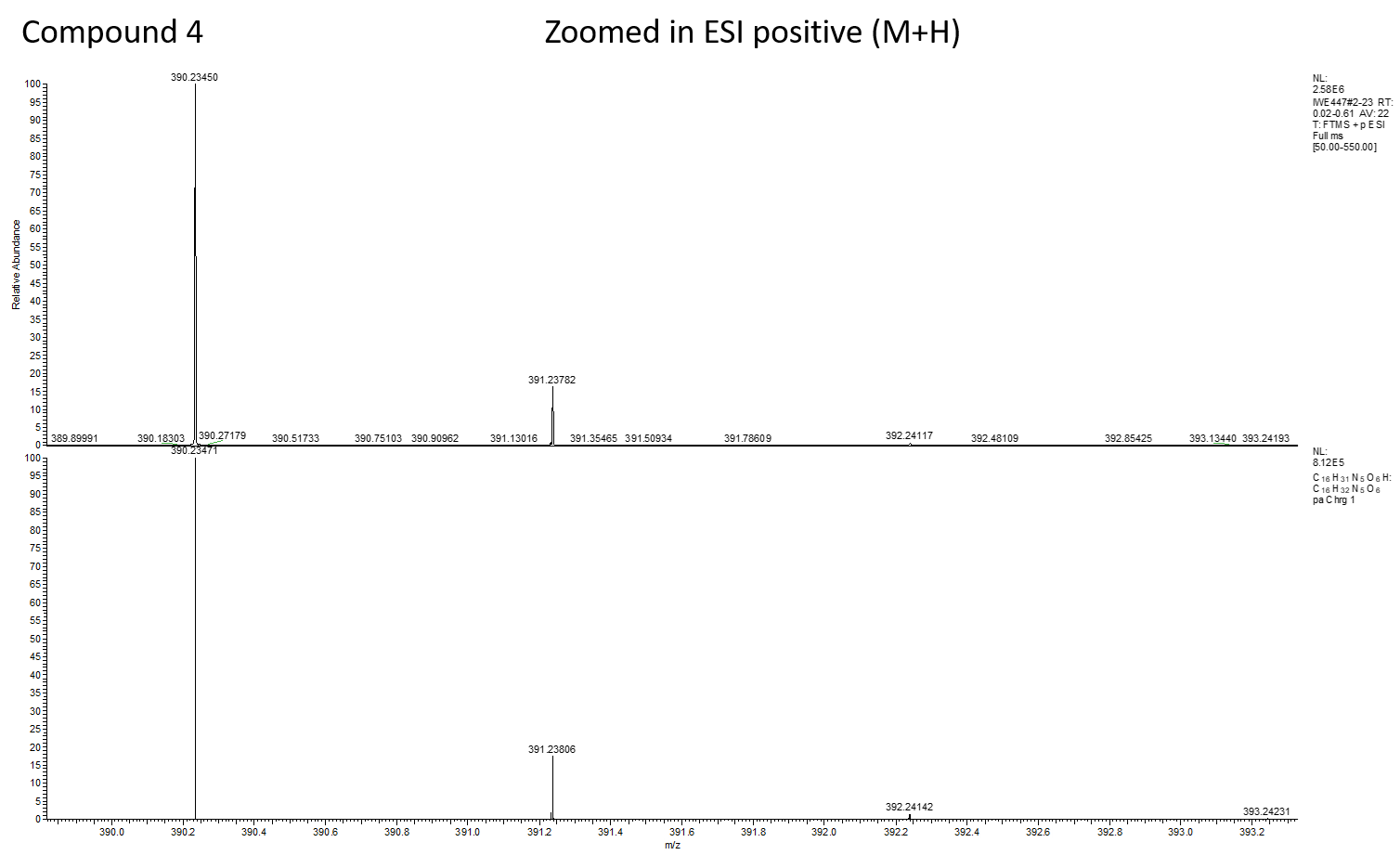


**Measured**

**Calculated**

**Figure S42.** HRSM of compound **4**, HRMS (ESI+) calc. for [M+H]^+^ (C_16_H_32_N_5_O_6_^+^): 390.2347, found: 390.2345.

**
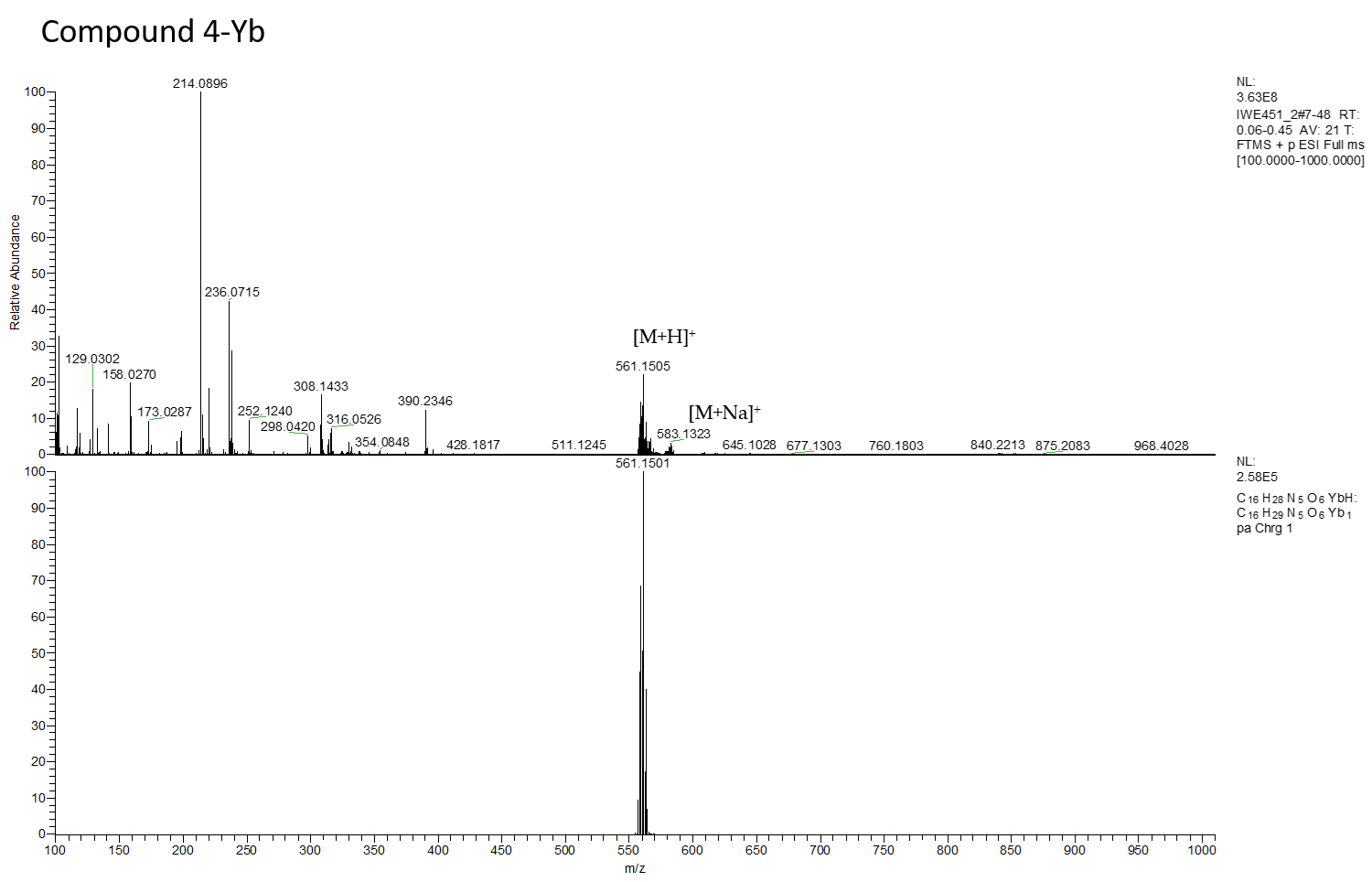
**

**Measured**

**Calculated**

**
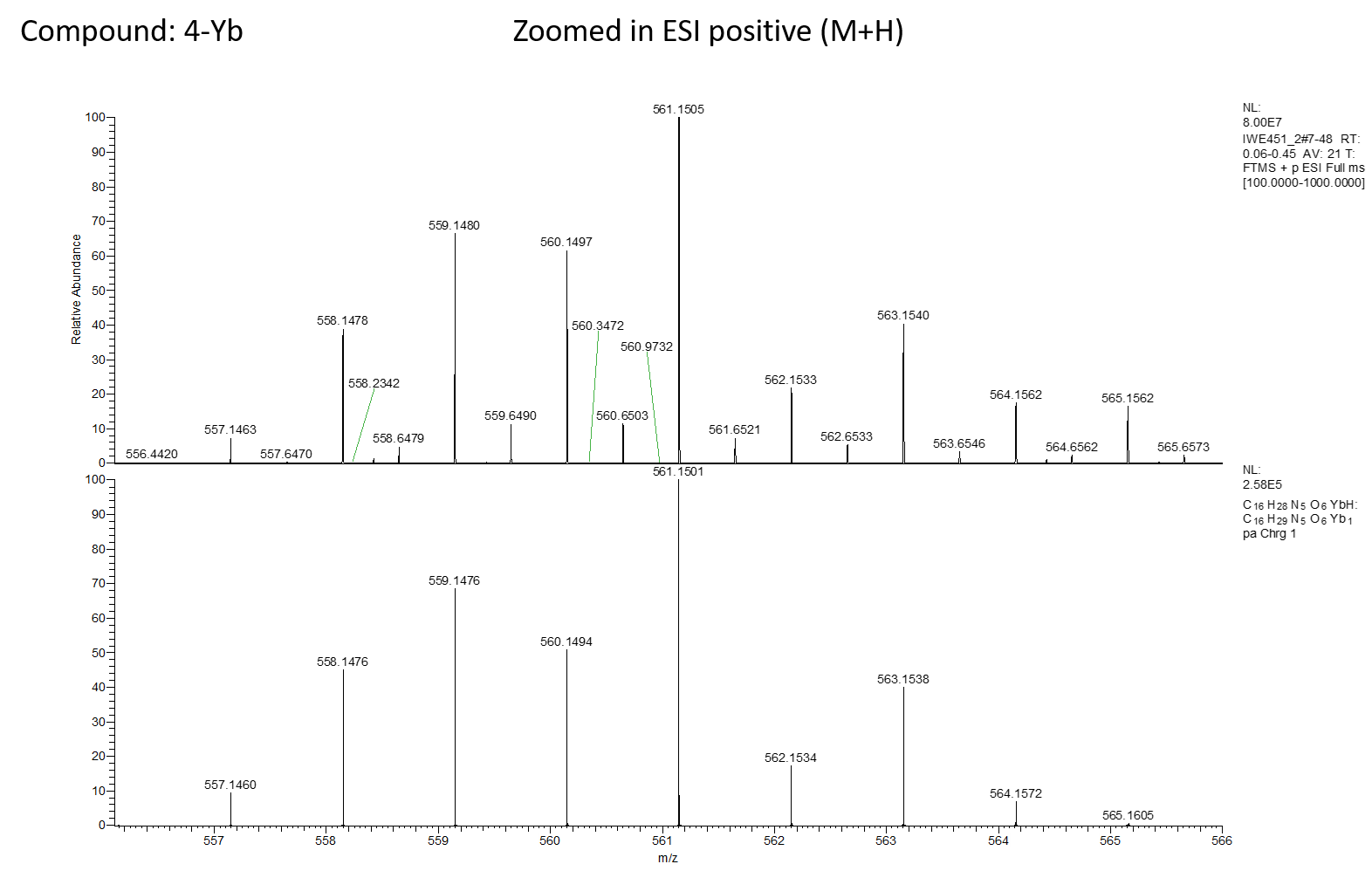
**

**Measured**

**Calculated**

**Figure S43.** HRSM of **4-Yb**, HRMS (ESI+) calc. for [M+H]^+^ (C_16_H_29_N_5_O_6_Yb^+^): 561.1500, found:561.1505.

9. IR


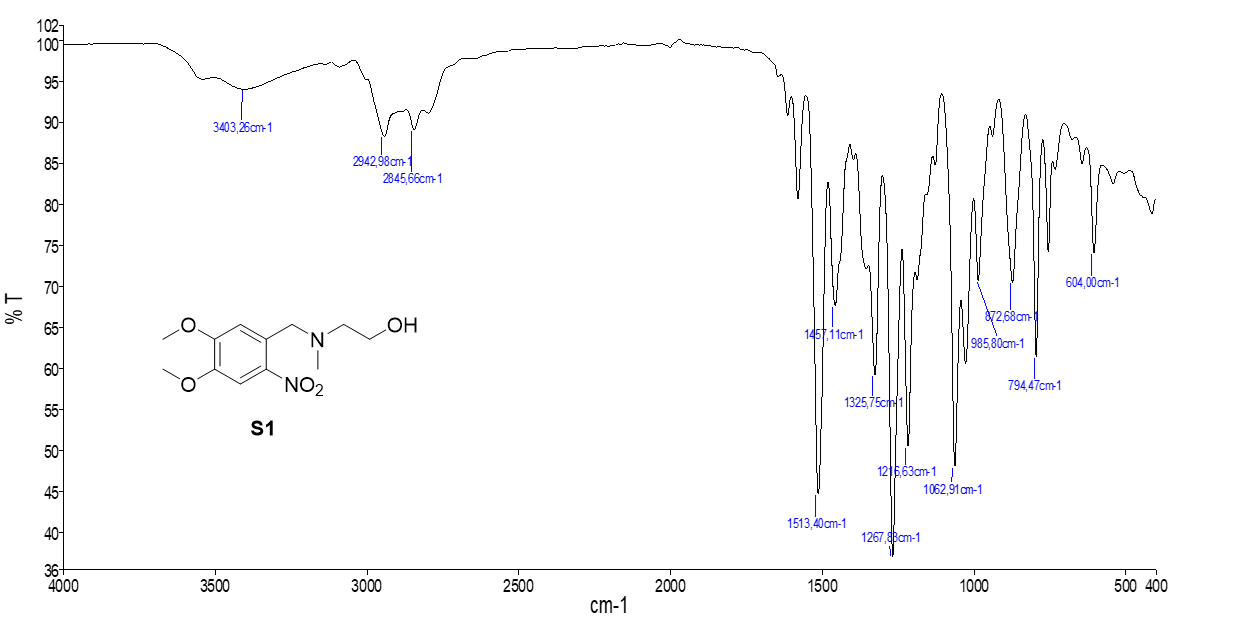


**Figure S44.** IR of 2-((4,5-dimethoxy-2-nitrobenzyl)(methyl)amino)ethan-1-ol (**S1**).


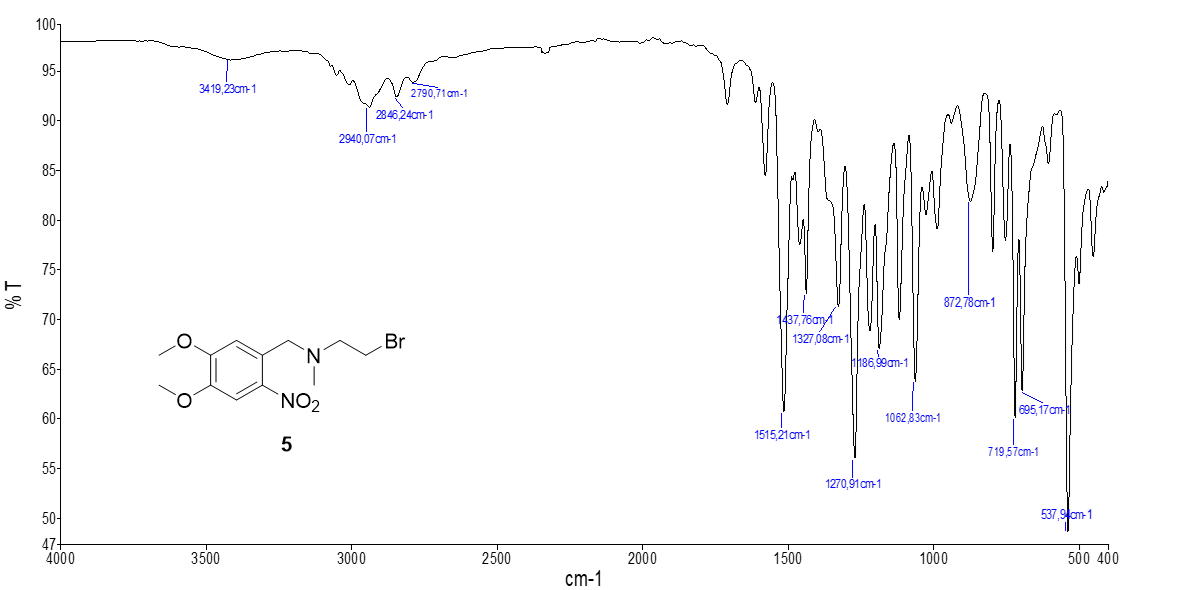


**Figure S45**. IR of 2-bromo-N-(4,5-dimethoxy-2-nitrobenzyl)-N-methylethan-1-amine (**5**).

10. UPLC-MS


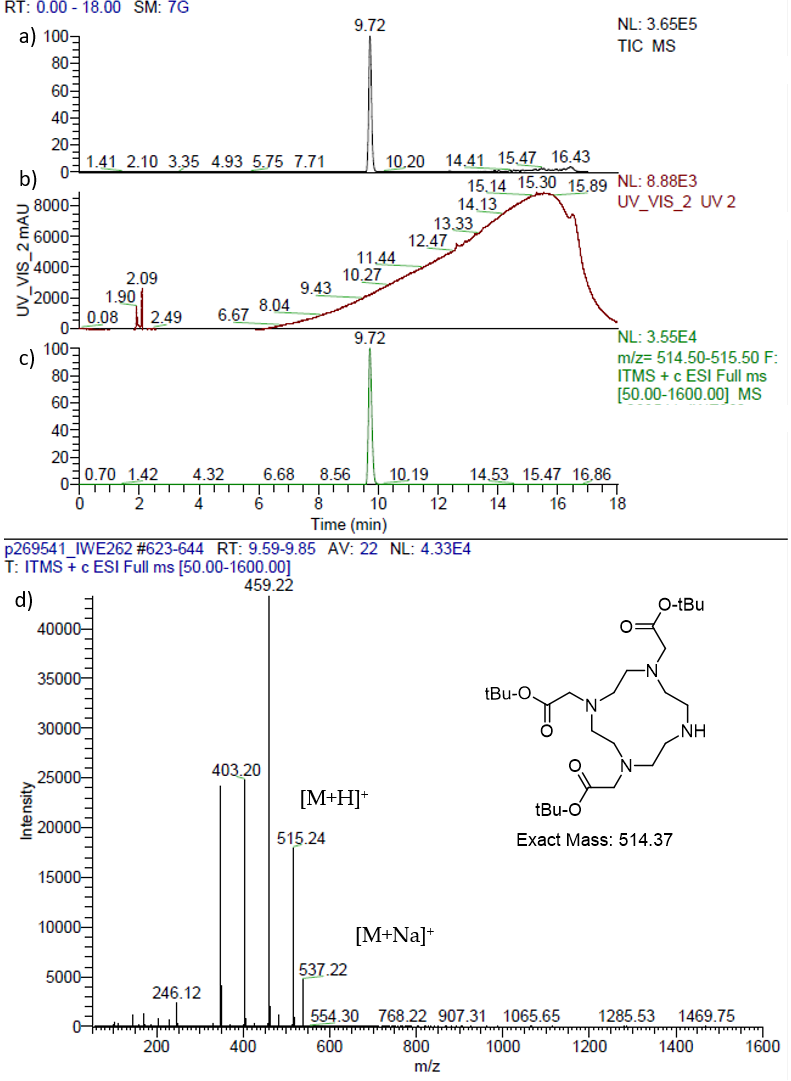


**Figure S46.** UPLC-MS analysis of compound **6** using UPLC-MS program 1 (see section 1), a) total ion current chromatogram (positive mode), b) UV-vis at 254 nm chromatogram, c) 514-515 mass trace, d) mass spectrum of the peak at Rt = 9.72 min.


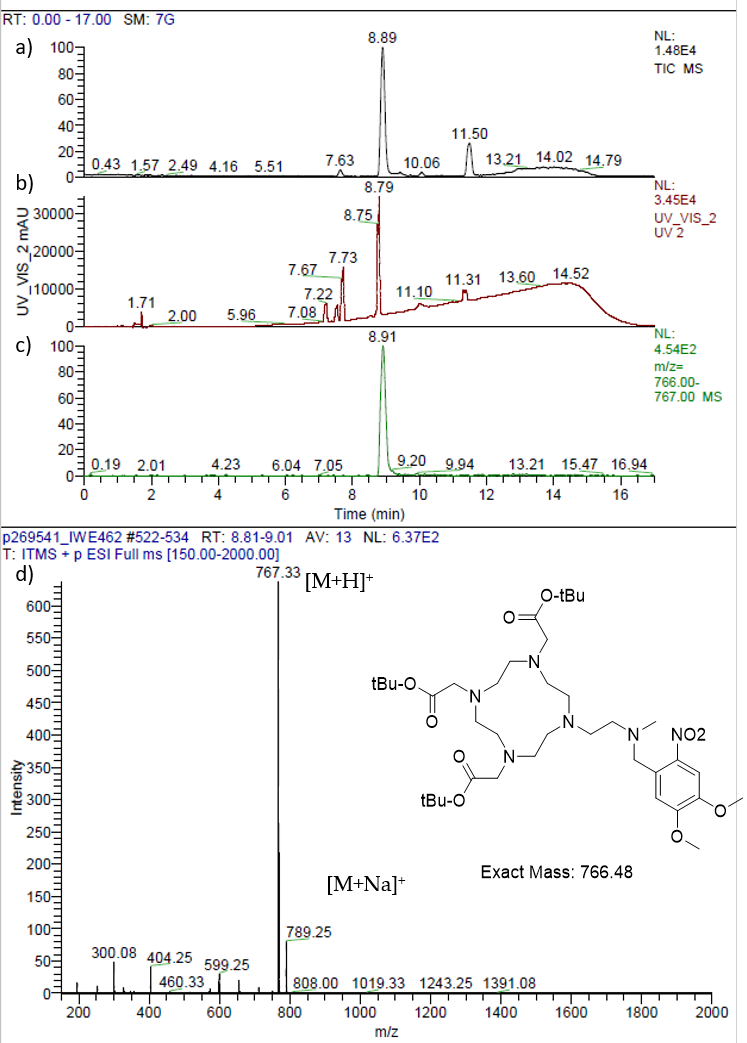


**Figure S47**, UPLC-MS analysis of compound **7** using UPLC-MS program 1 (see section 1), a) total ion current chromatogram (positive mode), b) UV-vis at 254 nm chromatogram, c) 766-767 mass trace, d) mass spectrum of the peak at Rt = 8.89 min.


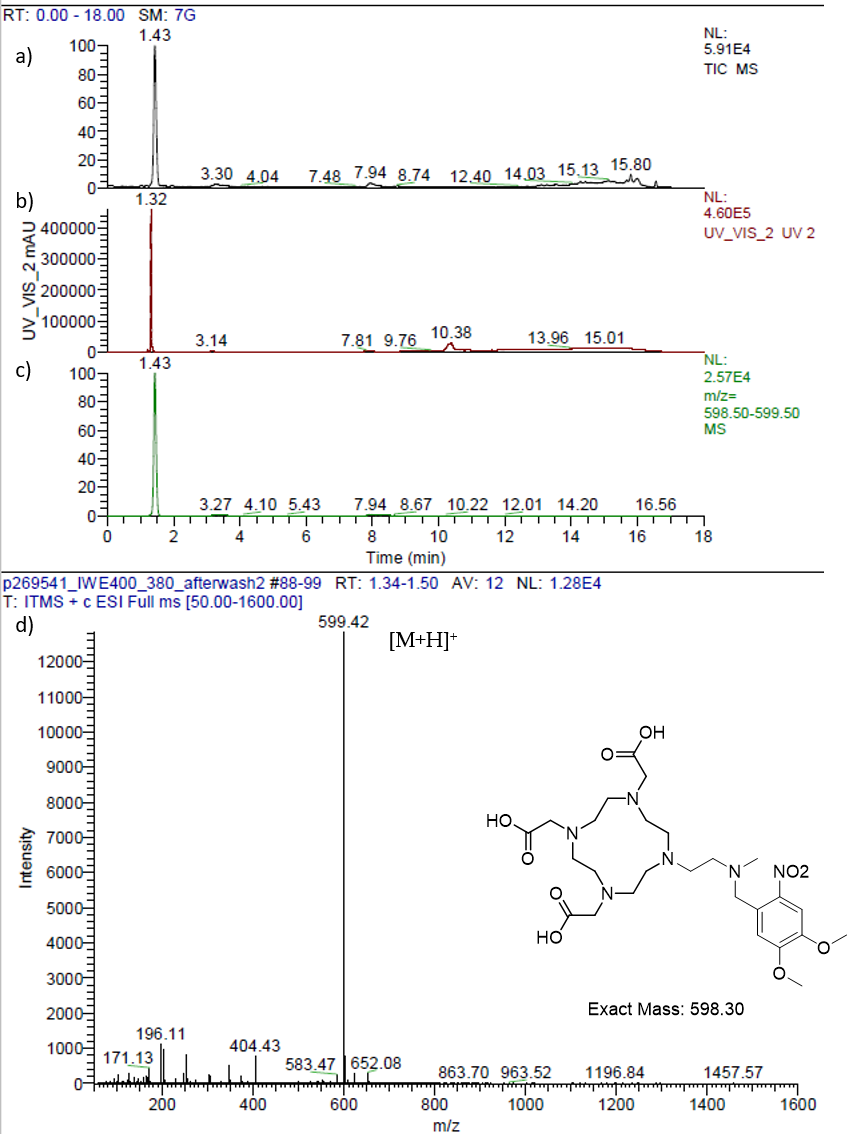


**Figure S48**. UPLC-MS analysis of compound **1** using UPLC-MS program 1 (see section 1), a) total ion current chromatogram (positive mode), b) UV-vis at 254 nm chromatogram, c) 598-599 mass trace, d) mass spectrum of the peak at Rt = 1.43 min.


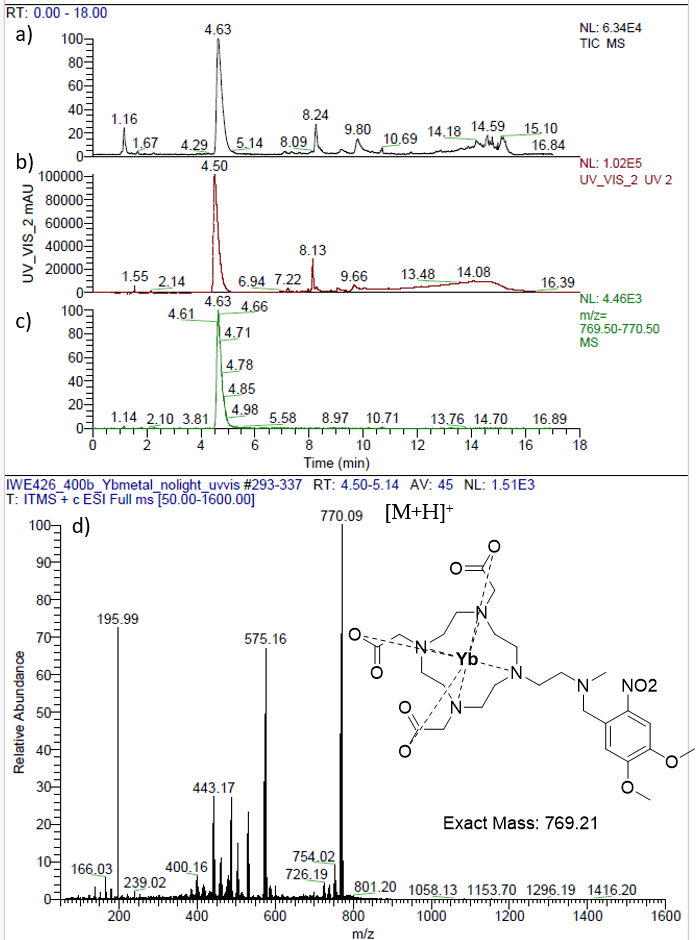


**Figure S49**. UPLC-MS analysis of compound **1-Yb** using UPLC-MS program 1 (see section 1), a) total ion current chromatogram (positive mode), b) UV-vis at 254 nm chromatogram, c) 769-770 mass trace, d) mass spectrum of the peak at Rt = 6.94 min.


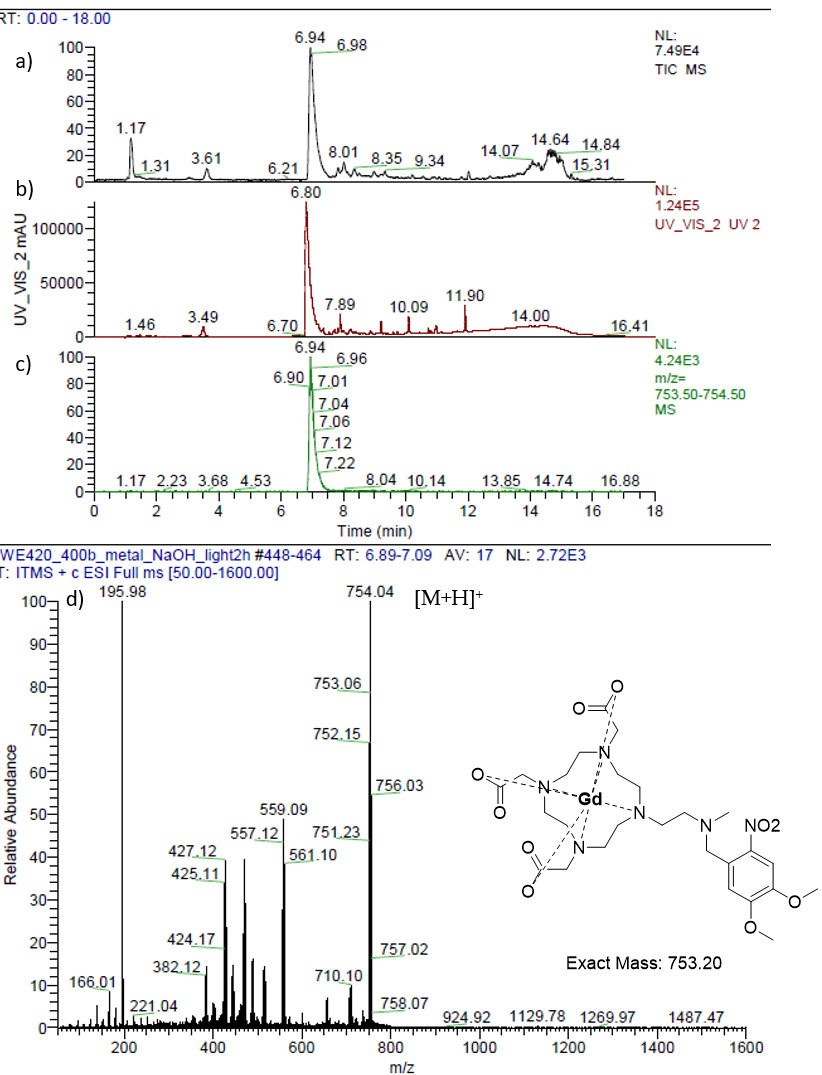


**Figure S50**. UPLC-MS analysis of compound **1-Gd** using UPLC-MS program 1 (see section 1), a) total ion current chromatogram (positive mode), b) UV-vis at 254 nm chromatogram, c) 753-754 mass trace, d) mass spectrum of the peak at Rt = 6.94 min.

*
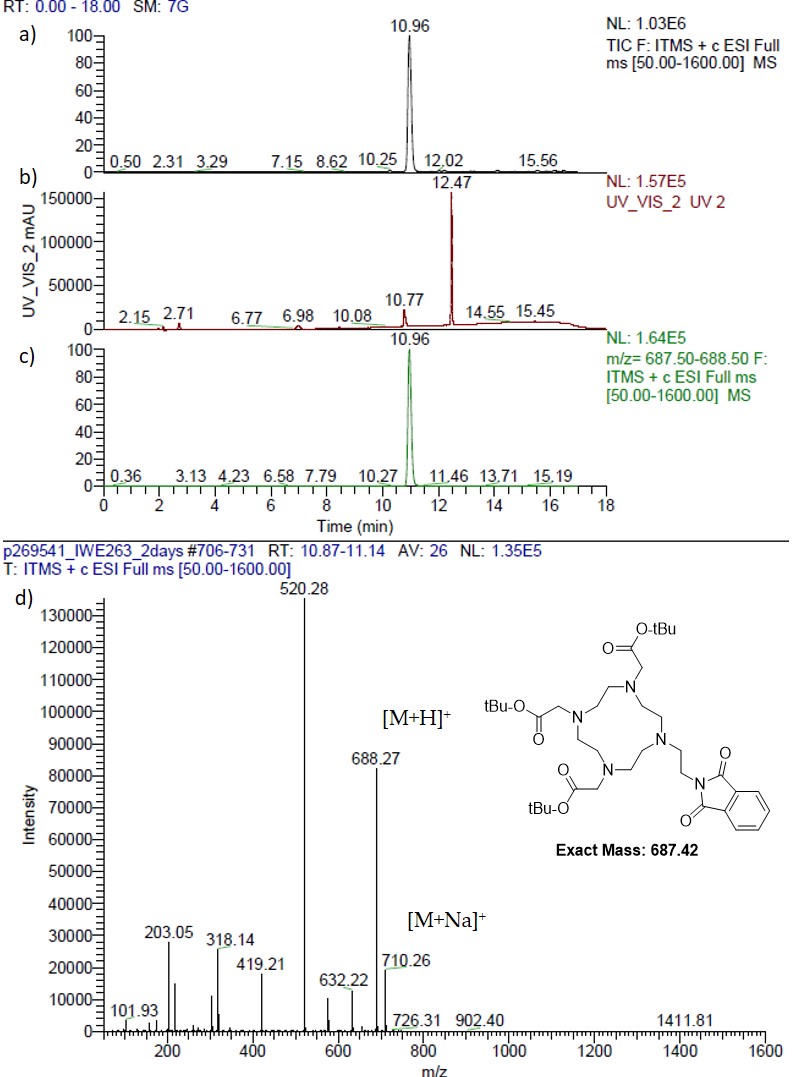
*

**Figure S51**. UPLC-MS analysis of compound **11** using UPLC-MS program 1 (see section 1), a) total ion current chromatogram, b) UV-vis at 254 nm chromatogram, c) 687-688 mass trace, d) mass spectrum of the peak at Rt = 10.96 min.


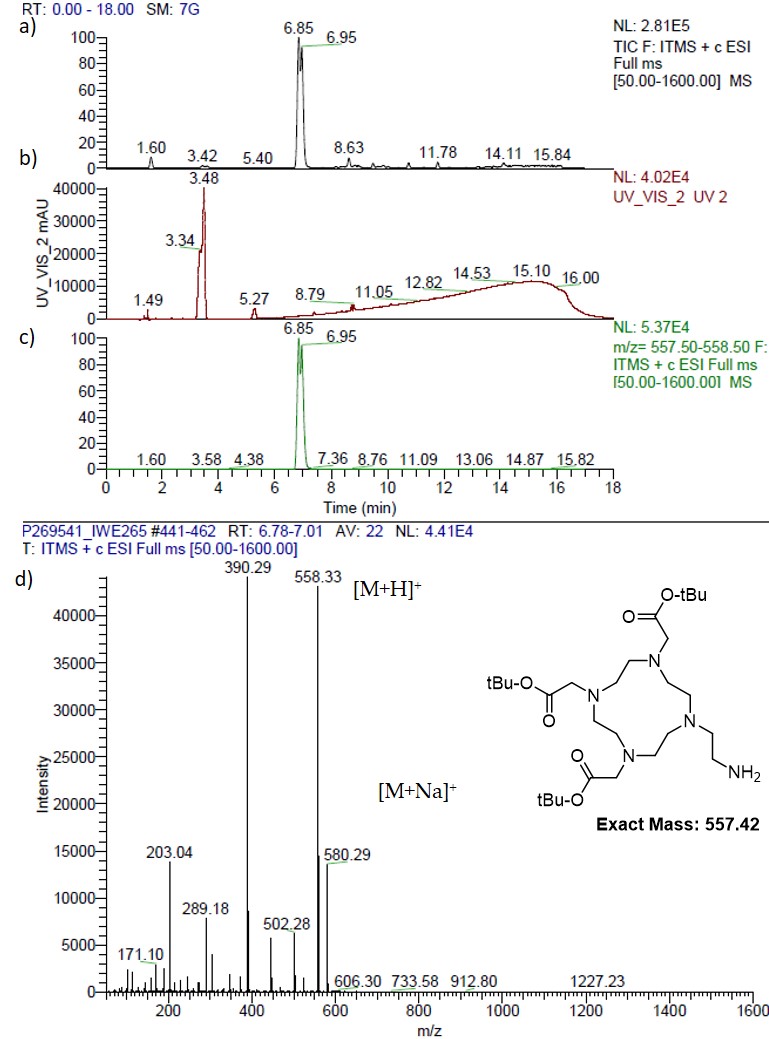


**Figure S52.** UPLC-MS analysis of compound **9** using UPLC-MS program 1 (see section 1), a) total ion current chromatogram, b) UV-vis at 254 nm chromatogram, c) 557-558 mass trace, d) mass spectrum of the peak at Rt = 6.85 min.


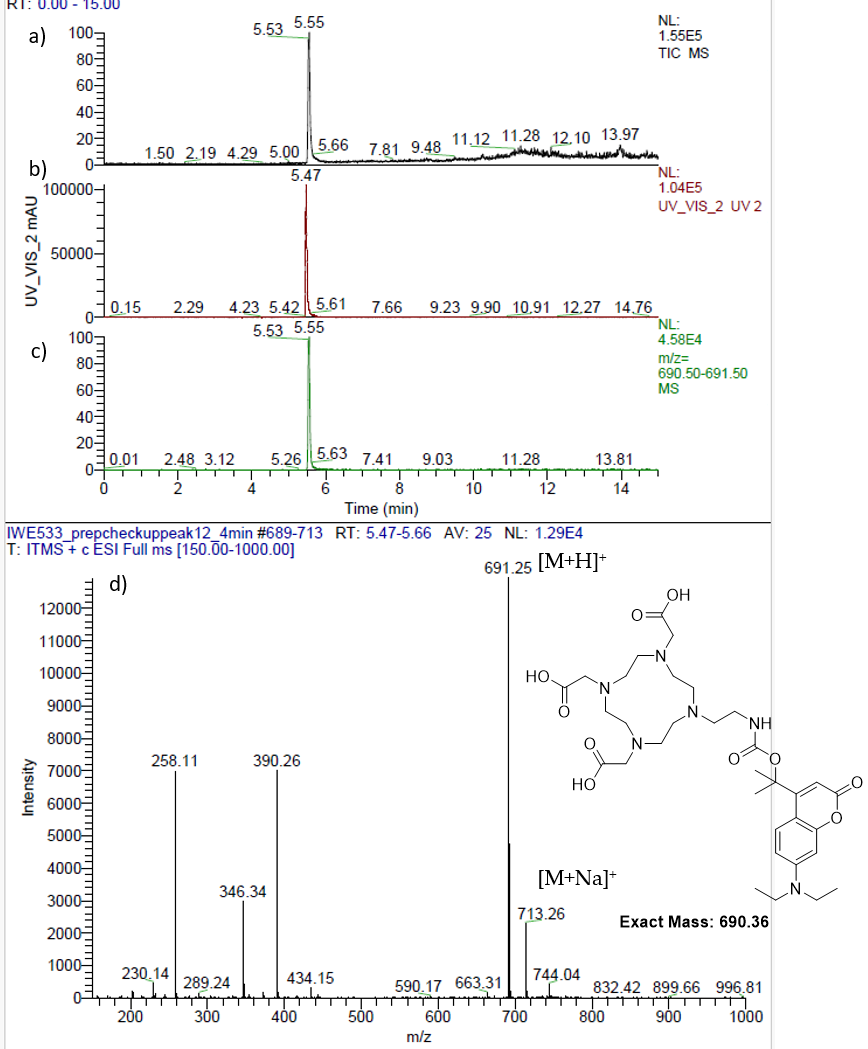


**Figure S53.** UPLC-MS analysis of compound **3** using UPLC-MS program 1 (see section 1), a) total ion current chromatogram (positive mode), b) UV-Vis at 254 nm chromatogram, c) 690-691 mass trace, d) mass spectrum of the peak at Rt = 5.55 min.


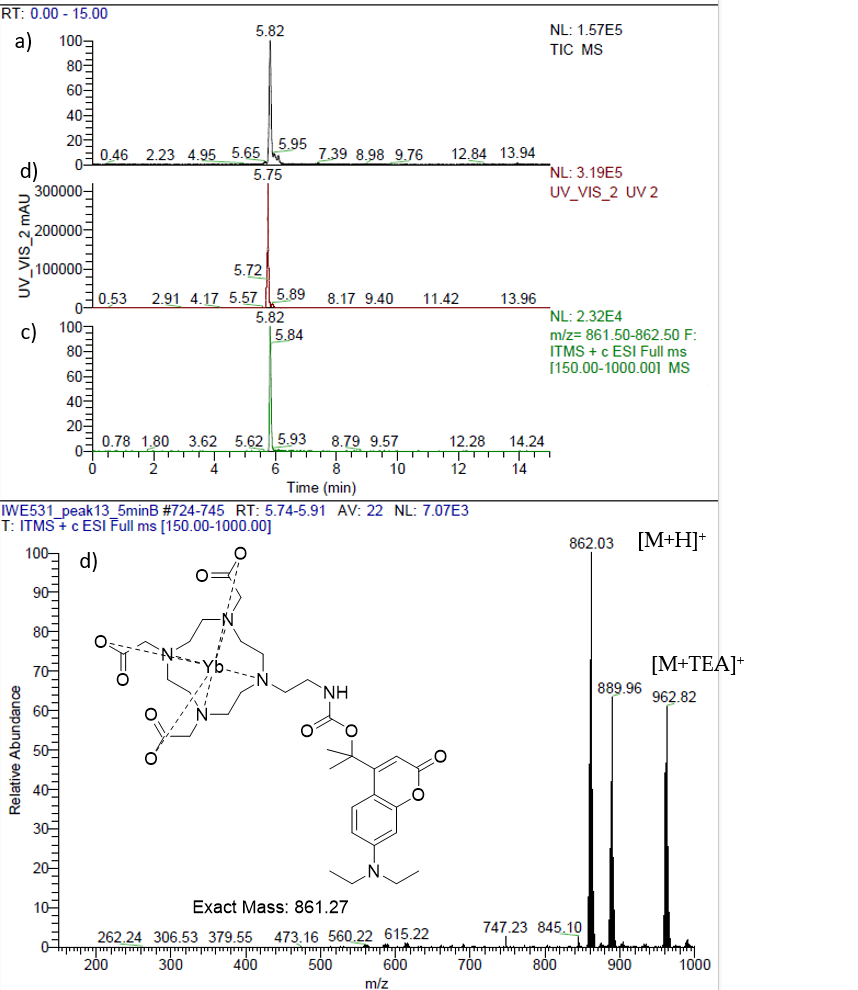


**Figure S54**. UPLC-MS analysis of compound **3-Yb** after PREP HPLC purification, using UPLC-MS program 1 (see section 1), a) total ion current chromatogram (positive mode), b) UV-Vis at 254 nm chromatogram, c) 861-862 mass trace, d) mass spectrum of the peak at Rt = 5.82 min.

**
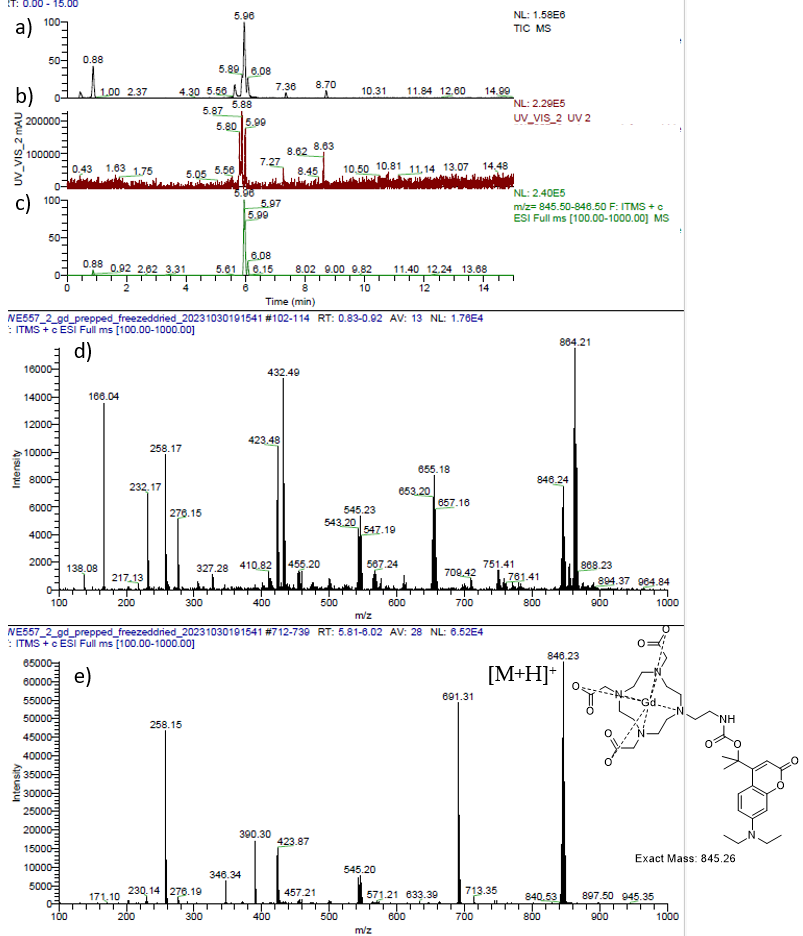
**

**Figure S55.** UPLC-MS analysis of compound **3-Gd** after PREP HPLC purification, using UPLC-MS program 1 (see section 1), a) total ion current chromatogram (positive mode), b) UV-vis at 254 nm chromatogram, c) 514-515 mass trace, d) mass spectrum of the peak at Rt = 9.72 min


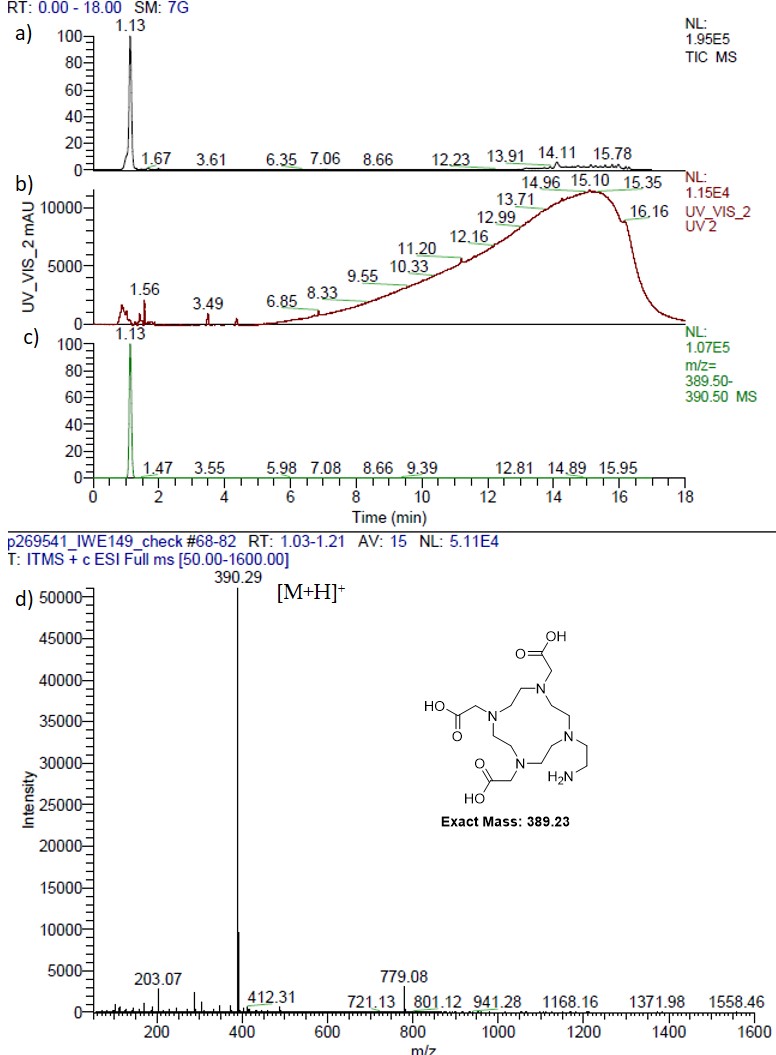


**Figure S56**. UPLC-MS analysis of compound **4** using UPLC program 1 (see section 1), (a) total ion current chromatogram, (b) UV-vis at 254 nm chromatogram), (c) 389-390 mass trace, (d) mass spectrum of the peak at R_t_ = 1.13 min.


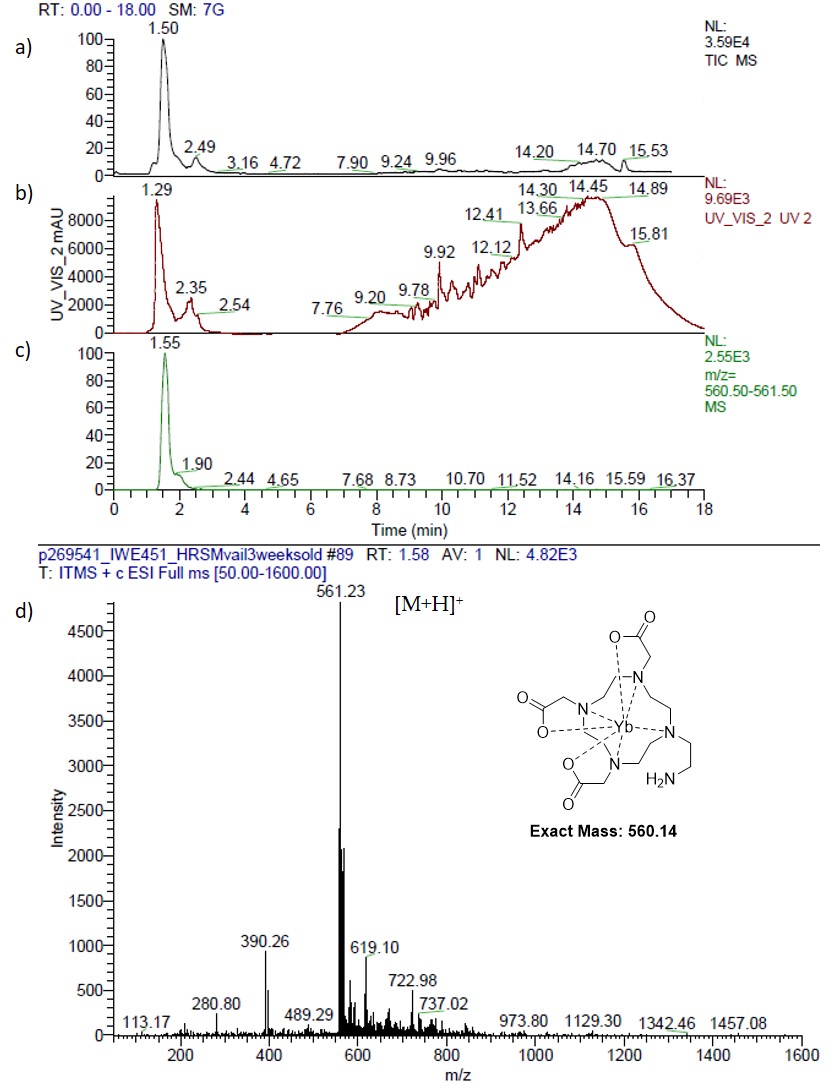


**Figure S****57**. UPLC-MS analysis of compound **4-Yb** using UPLC program 1 (see section 1), (a) total ion current chromatogram, (b) UV-vis at 254 nm chromatogram), (c) 560-561 mass trace, (d) mass spectrum of the peak at R_t_ = 1.50 min.

**10. Stability tests.**

Initial stability test of compounds **1-Gd**, **1-Yb, 3-Yb** were performed in human plasma-like medium (HPLM), and the samples were followed by UPLC-MS. For this analysis, three samples of 1 mM, in 0.6 mL HPLM were prepared of each contrast agent, then 0.1 mL was taken out diluted with 0.1 mL water and injected in the UPLC-MS before and after 24 h at 37 °C. Analyzed with UPLC-MS program 3. Results are shown in table S10-12 and visualized in Figure S58-59.

Table S10. UPLC-MS analysis of **1-Gd** in HPLM. Peak Areas of **1**-**Gd** for time point t = 0 and t = 24 h at 37 °C.

| Measurement | t0 | t24 |
| --- | --- | --- |
| 1 | 48056512.00 | 48550740.00 |
| 2 | 48228232.00 | 48526804.00 |
| 3 | 48337728.00 | 48404352.00 |
| Average | 48207490.67 | 48493965.33 |
| Standard deviation | 141750.7032 | 78524.806 |

| Measurement | t0 | t24 |
| --- | --- | --- |
| 1 | 36649832.00 | 36751096.00 |
| 2 | 37617940.00 | 36551740.00 |
| 3 | 37655264.00 | 37296228.00 |
| Average | 37307678.67 | 36866354.67 |
| Standard deviation | 570017.50 | 385394.62 |

Table S11. UPLC-MS analysis of **1-Yb** in HPLM. Peak Areas of **1**-**Yb** for time point t = 0 and t = 24 h at 37 °C.

Table S12. UPLC-MS analysis of **3-Yb** in HPLM. Peak Areas of **3-Yb** for time point t = 0 and t = 24 h at 37 °C. ND = below the detection limit.

| Measurement | t0 | t24 | Peak of with m/z = 881 at t = 24h |
| --- | --- | --- | --- |
| 1 | 1970396 | ND | 1701016 |
| 2 | 1755245 | ND | 1689992 |
| 3 | 1735949 | ND | 1488210 |
| Average | 1820530 | ND | 1626406 |
| Standard deviation | 130145.9 | ND | 119807.9 |

**Figure S58.** UPLC-MS analysis of **1-Gd** (1.0 mM) in HPLM at 37 °C followed over 24 h. Error bars represent the standard deviation.

**Figure S59.** UPLC-MS analysis of **1-Yb** (1.0 mM) in HPLM at 37 °C followed over 24 h. Error bars represent the standard deviation.


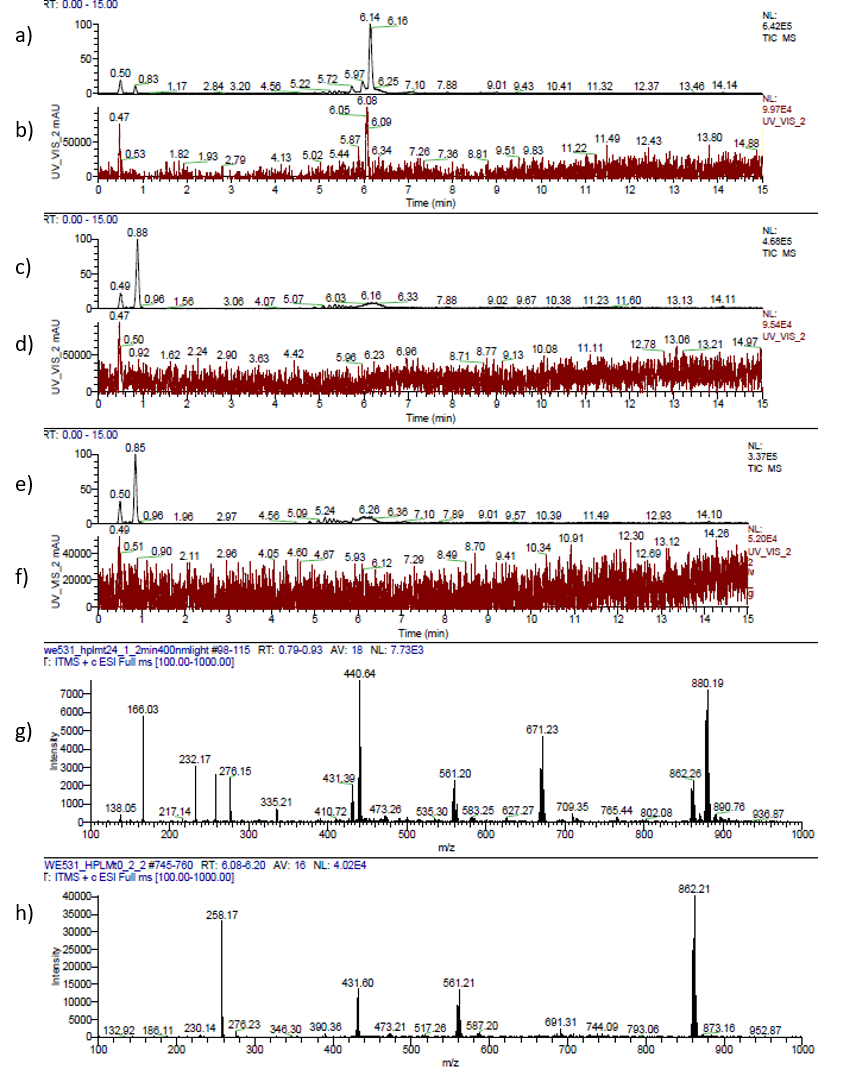


**Figure S60.** UPLC-MS analysis of **3-Yb** (1.0 mM) is shown in HPLM before and after incubation at 37 °C, after 24h an attempt of irradiation with 400 nm light was made (10 min of irradiation). We show serval parameters: a) total ion current chromatogram (positive mode) 0 min incubation, b) UV-vis at 390 nm chromatogram, 0 min incubation, c) total ion current chromatogram (positive mode) 24h incubation, d) UV-vis at 390 nm chromatogram 24h incubation, e) total ion current chromatogram (positive mode) 2 min of irradiation 400 nm after 24h incubation, d) UV-vis at 390 nm chromatogram, 2 min of irradiation 400 nm after 24h incubation, g) mass spectrum of the peak at Rt = 0.88 min, h) mass spectrum of the peak at Rt = 6.14 min.


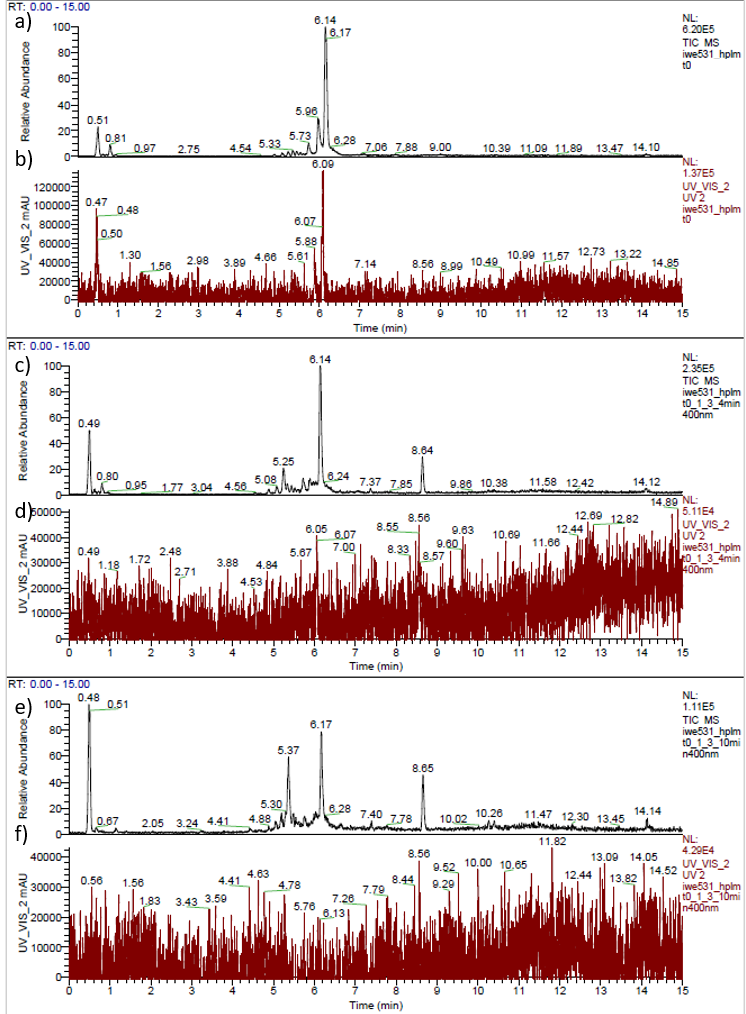


**Figure S61.** UPLC-MS analysis of **3-Yb** (1.0 mM) in HPLM at 37 °C, irradiated with 400 nm light Following parameters are shown: a) total ion current chromatogram (positive mode) 0 min of irradiation, b) UV-vis at 390 nm chromatogram, 0 min of irradiation, c) total ion current chromatogram (positive mode) 4 min of irradiation 400 nm, d) UV-vis at 390 nm chromatogram, 4 min of irradiation 400 nm, e) total ion current chromatogram (positive mode) 10 min of irradiation 400 nm, d) UV-vis at 390 nm chromatogram, 10 min of irradiation 400 nm.

**11. pH test with 4-Yb**

A 20 mM solution of **4-Yb** was prepared in milli Q and adjusted with 1 M NaOH to pH 6 or 7. Afterwards the Z-spectra were recorded on a Varian Oxford AS 500 MHz (B0 = 11.7T), using 5 mm sample tubes. (see figure captions for exact conditions)


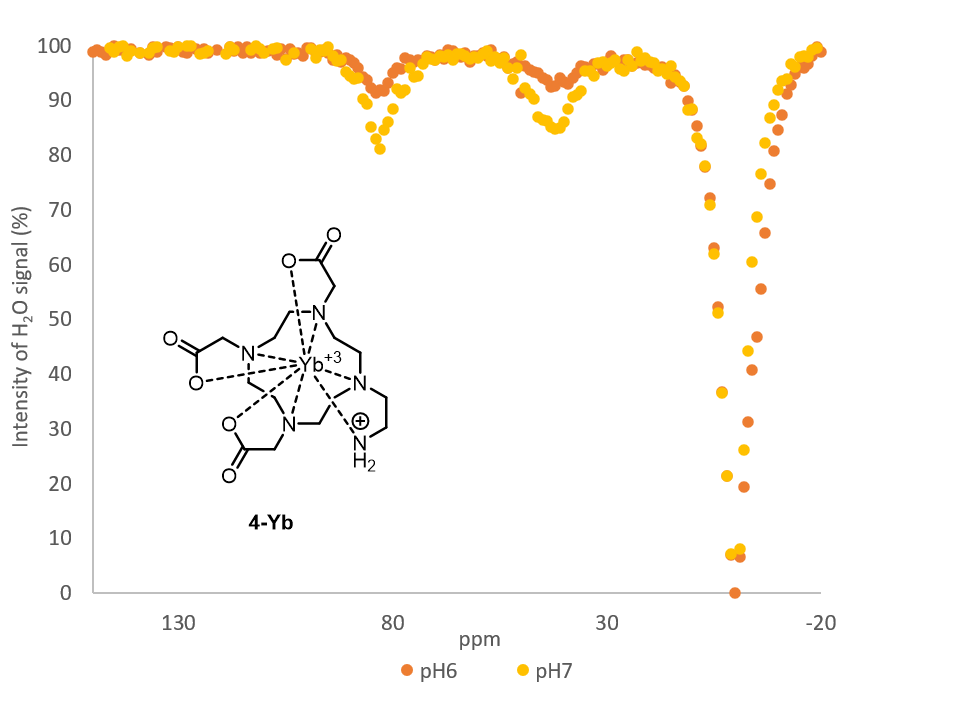


**Figure S62.** The NMR-Z profiles for **4-Yb** measured with different pH 6 and pH 7 (20 mM in water with 10% D_2_O, pH adjusted with 1 mM NaOH, B0 = 11.7 T, satpwr = 28 dB, satdly = 2 s T=37 °C)

**12. Reference**

1. X. Jiang, B. Huang, F. A. Olotu, J. Li, D. Kang, Z. Wang, E. De Clercq, M. E. S. Soliman, C. Pannecouque, X. Liu and P. Zhan, *Eur. J. Med. Chem*., **2021**, *213*, 113051.

2. H. Sajiki, K. Y. Ong, *Tetrahedron*, **1996**, *52*, 14507-14514.

3. F. Wan, M. Liu, J. Zhang, Y. Li and L. Jiang. Res. Chem. Intermed., 2015, 41, 5109–5119.

4. A. De La Reberdière, F. Lachaud, F. Chuburu, C. Cadiou and G. Lemercier, *Tetrahedron Lett.*, **2012**, *53*, 6115–6118.

5. A. Barge, G. Cravotto, E. Gianolio and F. Fedeli*, Contrast Media Mol. Imaging.*, **2006**, *1*, 184–188.

6. A. M. Schulte, G. Alachouzos, W. Szymański and B. L. Feringa, *J. Am. Chem. Soc.*, **2022**, *144*, 12421–12430.

7. I. M. Welleman, F. Reeβing, H. H. Boersma, R. A. J. O. Dierckx, B. L. Feringa and W. Szymanski, *Pharmaceuticals*, **2023**, *16*, 1439.
